# Doxorubicin-sensitive and -resistant colorectal cancer spheroid models: assessing tumor microenvironment features for therapeutic modulation

**DOI:** 10.3389/fcell.2023.1310397

**Published:** 2023-12-22

**Authors:** Ruben Valente, Sandra Cordeiro, André Luz, Maria C. Melo, Catarina Roma Rodrigues, Pedro V. Baptista, Alexandra R. Fernandes

**Affiliations:** ^1^ Associate Laboratory i4HB–Institute for Health and Bioeconomy, NOVA School of Science and Technology, NOVA University Lisbon, Caparica, Portugal; ^2^ UCIBIO–Applied Molecular Biosciences Unit, Department of Life Sciences, NOVA School of Science and Technology, NOVA University Lisbon, Caparica, Portugal

**Keywords:** colorectal cancer, tumor microenvironment, heterotypic spheroid models, extracellular vesicles, chemoresistance, doxorubicin

## Abstract

**Introduction:** The research on tumor microenvironment (TME) has recently been gaining attention due to its important role in tumor growth, progression, and response to therapy. Because of this, the development of three-dimensional cancer models that mimic the interactions in the TME and the tumor structure and complexity is of great relevance to cancer research and drug development.

**Methods:** This study aimed to characterize colorectal cancer spheroids overtime and assess how the susceptibility or resistance to doxorubicin (Dox) or the inclusion of fibroblasts in heterotypic spheroids influence and modulate their secretory activity, namely the release of extracellular vesicles (EVs), and the response to Dox-mediated chemotherapy. Different characteristics were assessed over time, namely spheroid growth, viability, presence of hypoxia, expression of hypoxia and inflammation-associated genes and proteins. Due to the importance of EVs in biomarker discovery with impact on early diagnostics, prognostics and response to treatment, proteomic profiling of the EVs released by the different 3D spheroid models was also assessed. Response to treatment was also monitored by assessing Dox internalization and its effects on the different 3D spheroid structures and on the cell viability.

**Results and Discussion:** The results show that distinct features are affected by both Dox resistance and the presence of fibroblasts. Fibroblasts can stabilize spheroid models, through the modulation of their growth, viability, hypoxia and inflammation levels, as well as the expressions of its associated transcripts/proteins, and promotes alterations in the protein profile exhibit by EVs. Summarily, fibroblasts can increase cell-cell and cell-extracellular matrix interactions, making the heterotypic spheroids a great model to study TME and understand TME role in chemotherapies resistance. Dox resistance induction is shown to influence the internalization of Dox, especially in homotypic spheroids, and it is also shown to influence cell viability and consequently the chemoresistance of those spheroids when exposed to Dox. Taken together these results highlight the importance of finding and characterizing different 3D models resembling more closely the *in vivo* interactions of tumors with their microenvironment as well as modulating drug resistance.

## Graphical Abstract

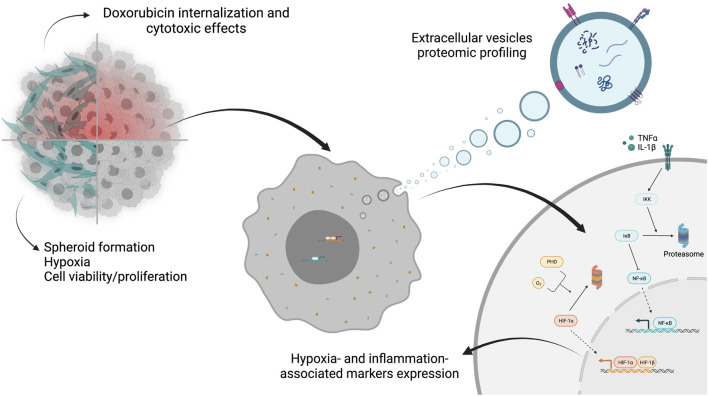

## 1 Introduction

Colorectal cancer (CRC) is one of the most prevalent and high-mortality cancers worldwide, being strongly linked to lifestyle (Keum and Giovannucci, 2019; Sawicki et al., 2021; Sung et al., 2021). Cancer cells establish crosstalk with different cellular and non-cellular components, such as stromal and immune cells, extracellular matrix (ECM), and extracellular vesicles (EVs), that together constitute the tumor microenvironment (TME) (Roma-Rodrigues et al., 2021). From all these components, cancer-associated fibroblasts (CAFs) are the most abundant cells and present a high level of heterogeneity and different functions, such as synthesis and remodeling of ECM, immunomodulation, production of growth factors, promoting angiogenesis and epithelial to mesenchymal transition (EMT) (Balkwill et al., 2012; Kalluri, 2016; Belli et al., 2018; Sahai et al., 2020; Maia and Wiemann, 2021; Mendes et al., 2021).

Cancer cells induce stromal cell migration, ECM remodeling, and expansion of the vasculature, whereas TME modulates tumor growth, invasion and metastasis, immune evasion, and response to therapy (Balkwill et al., 2012; Chen et al., 2015; Labani-Motlagh et al., 2020). During tumor growth, some tumor regions exhibit low supply of oxygen and nutrients, characterized by a hypoxic and acidic environment (Petrova et al., 2018; Sormendi and Wielockx, 2018; Aguilar-Cazares et al., 2019; Mendes et al., 2021). In these areas, tumor hypoxia and inflammation mechanisms are interconnected, being highly regulated by hypoxia-inducible factors (HIFs) and nuclear factor kappa B (NF-κB), which are responsible for activating genes associated with the promotion of tumor growth and progression and activation of cells within the TME (D’Ignazio et al., 2017; Belli et al., 2018; Petrova et al., 2018; Sormendi and Wielockx, 2018; Aguilar-Cazares et al., 2019; Roma-Rodrigues et al., 2019; Watts and Walmsley, 2019). Indeed, HIF-1 is a transcription factor composed of two subunits (HIF-1α and HIF-1β) that are constitutively expressed, but HIF-1α is only stabilized under hypoxic conditions (Petrova et al., 2018; Sormendi and Wielockx, 2018; Watts and Walmsley, 2019). HIF-1α/HIF-1β dimer activates the transcription of several target genes, which are involved in adaptive responses to hypoxia, including angiogenesis (such as *VEGFA*), glycolysis and erythropoiesis, ECM remodeling (such as *CTSD* and *MMP2*), cell survival, proliferation, apoptosis, and immune responses. (Sethi et al., 2008; Kumari et al., 2016). On the other hand, NF-κB plays an important role in several pathways, regulating downstream the expression of various genes/proteins involved in inflammation (like the pro-inflammatory cytokines IL-6 and TNF-α), immune responses, angiogenesis (such as vascular endothelial growth factor A, *VEGFA*), ECM remodeling (MMPs) and cell survival (like *HIF1A* gene) (Hoesel and Schmid, 2013; Biddlestone et al., 2015; D’Ignazio et al., 2017; Giridharan and Srinivasan, 2018).

EVs are key elements in TME since they can serve as dynamic carriers of bioactive molecules (e.g., proteins, nucleic acids and lipids), and play a crucial role in intercellular communications between cancer cells and other components of the TME. Ultimately, by promoting the autocrine and paracrine communication between several TME components, EVs can transfer oncogenic molecules (Cocucci and Meldolesi, 2015; Tkach and Théry, 2016). These can influence TME progression and metastasis formation by promoting tumor cell proliferation, angiogenesis, invasion, and evasion from the immune system, ultimately contributing to the establishment of a pro-inflammatory and immunosuppressive microenvironment (Peinado et al., 2011; Zhang and Grizzle, 2011; Becker et al., 2016; Cavallari et al., 2020; Robado de Lope et al., 2023).

**FIGURE 1:**
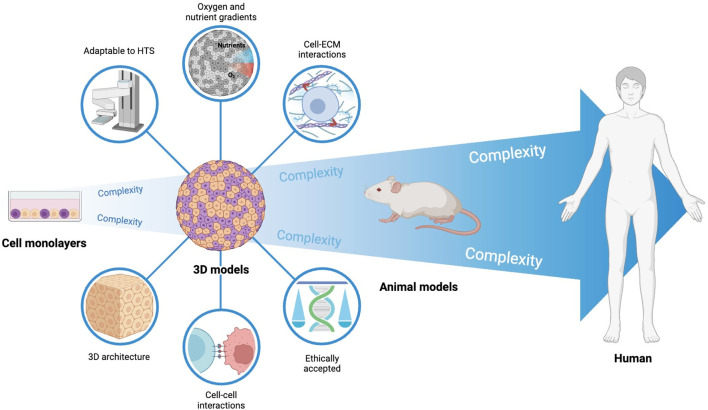
Cancer models used in preclinical research. Preclinical research in cancer heavily relies on cells cultured as monolayers. Despite being suitable for high throughput screening, these models can only mimic the *in vivo* tumor complexity to a low extent. On the opposite side, animal models have increased mimicking of the *in vivo* context, but are associated with high ethical issues, do not fully represent human context, and are not adapted to high-throughput screening. 3D human cell models and, particularly, heterotypic 3D models (tumor cells and fibroblasts used in this work) may recapitulate the *in vivo* human solid tumor phenomena to considerable levels, while being adapted to high-throughput screening necessary in pre-clinical research.

Considering the above, the development and characterization of cancer models that mimic tumors and TME *in vivo* are key to characterize the events leading to cancer progression and for the development of more efficient therapeutic strategies (Figure 1) (Hoarau-Véchot et al., 2018; Jensen and Teng, 2020; Zanoni et al., 2020). Despite having many limitations (e.g. inability to replicate the complexity of tumors and TME, which can lead to an inaccurate response of cells to therapy), two-dimensional (2D) cell cultures have been widely used because of their reproducibility, low cost, and easy manipulation (Hoarau-Véchot et al., 2018; Xin et al., 2019; Jensen and Teng, 2020; Zanoni et al., 2020). In contrast, animal models allow a systemic study of cancer mechanisms and therapy response, but are expensive, time-consuming, and raise ethical problems (Jensen and Teng, 2020; Zanoni et al., 2020).

In three-dimensional (3D) cell cultures, such as multicellular tumor spheroids and organoids, the formation of cell-to-cell and cell-matrix interactions allows a better replication of the complex *in vivo* tumor environment and constitutes a pivotal bridge between 2D cultures and animal models (Hoarau-Véchot et al., 2018; Xin et al., 2019; Jensen and Teng, 2020; Zanoni et al., 2020). In these models, cells are organized into three layers with different functions and metabolic activity: an external proliferative layer, an intermediate layer with quiescent and senescent cells, and a hypoxic and necrotic core (Hoarau-Véchot et al., 2018; Zanoni et al., 2020). Spheroids mimic tumor organization and some mechanisms, such as hypoxia and acidosis, due to the formation of gradients of nutrients, oxygen, metabolism products, and pH (Hoarau-Véchot et al., 2018; Roma-Rodrigues et al., 2019). Combination of different cell types allow further mimicking of TME (Hoarau-Véchot et al., 2018; Xin et al., 2019; Zanoni et al., 2020).

Herein, we assessed how the susceptibility or resistance to doxorubicin (Dox) of cancer cells is modulated by the presence of fibroblasts (important TME players) and impact in the response to Dox chemotherapy. We generated Dox sensitive and resistant 3D-spheroids, in homotypic or heterotypic with fibroblasts and characterized chemotherapy response via spheroid progression over time in term of size, cell number, viability, triggering of hypoxia and inflammatory response and the proteomic composition of secreted EVs.

## 2 Methods

### 2.1 Cell lines and cell culture maintenance

HCT116 colorectal carcinoma cell line (CCL-247) and Primary Dermal neonatal Fibroblasts (PCS-201-010) were obtained from American Type Culture Collection (ATCC^®^, United States). HCT116 doxorubicin-resistant cell line (HCT116-DoxR) was previously generated by culturing doxorubicin-sensitive HCT116 cells with increasing concentrations of doxorubicin (Dox, Sigma-Aldrich, United States), up to a maximum of 3.6 μM, as previously described (Pedrosa et al., 2018). Moreover, Pedrosa et al. (2018) were able to demonstrate, using Western blot, that the mechanism of resistance was due to the overexpression of P-glicoprotein (P-gP). Cells were cultured and maintained in Dulbecco’s Modified Eagle Medium (DMEM, Gibco™ by Life Technologies, United States), supplemented with 10% heat-inactivated fetal bovine serum (FBS; Gibco™ by Life Technologies, United States) or exosome-depleted heat-inactivated FBS for EVs isolation, 100 U/mL penicillin and 0.1 mg/mL streptomycin, and incubated at 37 °C, with 99% humidity and 5% (v/v) CO_2_. Fibroblast’s culture medium was additionally supplemented with 5 ng/mL fibroblast growth factor (FGF, Sigma-Aldrich, United States). For maintaining Dox selective pressure, HCT116-DoxR cells were cultivated in the presence of 3.6 μM of Dox, unless otherwise stated.

### 2.2 Spheroids formation and monitoring

Spheroids were produced using commercially available ultra-low attachment plates (BIOFLOAT™ 96-well plates, faCellitate, Germany), as described by Roma-Rodrigues et al., 2020. HCT116/HCT116-DoxR homotypic and HCT116/HCT116-DoxR-Fibroblasts heterotypic spheroids were produced, keeping initial cell seeding at 5 × 10^3^ cells per spheroid. Fibroblast seeding was made 72 h after initial cell seeding, at a HCT116/HCT116-DoxR: Fibroblasts cell ratio of 1:4, and culture medium was supplemented with 5 ng/mL of FGF.

Spheroids were monitored with CytoSMART™ Lux2 Live Cell Imager (Axion biosystems, United States) and Ti-U Eclipse Inverted microscope (Nikon Instruments, Japan).

### 2.3 Fibroblasts monitoring and cell count in 3D heterotypic spheroids

To track fibroblasts in heterotypic spheroids, fibroblasts were labeled with Cell Tracking Red Dye Kit (Abcam, United Kingdom), following manufacturer’s instructions. Labeled fibroblasts were resuspended in phenol red-free culture medium and added to each well on the third day of growth of heterotypic spheroids. Fluorescence microscopy images were taken between the 3^rd^ and 10th days of spheroid growth using CytoSMART™ Lux3 FL (Axion biosystems, United States).

### 2.4. Spheroids dissociation and cell count

To determine the cell number, spheroids were dissociated through a 30 min incubation with TrypLE™ Express (Gibco™ by Life Technologies, United States), followed by a centrifugation at 500 x g for 5 min. Viable cells were counted via Trypan Blue exclusion method.

The ratio of fibroblasts/HCT116 cells was assessed by flow cytometry. Fibroblasts were labeled as described in Section 2.3 and added to the heterotypic spheroids at the 3^rd^ day of growth. Spheroids were then disassembled as referred above, and cell populations were analyzed by Attune^®^ Acoustic Focusing Flow Cytometer (Life Technologies, Carlsbad, United States) using BL2 channel (488 nm excitation and 574/26 nm emission) (HCT116 cells = Total cells–Red fluorescent Fibroblasts) and results were processed with Attune^®^ Cytometric software. To validate total cell events, cells were also counted using trypan blue exclusion method.

### 2.5 Cell viability

Cell viability was assessed using CellTox™ Green Cytotoxicity Assay (Promega Corporation, United States), according to the manufacturer’s recommendations. CellTox™ Green dye enters cells with compromised plasma membrane, binding to their DNA, which enhances its emitted fluorescence. Considering this, an increase in the green fluorescence correlates with a decrease of cell viability. Briefly, phenol red-free culture medium supplemented with CellTox™ Green dye 1x, for 24 h. As control, spheroids were fixed with 4% paraformaldehyde (positive control). Fluorescence images were acquired with Ti-U Eclipse inverted microscope (Nikon Instruments, Japan), with a FITC filter (excitation at 465–495 nm and emission at 515–555 nm).

### 2.6 Transmission Electron Microscopy

To analyze spheroids’ internal structure and cell morphology, Transmission Electron Microscopy (TEM) was performed. TEM was provided as a service by Instituto Gulbenkian de Ciência, Portugal. Homotypic and heterotypic spheroids with 8 days of growth were fixed, dehydrated, and incubated with resin to form blocks. For each type of spheroid, sections were made at approximately one-half of the spheroids and subsequently analyzed by TEM.

### 2.7 Hypoxia detection

To evaluate hypoxia in spheroids, Image-iT™ Red Hypoxia Reagent (Invitrogen, United States) was used. Briefly, spheroids were incubated with phenol red-free DMEM with 5 µM Image-iT™ Red Hypoxia Reagent and 7.5 μg/mL Hoechst 33258, for 24 h at 37°C in a CO_2_ incubator. As a negative control, spheroids were incubated with 0.1% (v/v) DMSO, under the same conditions. Fluorescence images were acquired with Ti-U Eclipse inverted microscope (Nikon Instruments, Japan). Images of the nucleus were obtained using a DAPI filter (excitation at 340–380 nm and emission at 435–485 nm), and images of hypoxia with a G-2A filter (excitation at 510–560 nm and emission >590 nm).

### 2.8 Inflammation and hypoxia markers expression

#### 2.8.1 At gene level

Inflammation and hypoxia markers *HIF1A*, *RELA*, *VEGFA*, *MMP2*, *CTSD*, *IL6*, and *TNFA* genes expression was assessed by reverse transcription quantitative polymerase chain reaction (RT-qPCR). First, total RNA was extracted from 10 spheroids of each condition using SV Total RNA Isolation System (Promega Corporation, United States). Then, complementary DNA (cDNA) synthesis was achieved using NZY M-MulV First-Strand cDNA Synthesis Kit (nzytech, Portugal), following the manufacturer’s recommendations. cDNA amplification was performed using Rotor-Gene (Qiagen, Germany), using NZYSupreme qPCR Green Master Mix (2x) (nzytech, Portugal). The primers sequences and qPCR cycling programs used to evaluate each gene expression are described in the (Supplementary Tables S1, S2).

The RT-qPCR was used as endogenous control. Relative levels of gene expression were quantified based on the 2^−ΔΔCT^ method (Schmittgen and Livak, 2008), using the 18S ribosomal RNA (18S) gene as endogenous control (Schmittgen and Zakrajsek, 2000).

#### 2.8.2 At protein level

Levels of hypoxia- and inflammation-associated proteins were assessed by Western blot. For protein extraction, 20 spheroids from each condition were collected, protein was extracted and quantified as previously described (Sequeira et al., 2021). 20 μg of protein were separated by an 8% (for HIF-1α, Cathepsin D, and MMP2) or 12.5% (for TNF-α, IL-6, VEGFA, and NF-κB p65) acrylamide-bisacrylamide gel (SDS-PAGE) and then transferred to PVDF membranes (GE Healthcare, United States) using a semi-dry system transfer (Bio-Rad, United States).

Membranes were blocked with a 5% (w/v) non-fat milk solution in TBST (50 mM Tris-HCl, 150 mM NaCl, pH 7.5% and 0.1% (v/v) Tween 20) during 2 h, at 4°C with agitation. Then membranes were blotted with anti-HIF-1α mouse antibody (1:300); anti-Cathepsin D rabbit antibody (1:1000); anti-MMP2 mouse antibody (1:750); anti-TNF-α mouse antibody (1:1000); anti-IL-6 rabbit antibody (1:1000), anti-VEGFA rabbit antibody (1:1000), and anti-NF-κB p65 rabbit antibody (1:500), with overnight incubation at 4°C, with agitation. All primary antibodies were purchased from abcam, United Kingdom. Afterwards, membranes were washed 3x with TBST for 5 min, and incubated with the respective secondary antibody conjugated with horseradish peroxidase (HRP) (anti-mouse IgG HRP-linked, 1:3000, or anti-rabbit IgG HRP-linked antibody, 1:2000, Cell Signaling, United States) for 1 h at RT, with agitation. Signal acquisition was achieved using WesternBright™ ECL substrate (Advansta, United States) and Hyperflm ECL (GE Healthcare, United States). Images of the films were obtained with Gel Doc™ EZ Imager (Bio-Rad, United States), and proteins were quantified by densitometry using ImageJ software. β-actin expression was used as a control to normalize the results, as previously described (Choroba et al., 2023).

### 2.9 Challenging with doxorubicin

#### 2.9.1 Internalization of doxorubicin

Dox internalization was analyzed by fluorescence microscopy (Shah et al., 2017). Dox-sensitive spheroids were incubated with DMEM (without phenol red) supplemented with 8 µM Dox, and Dox-resistant spheroids were incubated with 8 µM or 120 µM Dox, for 24 and 48 h, as described by Roma-Rodrigues et al., 2020. As a control, spheroids were incubated with 0.1% (v/v) DMSO (Sigma-Aldrich, United States), under the same conditions. Fluorescence images were acquired with Ti-U Eclipse inverted microscope (Nikon Instruments, Japan), with a G-2A filter.

#### 2.9.2 Evaluation of doxorubicin cytotoxic effect

For 2D cell cultures, fibroblasts and HCT116 Dox-R cells were seeded at a density of 0.75 × 10^5^ cells/mL into 96 well-plates and incubated at 37°C and 5% (v/v) CO_2_ for 24 h. After the 24 h of incubation, culture medium was replaced by fresh medium supplemented with Dox. As negative control, 0.1% (v/v) DMSO was used. Following a 24 h or 48 h incubation, cell viability was indirectly assessed with CellTiter 96^®^ Aqueous One Solution Cell Proliferation Assay kit (Promega, Madison, United States) (Niles et al., 2008; Das et al., 2018). In metabolically active cells, mitochondrial dehydrogenases reduce 3-(4,5- dimethylthiazol-2-yl)-5-(3-carboxymethoxyphenyl)-2-(4-sulfophenyl)-2H-tetrazolium, inner salt (MTS) to formazan, whose absorbance can be measured at 490 nm in a microplate reader, Tecan Infinite M200 (Tecan, Mannedorf, Switzerland). Thus, formazan’s absorbance is directly proportional to the number of viable cells. Using Prism 8 (GraphPad software), it is possible to determine the IC_50_ (concentration that induces a 50% reduction in cell viability) of Dox for each cell line (Niles et al., 2008; Das et al., 2018).

For 3D cultures, spheroids from each culture were used on days 2, 5 and 7 of growth. At those days culture media was replaced by medium with 8 μM and 120 µM of Dox. Spheroids were then incubated for 24 h or 48 h in a humidified atmosphere at 37°C and 5% (v/v) CO_2_ and, then medium was replaced by a mixture containing the MTS reagent and DMEM medium (20:100). Spheroids were incubated for another 6 h period and transferred into a 96-well plate with flat bottom to be analysed in the microplate reader Tecan Infinite M200 (Tecan, Mannedorf, Switzerland) (Choroba et al., 2023).

### 2.10 Image analysis

The ImageJ software was used to estimate Feret’s diameter (Al-Thyabat and Miles, 2006), for fluorescence quantification and image processing, and for densitometry analysis of Western blot films, to quantify protein bands.

For fluorescence quantification, Corrected Total Cell Fluorescence (CTCF) was determined using Eq. 1. To normalize fluorescence by spheroids’ size, the CTCF values were divided by the area of the spheroids.

Eq. 1. Corrected Total Cell Fluorescence (CTCF) calculation (Rueden et al., 2017).

$$\text{CTCF}=\text{integrated density of spheroid }-\;\left(\text{area of spheroid}\times \text{background mean fluorescence}\right)$$ (1)

### 2.11 Extracellular vesicles isolation and protein content analysis

EVs were isolated between the 8^th^ and 10th days after spheroids formation using the Exoquick-TC™ kit (System Biosciences, United States), following manufacturer’s instructions. Isolated EVs were characterized via TEM, provided as a service by Instituto Gulbenkian de Ciência, Portugal and Nanoparticle Tracking Analysis (NTA).

Protein content was measured using Pierce 660™ method (Thermofisher, United States) (Antharavally et al., 2009). Subsequently, RIPA solution (25 mM Tris-HCl, 150 mM NaCl, 1% NP40, 1% Sodium deoxycholate, 0.1% SDS) was added to 100 µg of EVs/protein and incubated for 5 min at 95 °C to allow EVs lysis. The protein content of EVs was analyzed by Liquid Chromatography Mass Spectrometry (LC-MS/MS), performed as a service by LAQV, FCT-NOVA.

#### 2.11.1 Protein correlation analysis

The software STRING: functional protein association networks version 11.5 (available at https://string-db.org/) was used for protein correlation analysis, using the default settings to detect the most representative biological processes. Only biological processes with q-value <0.05 and the highest number of represented proteins were considered.

### 2.12 Statistical analysis

Statistical analysis was performed using the GraphPad Prism program (version 8.0.1). The Dunnett non-parametric two-way ANOVA test with multiple comparisons was used to compare different days in the same type of spheroid and the same day between different types of spheroids, by estimating the *p*-value. Results were considered statistically significant for *p* < 0.05.

Tukey’s Honest Significant Difference test (with an FDR of 0.05) was performed in order to compare the expression of proteins detected in EVs from the different models of spheroids studied and the respective controls of 2D models.

## 3 Results and discussion

### 3.1 Tumor spheroid formation

**FIGURE 2:**
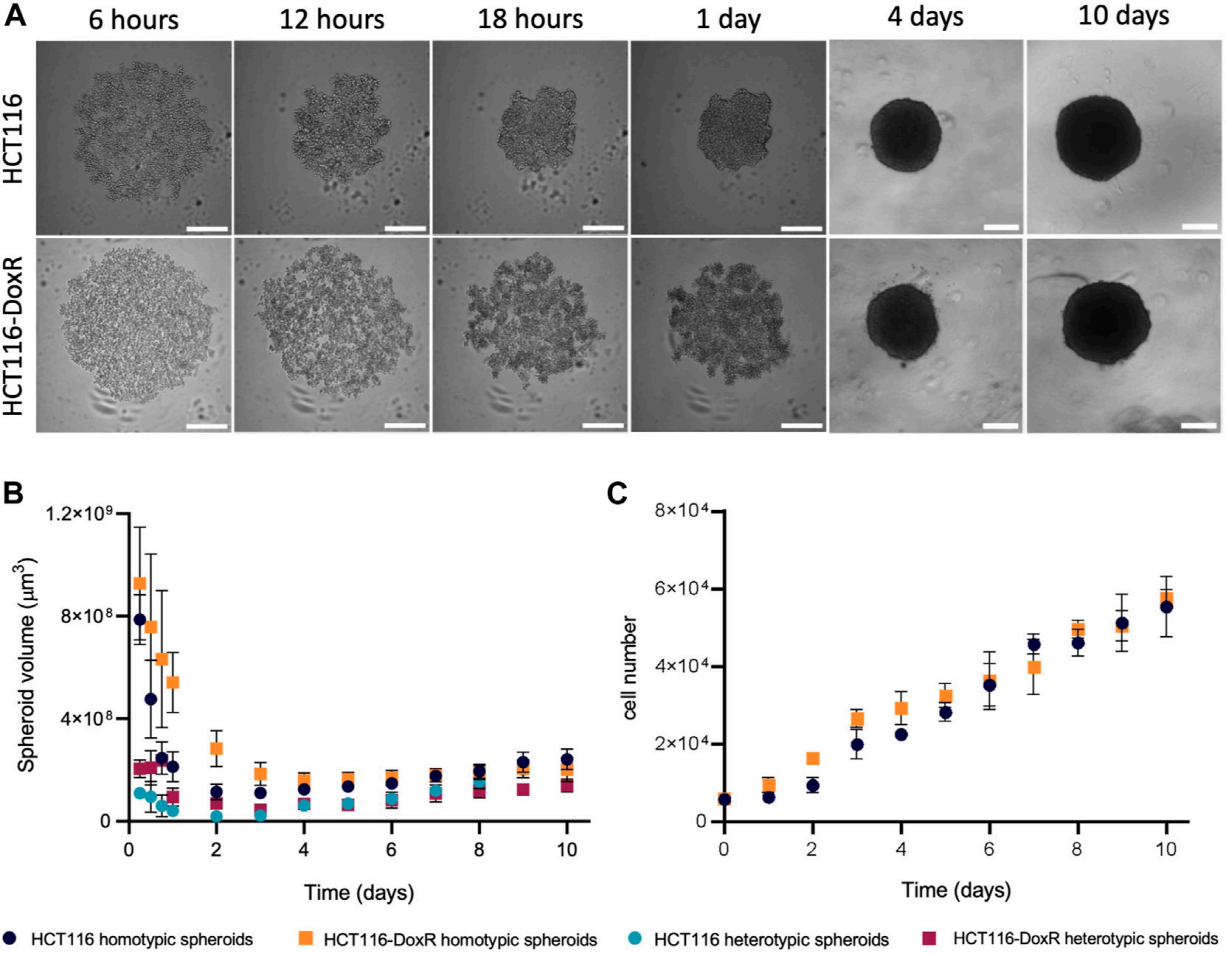
HCT116/HCT116-DoxR spheroids formation and growth over 10 days of culture. **(A)** Brightfield microscopy images of HCT116 and HCT116-DoxR homotypic spheroids; **(B)** evolution of HCT116 or HCT116-DoxR spheroids volume over 10 days of culture; and **(C)** evolution of HCT116 or HCT116-DoxR cell number over 10 days of culture. Scale bar corresponds to 300 μm. Data expressed as the mean ± SD of at least two independent assays.

The formation of tumor spheroids involves an initial step of tumor cell aggregation, followed by spheroids’ condensation (Cui et al., 2017; Han et al., 2021), which may be easily monitored over time by brightfield microscopy (Figures 2A, B; Supplementary Videos S1, S2). Dox-sensitive (HCT116) and Dox-resistant (HCT116-DoxR) spheroids follow a similar global pattern of condensation regardless of the presence (heterotypic spheroids) or absence of fibroblasts (homotypic spheroids) (Figures 2B). There are some small differences: HCT116 spheroids condense as a whole (Figures 2B; Supplementary Video S1), showing a small contraction of their volume from 15 to 2.5 × 10^8^ μm^3^, as previously demonstrated by Tartagni et al., 2023, whereas, HCT116-DoxR spheroids form several small cell aggregates close to each other, that overtime condense with less variation of their total volume (approximately from 12 to 5.8 × 10^8^ μm^3^) (Supplementary Video S2).

Also, for the first couple of days, HCT116 homotypic spheroids grow at an approximate rate of 1.8 × 10^3^ cells/day, increasing to 5.8 × 10^3^ cells/day between the 2^nd^ and the 10th day (Figures 2C). Conversely, In HCT116-DoxR homotypic spheroids, a consistent linear increase of cell density is observed (approximately 5.2 × 10^3^ cells/day). These values support the longer lag phase for the first 2 days for HCT116 homotypic spheroids (Figures 2C). Around day 9, both types of spheroids reach a 10-fold increase of cells compared to the initial seeding (Figure 2). Similar condensation patterns are observed for heterotypic spheroids, where initial cell seeding is lower, which seems to demonstrate that its growth behavior is not dependent on cell number (Figures 2C). Altogether, these data hint at the involvement of critical and specific interactions between cells and cell types during spheroid progression.

### 3.2 Fibroblast tracking

**FIGURE 3:**
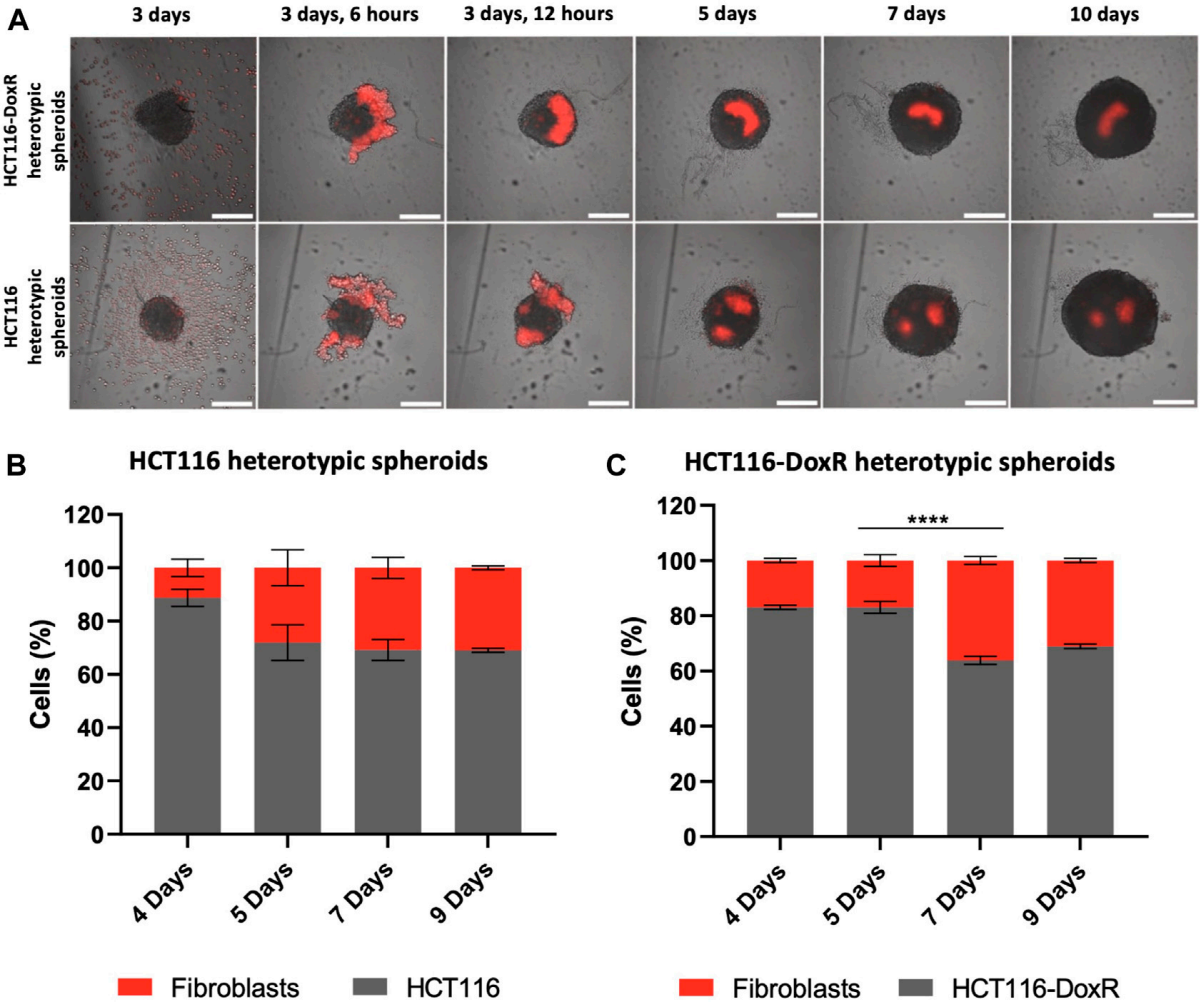
Cell tracking of fibroblasts in heterotypic spheroids. **(A)**–Fluorescence images of HCT116 and HCT116-DoxR heterotypic spheroids between days 3 and 10; Percentages of Dox-sensitive **(B)** or -resistant **(C)** cells and Fibroblasts in heterotypic spheroids at days 4, 5, 7, and 9. Fibroblasts were labeled with Cell Tracking Red Dye Kit - Longer cell staining, DMSO-free. Scale bars represent 300 μm. Data expressed as the mean ± SEM of at least two independent assays (**** *p* < 0.0001). Statistical analysis was performed by two-way ANOVA method.

In heterotypic spheroids, the interaction between fibroblasts and the already formed HCT116 or HCT116-DoxR spheroids becomes a critical point to understand the interplay between cell players. For this purpose, fibroblasts were previously stained with a cell tracker and spheroids formation monitored by fluorescence microscopy (Figure 3; Supplementary Videos S3, 4) (Massignani et al., 2010). Noteworthy, fibroblasts are not evenly distributed within HCT116 and HCT116-DoxR spheroids, but rather in clusters in a small area of the spheroid (Figures 3A). Moreover, flow cytometry data show that the ratio of fibroblasts/CRC cells is relatively stable overtime both in Dox-sensitive (Figures 3B) and resistant spheroids (Figures 3C), which highlights that the architecture of these spheroids is considerably stable.

In both heterotypic spheroids the proportion of fibroblasts show a slight increase in the first days of culture (from 17% to 36% in HCT116-DoxR and from 11% to 31% in HCT116), followed by an apparent stabilization of their proportion in different 3D cultures. As healthy cells, fibroblasts usually have a considerably low division rate when compared with HCT116 cancer cell line (Schäuble et al., 2012). Fibroblasts display doubling time of approximately 33 h (Supplementary Figure S1), whereas HCT116 and HCT116-DoxR cell lines duplicate their number in approximately 15 h and 18 h, respectively (Supplementary Figure S2, S3).

In the heterotypic spheroids under study, a different scenario is observed. The considerable differences in cell proliferation profiles of cancer and healthy cells decrease and fibroblasts’ proportions stabilize after the 5th or 7th day in HCT116 and HCT116-DoxR heterotypic spheroids, respectively **(** Figures 3B, C **)**. This indicate that, after the adaptation phase, HCT116/HCT116-DoxR cells and fibroblasts possess comparable growth kinetics, which might be associated with a modulation of fibroblasts behavior by tumor cells, as previously described for the *in vivo* cancerous growth (transition into cancer-associated fibroblasts, CAFs) (Fang et al., 2023).

### 3.3 Spheroids viability

Spheroids are important models to study cancer since their organization and structure more closely resemble *in vivo* tumors. In spheroids with more than 500 μm in diameter, it is expected the formation of three cell layers: a highly proliferative external layer, a quiescent intermediate layer, and an internal necrotic core (Zanoni et al., 2020). While homotypic spheroids presented a diameter greater than 500 μm during the 10 days studied, the heterotypic spheroids diameter only exceeds 500 μm on the 5^th^ day (as spheroids’ condensation was observed only after day 2 or 3 for HCT116 and HCT116-DoxR heterotypic spheroids, respectively) (Figures 2B). Simultaneously, the viability of the cells involved the formation of the different layers of spheroids (external, middle layer and internal necrotic core) was accessed. For homotypic spheroids, cell viability was analyzed between the 2^nd^ and 10th days of growth, while in heterotypic spheroids, cell viability was monitored from the 5^th^ day onward (diameter >500 μm).

**FIGURE 4:**
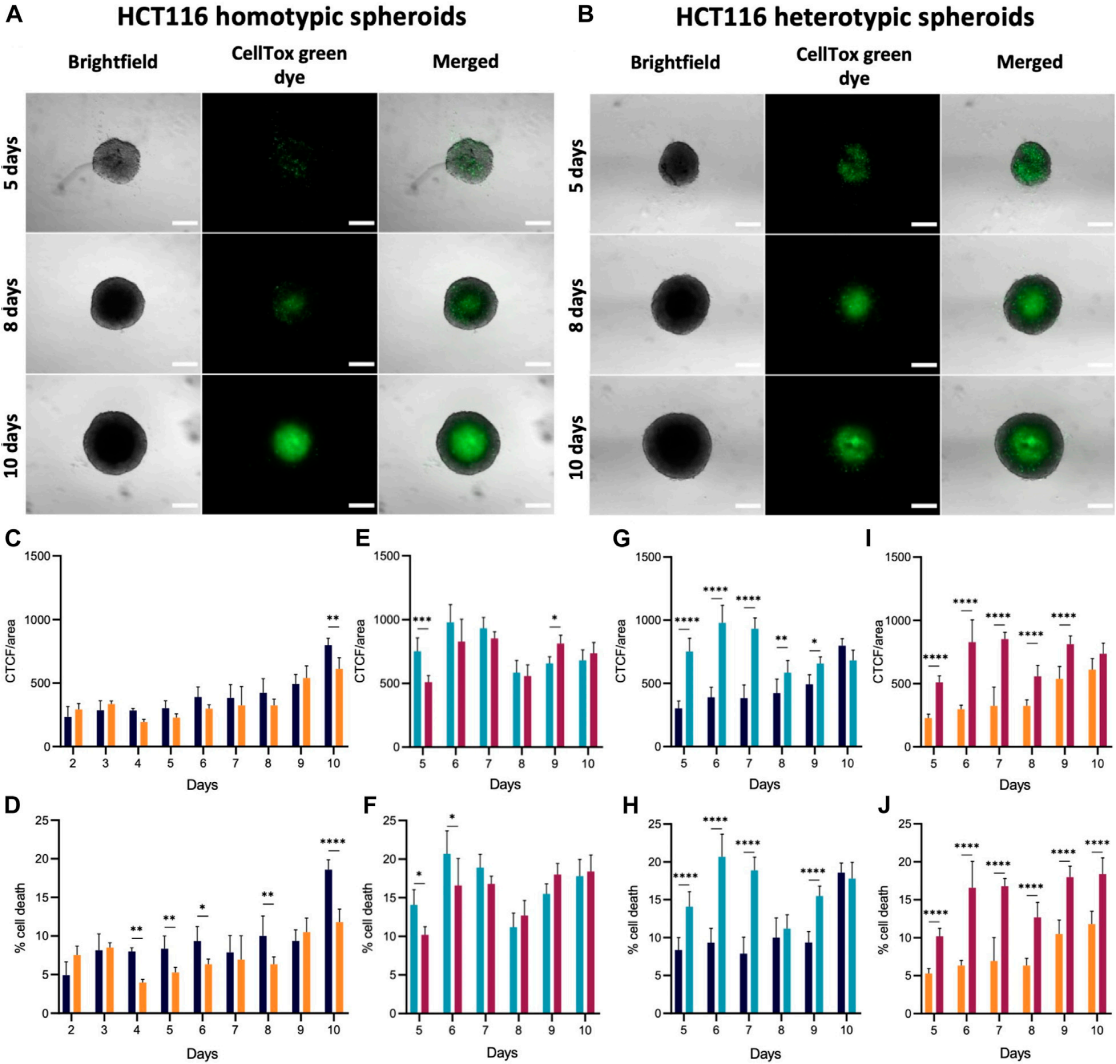
Cell viability in HCT116/HCT116-DoxR homotypic and heterotypic spheroids. **(A)** Fluorescence and brightfield microscopy images of HCT116 homotypic spheroids with 2, 6 and 10 days of growth. **(B)** Fluorescence and brightfield microscopy images of HCT116 heterotypic spheroids with 5, 8 and 10 days of growth. **(C–J)** CTCF/area values and percentage of cell death for HCT116/HCT116-DoxR homotypic and heterotypic spheroids between 2 and 10 days or between 5 and 10 days of growth, respectively. Spheroids were incubated with CellTox™ Green dye 1x for 24 h. Scale bars correspond to 300 μm. Data expressed as the mean ± SD of at least two independent assays. Statistical analysis was performed by two-way ANOVA method (*p < 0.1, **p < 0.01, ***p < 0.001, ****p < 0.0001).

In HCT116 homotypic spheroids (Figures 4C, D; Supplementary Figure S4), cell death increases over time, but most significantly from day 9 to day 10 (Figures 4C). Between days 2 and 9, approximately 5%–10% of the cells within the spheroid show some degree of impairment and/or are nonviable (Figures 4D). This percentage almost doubles on day 10, being approximately 19%. Furthermore, fluorescence images reveal an accumulation of green (membrane compromised) cells at the center of spheroids, from day 6 onwards, corresponding to the formation of a necrotic core (Figures 4A).

In HCT116-DoxR homotypic spheroids, CTCF/area values are slightly higher on day 2 and 3 than on day 4 (Figure 4C). These variations may be associated to cell death concomitant to spheroid condensation, which may be related to the increase in cell number between the day 2 and 3 (Figures 2C) since CellTox probe is added to cells 24 h prior to analysis. From day 4 onwards, fluorescence increases over time, particularly from day 8 to day 9 (Figures 4C). In HCT116-DoxR spheroids, the percentage of cell death at day 10 is lower than the corresponding values in their HCT116 counterparts (Figures 4D). This observation may be correlated to the showed accumulation of dead cells at the core of HCT116 homotypic spheroids from the sixth day onwards (Supplementary Figure S5).

Homotypic spheroids, both Dox-sensitive or resistant have similar progressions of cell death levels (Figures 4C, D). It should be noted that the corrected fluorescence values are rather similar between both cultures (Figures 4C), but cell death levels exhibit a distinct progression (Figures 4D). Globally, cell death is more marked for the HCT116 homotypic spheroids than in HCT116-DoxR homotypic spheroids, especially on the 10th day of growth.

In heterotypic spheroids, between days 5 and 7, no considerable accumulation of dead cells at the core of the spheroids is observed, but some clusters can be seen in more peripheral areas (Supplementary Figures S6, S7). As shown in Figures 3A, upon addition to the pre-formed spheroids, fibroblasts tend to form clusters, that could be related to those masses of non-viable cells. Thus, the higher intensity of fluorescence between these days might correspond to fibroblasts that did not adapt to the spheroid structure and became non-viable. Fluorescence then decreases on day 8, when the previously observed fluorescence clusters disappear and an accumulation of dead cells at the center of spheroids occurs, originating the necrotic core (Figures 4B; Supplementary Figures S7A). From day 8 onwards, there is an increase in cell death (Figures 4F).

Cell death in HCT116 heterotypic spheroids (Figures 4F) is significantly higher on day 6–7 (approximately 20%), followed by a minimum value of 11% on day 8 and later an increase up to day 10. In HCT116-DoxR heterotypic spheroids (Figures 4F), the percentage of cell death is lower than in HCT116 heterotypic spheroids, as also observed for HCT116 and HCT116-DoxR homotypic spheroids. As for HCT116 heterotypic spheroids, cell death levels are higher on days 6 and 7 (approximately 17%), then decrease on day 8 to approximately 13%, and a second increase is observed until day 10. In heterotypic spheroids (Figures 4E, F), it is also possible to note similar profiles along time for HCT116 and HCT116-DoxR, regarding corrected fluorescence intensity values as well as for percentage of cell death. By comparing homotypic with heterotypic spheroids (Figures 4G–I), large differences in CTCF/area values and in the percentage of cell death are observed. In Dox-sensitive spheroids (Figures 4G, H), these differences are more significant between days 5 and 7, with higher levels of cell death for HCT116 heterotypic spheroids. Regarding Dox-resistant spheroids (Figures 4I, J), HCT116-DoxR heterotypic spheroids present higher levels of fluorescence and cell death, on all the days studied, except on day 10, where corrected fluorescence levels are higher in homotypic spheroids.

Altogether, these data agree with what is referred in literature, that in all types of spheroids a necrotic core is formed (Hari et al., 2019; Zanoni et al., 2020). Furthermore, Dox susceptibility or resistance does not seem to have a strong influence in the formation of this necrotic core. However, the presence of fibroblasts in heterotypic spheroids appears to result in an overall cell viability decrease. Indeed, when fibroblasts are inserted into a new environment - pre-formed HCT116/HCT116-DoxR spheroids, an adaptation phase is needed and some of them will adapt to the new environment, others will not and will die. Moreover, tumor cells have high demands of nutrients and oxygen, due to the sustained activation of proliferation pathways (e.g. c-MYC) fostering the competition with fibroblasts for those requirements. The sustained activation of different cellular pathways in tumor cells also promote their metabolic adaptation, inhibition of cell death mechanisms, leading to an increased competitive behavior which trigger the elimination of rival non-adapted populations–in this case fibroblasts - via induction of apoptosis or other cell death mechanisms (Di Giacomo et al., 2017). These crosstalk between tumoral cells and fibroblasts that can adapt (usually referred to as cancer-associated fibroblasts (CAFs), allow them to activate ECM synthesis and microenvironmental remodeling, leading to stromal desmoplasia (Yang et al., 2023). CAFs have been described as having higher proliferative capabilities (Fang et al., 2023), as we also shown in our 3D models (Figure 3) where fibroblasts demonstrated proliferation rates like the ones observed for tumoral cells. This competition phenomenon that occurs between tumoral cells and fibroblasts and the adaptation of the latter to the new system, may explain the reduction of cell viability between the days 5 and 7 (corresponding to 2^nd^ and 4^th^ days after the addition of fibroblasts to the system). During this period, most fibroblasts will die and the ones that survive acquire a more aggressive phenotype, corresponding to CAFs.

### 3.4 Hypoxia

The analysis of spheroids’ viability revealed an accumulation of dead cells in the center of all the studied cultures, that most probably correspond to a necrotic and hypoxic core. To better understand this phenomenon, spatial occurrence of hypoxia was assessed using Image-iT™ Red Hypoxia Reagent, a fluorogenic compound that enables the visualization of hypoxic regions (for O_2_ concentrations below 5%) (Zhang et al., 2010). In fact, *in vivo* TME is characterized by oxygen levels between 0.3% and 4.2% (McKeown, 2014), so hypoxia regions detected using this probe might be correlated to *in vivo* data.

**FIGURE 5:**
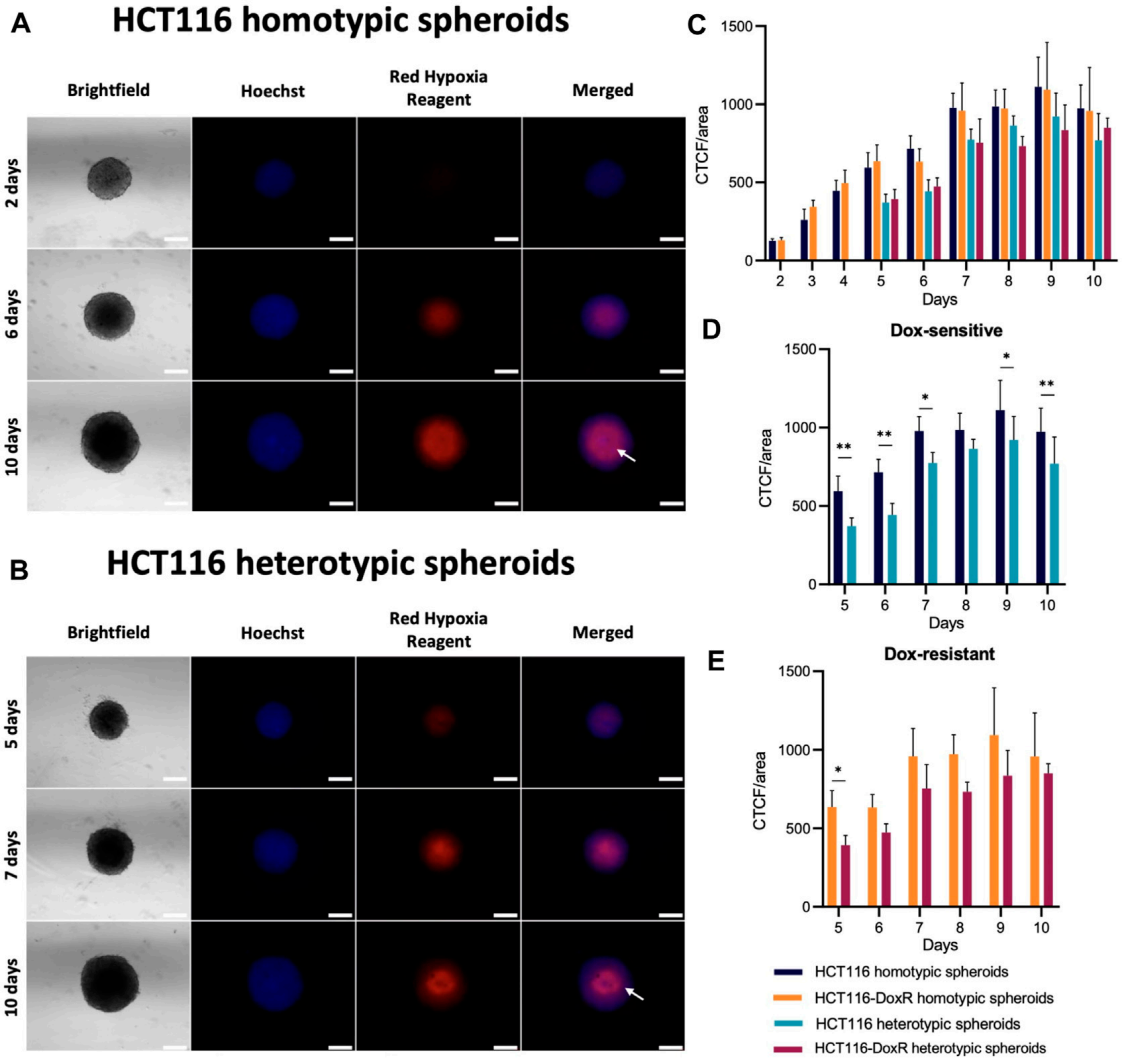
Presence of hypoxia in HCT116/HCT116-DoxR homotypic and heterotypic spheroids. **(A)** Fluorescence microscopy images of HCT116 homotypic spheroids with 2, 6, and 10 days of growth incubated with Hoechst 33258 and Image-iT™ Red Hypoxia Reagent for 24 h; **(B)** Fluorescence microscopy images of HCT116 heterotypic spheroids with 2, 6, and 10 days of growth incubated with Hoechst 33258 and Image-iT™ Red Hypoxia Reagent for 24 h; **(C)** CTCF/area values for hypoxia between 2 and 10 days of growth; **(D,E)** comparisons of the CTCF/area values for Dox-sensitive and -resistant spheroids, respectively. The white arrow on the 10th day indicates the reduction of hypoxia detected exactly in the center of the spheroid. Scale bar corresponds to 300 μm. Data expressed as the mean ± SD of at least two independent assays. Statistical analysis was performed by two-way ANOVA method (**p < 0.01).

In all types of spheroids (Figure 5), hypoxia levels increase steadily until the sixth day of growth, as expected since spheroid’ diameter increases in this period, surpassing the hypoxia threshold of approximately 400–500 μm (Riffle and Hegde, 2017; Zanoni et al., 2020). A significant rise in hypoxia levels is observed for day 6–7 (Supplementary Figure S8) that tends to steady up to the 10th day of growth. Interestingly, hypoxia at the core of homotypic spheroids occurs from 4^th^ day onwards, while the accumulation of death cells occurs from the 6^th^ day onward (Supplementary Figures S9, 10). This supports the idea that a necrotic core is formed as spheroids grow, due to the decrease of oxygen saturation and nutrient deficiency. In heterotypic spheroids, a similar pattern is observed. The development of a hypoxic core takes place on day 6, whereas the accumulation of dead cells in this region can be observed 2 days later, on the eighth day of growth (Supplementary Figure S11, S12). The hypoxia levels seem to have a similar trend over time for homotypic and heterotypic spheroids (Figures 5C). However, when comparing both types of spheroids (Figures 5C, D), higher levels of hypoxia are observed for homotypic spheroids on most days, although the differences are not as significant as those observed in cell viability (Figures 4G–J).

Interestingly, in Dox-sensitive spheroids with 10 days of growth (Figures 5A, B), it is possible to observe a reduction in hypoxia levels at the spheroids’ core. As Image-iT™ Red Hypoxia Reagent enables the detection of hypoxia in live cells, this may indicate that those regions are hollow, or only composed of dead/dying cells, thus not yielding fluorescence under hypoxia.

These results show that, although cell viability is lower in heterotypic spheroids, the levels of hypoxia tend to be higher in homotypic spheroids.

### 3.5 Transmission Electron Microscopy

During tumor progression, cancer cells are exposed to various types of stress, whether it be oxidative, metabolic, or mechanical, due to lack of nutrients, hypoxia, amongst others (Chen and Xie, 2018). To adapt and survive in this environment, maintaining high proliferation levels, cancer cells change their metabolism from oxidative phosphorylation to glycolysis (Warburg effect), even in the presence of oxygen (Petrova et al., 2018; Sormendi and Wielockx, 2018; Mendes et al., 2021). As such, during spheroid growth, it is expected that cells become organized in different layers due to oxygen, nutrients, and pH gradients (Hoarau-Véchot et al., 2018; Zanoni et al., 2020). Considering that all spheroids presented a necrotic and hypoxic core from day 8, we proceeded to assess the cell structure of these 3D models via TEM. Equatorial sectioning should allow to visualize any difference in strata of the spheroids.

**FIGURE 6:**
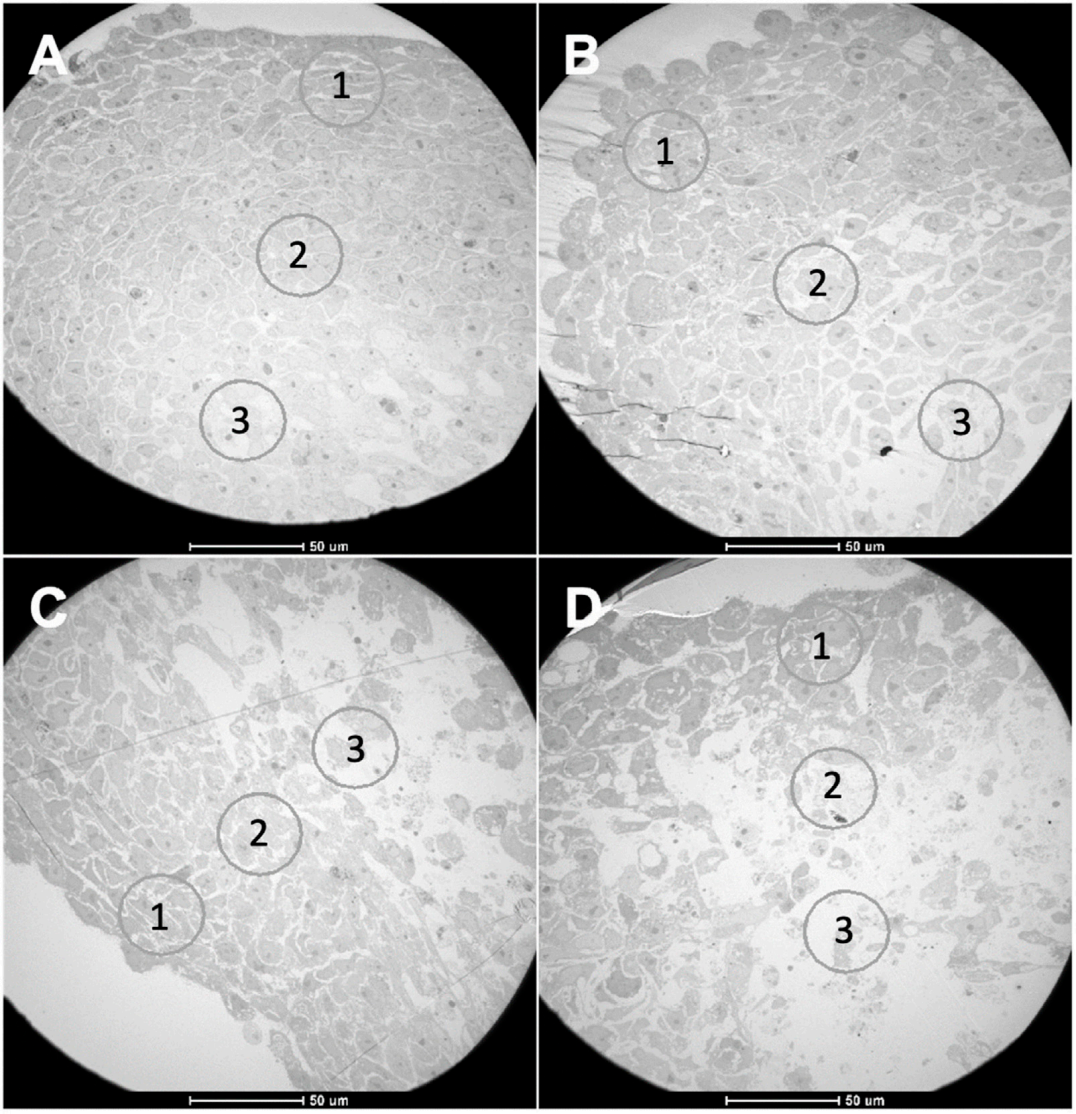
TEM images from spheroids with 8 days of growth. **(A)** HCT116 homotypic, **(B)** HCT116 heterotypic, **(C)** HCT116-DoxR homotypic, and **(D)** HCT116-DoxR heterotypic spheroids. Circles 1, 2, and 3 in each image correspond to areas from different cell layers: (1) outer layer, (2) intermediate layer, (3) core. Scale bars correspond to 50 µm.

Through the analysis of TEM images, it was possible to differentiate the three expected zones in all types of spheroids: a more external layer, an intermediate layer, and a necrotic core (Figure 6).

**FIGURE 7:**
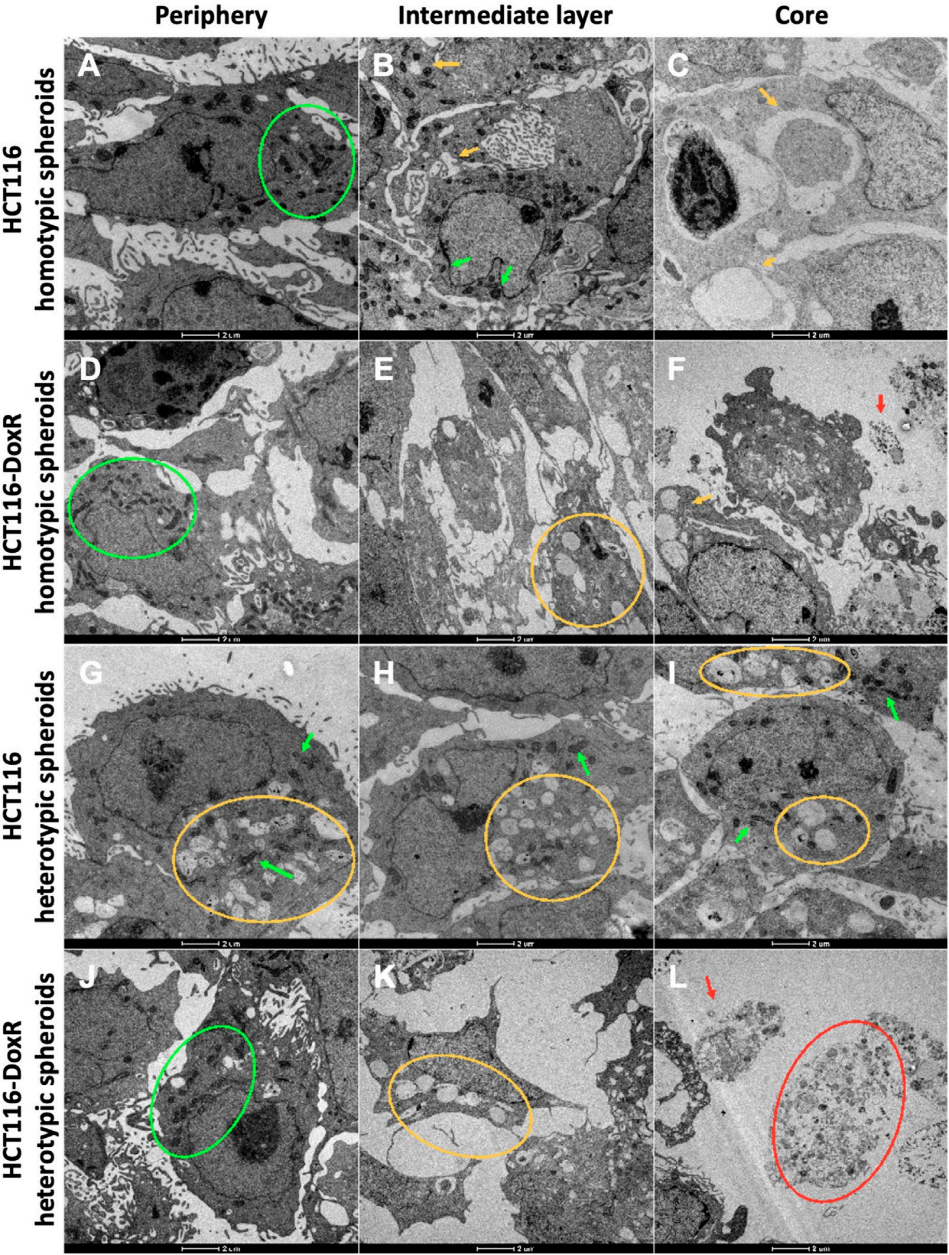
TEM images from the three cell layers of HCT116 homotypic **(A–C)**, HCT116-DoxR homotypic **(D–F)**, HCT116 heterotypic **(G–I)**, and HCT116-DoxR heterotypic **(J–L)** spheroids. For all spheroids, it was possible to identify three cell layers: **(A,D,G,J)** an outer layer, **(B,E,H,K)** an intermediate layer, and **(C,F,I,L)** a core. Green arrows and circles indicate the presence of mitochondria. Yellow arrows and circles point out vesicles. Red arrows and circles denote dead cells and cell debris. Scale bars correspond to 2 µm.

For each type of spheroid, images from the outer layer and the core were compared (Figure 7). In all types of spheroids, it was observed that the outer layer presented higher cell density, with a high number of mitochondria per cell. Mitochondria are essential organelles involved in ATP production and regulation of cell signaling, cell death, and oxidative stress, being important in the adaptation of cells to the environment (Vyas et al., 2016).

As expected, cells in the inner layer presented higher levels of stress with a lower cell density and showing numerous vesicles. These vesicular bodies may correspond to autophagic vesicles. A growing number of cellular debris is also observed that should correspond to a necrotic core.

However, in HCT116 heterotypic spheroids (Supplementary Figure S13), though cell density was higher in the outer layer for the other types of spheroids, cells in this layer already present several vesicles, and in the inner layer it was also possible to observe a high number of mitochondria per cell. Since CAFs play an important role in tumor progression and cell survival (Kalluri, 2016; Sahai et al., 2020; Maia and Wiemann, 2021), fibroblasts in HCT116 heterotypic spheroids might be improving cell survival in the inner layers. However, the same is not observed for HCT116-DoxR heterotypic spheroids (Supplementary Figure S14).

TEM images from the outer layer, intermediate layer, and core were also compared for the four types of spheroids (Figure 7). Although no significant differences were observed in cell count between Dox-sensitive and resistant homotypic spheroids at day 8 (Figures 2C), TEM micrographs demonstrated that Dox-resistant spheroids (Figures 7D–F; Supplementary Figure S15) presented lower cell density and more cell debris in the inner layers compared to their sensitive counterparts (Supplementary Figure S16). This could imply that the acquired resistance in Dox-resistant spheroids somehow affects the ability of cells to better adapt to stress, such as the observed in the inner layers of the spheroids, where hypoxia levels are higher.

The lower number of mitochondria in cells at the core could be a consequence of mitophagy - a process responsible for the degradation of mitochondria when they are damaged or dysfunctional or when cells are under stress conditions, such as hypoxia or nutrient deficiency (Vara-Perez et al., 2019; Song et al., 2022). Under hypoxic conditions, the transcription factor HIF1 activates the expression of several genes involved in the mitophagy pathway, coding for glucose transporters and glycolytic enzymes (Petrova et al., 2018; Vara-Perez et al., 2019; Watts and Walmsley, 2019). Abnormal mitophagy in cancer is associated with tumor growth and cancer metabolic reprogramming (Vara-Perez et al., 2019; Song et al., 2022).

### 3.6 Gene expression and protein levels of hypoxia and inflammation effectors

Within the TME, hypoxia and inflammation are usually associated (D’Ignazio et al., 2017). Hypoxia can promote inflammation through the activation of TME cells and induction of pro-inflammatory agents release (Biddlestone et al., 2015). On the other hand, inflammation can elevate the hypoxia levels by impairing oxygen diffusion within tumor tissue (Biddlestone et al., 2015).

**FIGURE 8:**
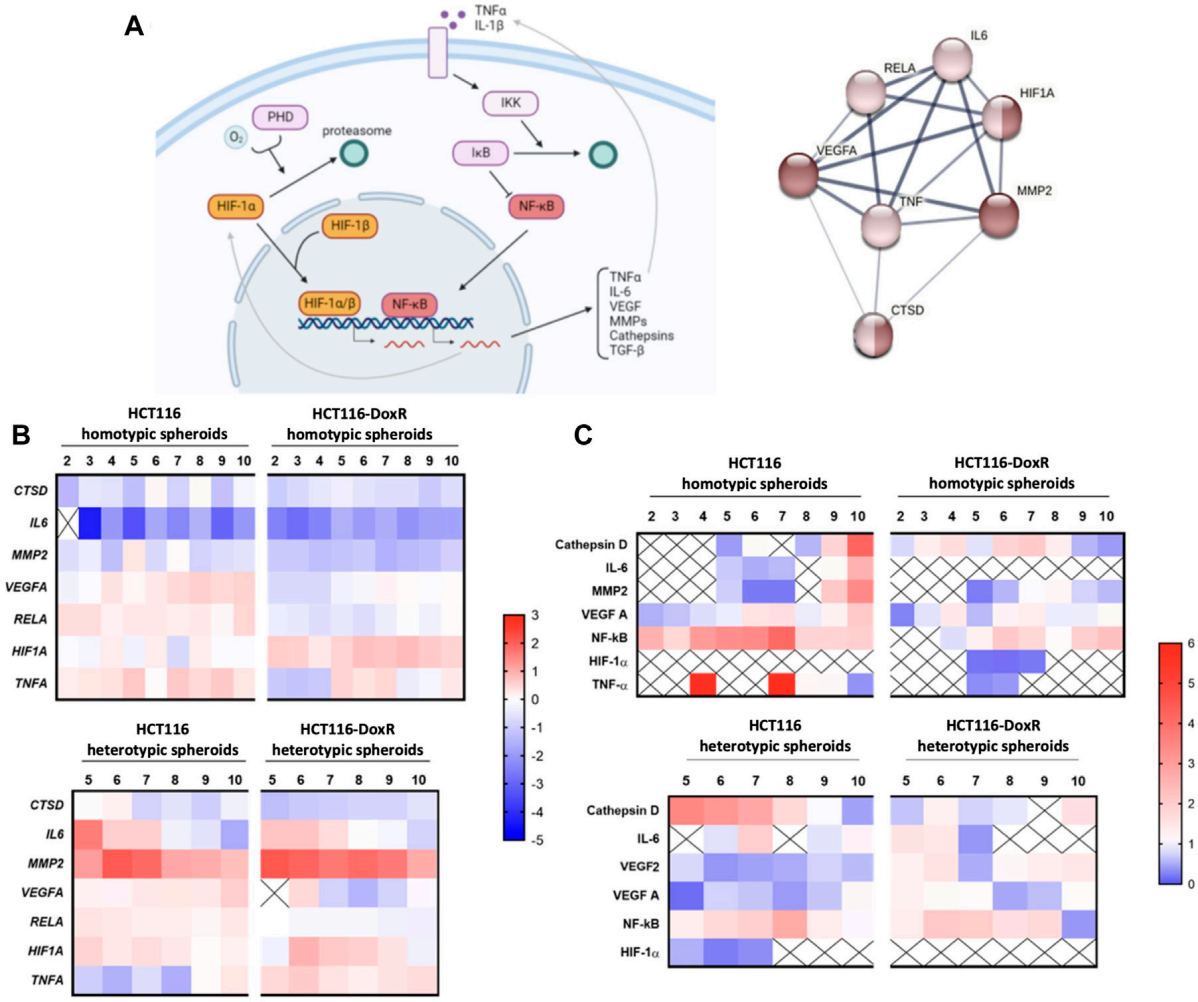
**(A)** HIF-1 and NF-κB signaling pathways of hypoxia and inflammation processes, respectively, in cancer. Dark brown nodes represent proteins involved in hypoxia response and light brown nodes identify the proteins related to inflammatory response. Image created with BioRender.com and STRING software. **(B)** Gene expression of *HIF1A*, *RELA*, *VEGFA*, *MMP2*, *CTSD*, *IL6* and *TNFA* in different types of spheroids, HCT116 homotypic and heterotypic spheroids and HCT116-DoxR homotypic and heterotypic spheroids, for 2–10 days of growth, in the case of the homotypic spheroids, and 5–10 days of growth, in the case of heterotypic spheroids. Gene expression was analyzed via the 2^−ΔΔCT^ method, and all data was normalized to the gene expression of its correspondent 2D culture (HCTT116 or HCT116-DoxR cell lines). The value 0 is considered as basal expression and the red color represents overexpression and the blue color under expression of the genes. Data is expressed as the means of at least two independent biological assays with three technical replicates each. **(C)** Protein levels of HIF-1α, TNF-α*,* NF-κB p65, IL-6, VEGFA, MMP2 and Cathepsin D in different types of spheroids, HCT116 homotypic and heterotypic spheroids and HCT116-DoxR homotypic and heterotypic spheroids, for 2–10 days of growth, in the case of the homotypic spheroids, and 5–10 days of growth, in the case of heterotypic spheroids. All data was normalized to the protein expression of its correspondent 2D culture (HCTT116 or HCT116-DoxR cell lines). Value 1 is considered as basal expression, and the red color represents overexpression and the blue color under expression of the proteins. Data is expressed as the means of at least two independent biological assays with three technical replicates each.

Therefore, we assessed the expression of genes and respective proteins involved in hypoxia and inflammation in tumor spheroids, namely the main regulator of hypoxia–HIF-1, and the main regulator of inflammation and cell survival–NF-κB, and their target genes (Figure 8).

Normalized expression (Figures 8B; Supplementary Figure S17) shows a downregulation of *CTSD*, *IL6*, and *MMP2* genes in homotypic spheroids, while in heterotypic spheroids there is an overexpression of *IL6* (between the 5th and 7th days of growth) and *MMP2* (at all the analyzed time points), which may occur due to the increased complexity of the 3D cultures after the addition of fibroblasts. Indeed, fibroblasts within TME are important modulators of cytokines and metalloproteinases release during tumor progression (Liu et al., 2019). Nevertheless, in HCT116 homotypic spheroids it is possible to observe that the remaining genes under study (*VEGFA*, *RELA*, *HIF1A and TNFA)* are overexpressed (Figures 8B; Supplementary Figure S17). The expression of the *HIF1A* gene in this type of spheroids demonstrates a first peak at the 4^th^ day of growth (corroborating what is depicted in Figure 4, that shows an increase in hypoxia). Regarding HCT116-DoxR homotypic spheroids, an overexpression of the *HIF1A* gene was detected for all time points and for *TNFA* gene the overexpression was observed after 5, 6, 7 and 10 days of growth.

*RELA*, *VEGFA* and *HIF1A* genes show similar patterns of expression in HCT116 heterotypic as in its homotypic spheroids’ counterparts, with *HIF1A* expression being slightly higher in heterotypic spheroids than in homotypic spheroids. *HIF1A* expression shows a peak at day 5 and its effect is observed at day 6, where hypoxia levels increase (Figure 5, Figures 8B).

*TNFA* gene is mostly downregulated in heterotypic spheroids. These observations may indicate that the gene that is triggering both inflammation and hypoxia signaling pathways is the *HIF1A* gene, which is able to influence the expression of NF-κB subunits and associated molecules (Biddlestone et al., 2015). On the other hand, in HCT116-DoxR heterotypic spheroids an overexpression of the *TNFA* and *HIF1A* genes occurs, but a low expression of *RELA* and *VEGFA* genes is observed. In this case, *HIF1A* gene may be negatively influencing the expression of *RELA*, preventing its expression and activation of the signaling pathway, as previously demonstrated by (Szatkowski et al., 2020). *HIF1A* exhibits a peak of expression at day 6, when hypoxia was first detected (Figures 8B; Supplementary Figure S11, S12).

The expression of the correlated proteins (as effectors of the gene expression) was also assessed through WB (Figures 8C; Supplementary Figure S18). Although hypoxia was detected in all types of spheroids by fluorescence microscopy (Figure 5; Supplementary Figures S9–S12), HIF-1α and TNF-α proteins were only detected in a few time points, which might be due to the short half-life of these proteins (Simó et al., 2012; Masoud and Li, 2015). For the other proteins, bands with the expected size were obtained (Supplementary Table S3). However, in heterotypic spheroids, only the light chain of Cathepsin D was detected.

Regarding HIF-1α and TNF-α expression, when detected, HIF-1α expression is lower than its basal expression, which may be due to its short half-life, as mentioned before. On the other hand, TNF-α only presents higher values than the basal expression in HCT116 homotypic spheroids at days 4 and 7, and a downregulation was observed at the remaining time points.

In HCT116 homotypic spheroids, two peaks of TNF-α expression levels were detected, as mentioned before, which are consistent with the progression of the NF-κB expression levels. After day 4, it is possible to observe that NF-κB expression starts to increase, as well as for VEGFA expression. This may indicate that VEGFA is one of the main target genes of NF-κB (Hu et al., 2016), since it is the first to respond to its increase. After the peak of NF-κB expression at day 7, it decreases to values near to the basal levels. On day 9, it is possible to observe an increase of VEGFA, IL-6, MMP2 and Cathepsin D levels. All these proteins play a role in the NF-κB signaling pathway (Hoesel and Schmid, 2013; D’Ignazio et al., 2017; Giridharan and Srinivasan, 2018), therefore it may be hypothesized that this increase of expression is a delayed response to the peak of NF-κB at day 7. The gene expression pattern exhibited by HCT116 homotypic spheroids shows the induction of the NF-κB signaling pathway through *TNFA*, with *VEGFA* also being expressed, which is mirrored at the protein level.

When analyzing HCT116-DoxR homotypic spheroids protein expression, TNF-α was detected at day 5 and 7, i.e. the same days for gene expression peaks. HIF-1α was detected between days 5 and 7, which also corresponds to gene expression peak. Following TNF-α and HIF-1α peaking, NF-κB and VEGFA proteins also show an increase of expression levels, confirming the interplay between these proteins. In this protein profile it is also possible to observe that Cathepsin D shows higher levels of expression at days 4, 6 and 7 of growth. Cathepsin D is associated with one of most known programmed cell death processes, apoptosis, and is also known to be an anti-angiogenic protein (Yoshida et al., 2005; Sheikh et al., 2010). *HIF1A* gene overexpression corroborates the fact that these spheroids are under hypoxia conditions (see Supplementary Figure S10, where hypoxia levels increase after 4 days of growth). Under hypoxia, Cathepsin D overexpression can be associated with the inhibition of VEGFA (Yoshida et al., 2005), which is expected to be highly overexpressed after activation of the NF-κB signaling pathway. Cathepsin D can be an inducer or inhibitor of the apoptotic process. It has been reported that, under hypoxic conditions, it mostly works as an apoptotic inducer (Sheikh et al., 2010). This may explain the accumulation of dead cells and cell debris at the core of HCT116-DoxR homotypic spheroids as of day 6 (Figures 4C, D), when the peak of Cathepsin D occurred.

In HCT116 heterotypic spheroids, Cathepsin D is the first to show a definite increase in its expression, with a peak at day 5, which then decreases to basal levels at day 9. Once again, it is possible that Cathepsin D is inhibiting the VEGFA protein, since *VEGFA* transcript is being synthesized and VEGFA protein has low levels of expression. It is also possible that Cathepsin D is promoting cell death mechanisms, as explained before, since an increase of cell death was observed (Figures 4A) 3 days after its peak and 2 days after hypoxia levels had been detected (Supplementary Figure S11). HIF-1α is detected in the time points correspondent to its gene expression peaks, after which an increase of NF-κB is noted until it reaches its peak at day 8. Interestingly, a peak of IL-6 occurs at day 7, one day before the peak of NF-κB and 2 days after its gene expression peak. These results seem to indicate that HIF-1α is the responsible for the induction of NF-κB and IL-6, and the latter being also capable of promoting the increase of NF-κB expression (Chung et al., 2017).

In HCT116-DoxR heterotypic spheroids, NF-κB is slightly overexpressed, reaching its peak between days 6–7. The same was not observed for *RELA* expression, which is always near to basal levels. It can also be observed that IL-6 and VEGFA exhibit a decreasing trend, with their peak at day 5, 1 day before the peak of NF-κB. IL-6 might be one of the main triggers of NF-κB increased expression (Chung et al., 2017), even though *HIF1A* and *TNFA* genes demonstrated to be overexpressed. MMP2 presents a peak on day 6 and another on day 10, while Cathepsin D remains close to basal levels. As Cathepsin D, MMP2 can also be associated with induction of apoptosis (Ben-Yosef et al., 2005; Seo et al., 2009). In fact, all these cultures exhibited an increase of cell death at day 8, i.e., 2 days after the peak of MMP2, which supports the influence of this protein in cell death in these spheroids.

Globally, we verified that NF-κB seemed to trigger VEGFA expression and that heterotypic spheroids express more MMP2 and IL-6 than homotypic spheroids. Concerning MMP2, it is not possible to draw many conclusions regarding protein levels, but for IL-6 protein levels in HCT116 spheroids, it was also verified that its levels decrease over time and are initially higher in heterotypic spheroids. Since stromal cells, namely fibroblasts, are the major contributors to high IL-6 and MMP2 synthesis (Liu et al., 2019), these results demonstrate that the presence of fibroblasts in the heterotypic spheroids influence protein expression. MMP2 and Cathepsin D in the different types of spheroids can promote cell death under hypoxic conditions.

It was also verified that HCT116 spheroids exhibit higher levels of expression of the effector protein related to hypoxia and inflammation. On the other hand, HCT116-DoxR spheroids show higher expression of precursor proteins. These observations suggest that when comparing the HCT116 and HCT116-DoxR spheroids the HCT116-DoxR spheroids have activated the hypoxia and inflammation pathways prior to the HCT116 spheroids.

Together, these results demonstrate the enormous potential of 3D models to recapitulate *in vivo* processes, such as hypoxia and inflammation, paramount in the TME context (D’Ignazio et al., 2017). Therefore, spheroids are great models to better understand drug resistance within tumors and the active pathways for tumor growth and development.

### 3.7 EVs protein content analysis

So far, a very good correlation has been obtained between gene and protein expression of hypoxia and inflammation biomarkers in these tumor spheroids, making them highly attractive as simplified models of the TME. Still, there are several other relevant indicators involved in cell modulation that ought to be analyzed. For example, EVs have emerged as pivotal players in intercellular communication and the regulation of diverse biological processes, influencing cellular behavior and modulating the TME (Roma-Rodrigues et al., 2014; Tao and Guo, 2020).

Prior to proteomic analysis of EVs content, EVs released by HCT116 and HCT116-DoxR both homotypic and heterotypic spheroids, as well as by 2D cultures of HCT116, HCT116-DoxR and Fibroblasts, were firstly characterized via TEM and NTA (Supplementary Figure S19, S20 and Supplementary Videos S5, S6).

**FIGURE 9:**
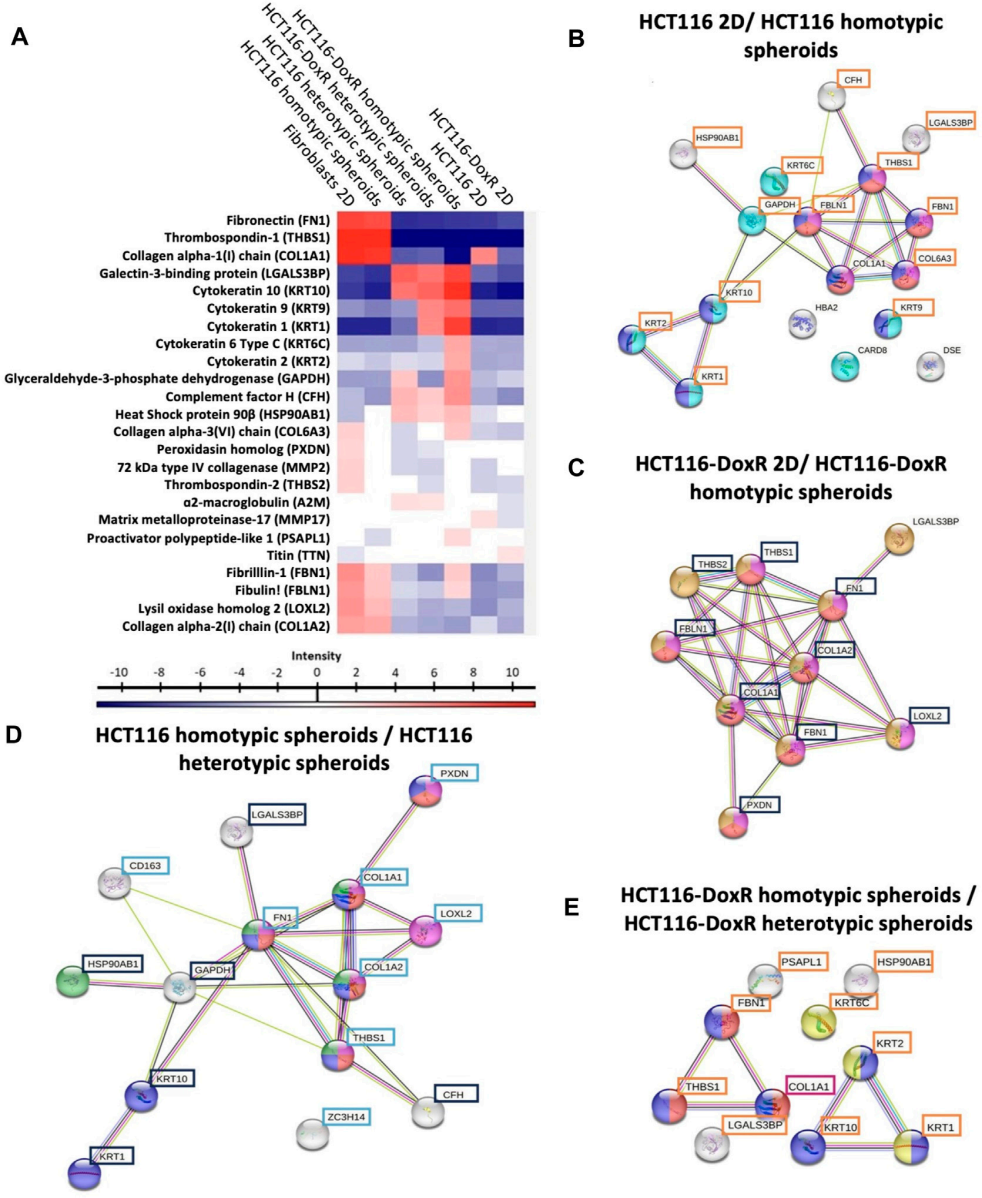
Proteomic profiling of extracellular vesicles isolated from the different culture models. **(A)** Hierarchical cluster of 24 significant proteins (multiple sample ANOVA test, based FDR 0.05) from *Homo sapiens* detected in EVs. Proteins with altered expression in EVs isolated from: **(B)** HCT116-doxR 2D culture and HCT116-DoxR homotypic spheroids; **(C)** HCT116 2D culture and HCT116 homotypic spheroids; **(D)** HCT116-DoxR heterotypic and homotypic spheroids; **(E)** and HCT116 heterotypic and homotypic spheroids. Tukey’s Honest Significant Difference test, based FDR 0.05. Nodes represent proteins and the lines connecting them indicate direct or indirect interactions. Blue nodes indicate a structural molecule activity (Molecular function–GO:0005198); red nodes indicate ECM structural constituents (Molecular function–GO:0005201); purple nodes indicate proteins with a role in ECM organization (Biological process - GO:0030198); cyan nodes indicate proteins involved in programmed cell death (Biological process–GO:0012501); orange nodes indicate collagen-containing ECM components (cellular component–GO:0062023); yellow nodes indicate keratin filament components (Cellular component–O:0045095); green nodes indicate proteins involved in PI3K-Akt signaling pathway (KEGG Pathways); and white nodes indicate proteins without a biological process associated. Proteins highlighted by blue, orange, cyan or purple boxes displayed increased levels in EVs extracted from HCT116 homotypic, HCT116-DoxR homotypic, HCT116 heterotypic or HCT116-DoxR heterotypic spheroids, respectively.

Proteomic analysis of the EVs content has identified 24 relevant proteins (Figures 9A). EVs isolated from fibroblasts monolayers show a prevalence of proteins associated with the ECM such as fibronectin (FN1) and thrombospondin 1 (THBS1), whereas HCT116-DoxR 3D culture-derived EVs have a predominance of proteins linked to the cytoskeleton, such as various cytokeratins like KRT1, KRT9, and KRT10.

A comparison between EVs isolated from 2D and 3D models (Figures 9B, C; Supplementary Table S4) revealed significant changes to protein levels, namely for proteins associated with ECM organization and cell structure, whose levels were elevated in homotypic spheroids. This might be attributed to the way cells grow - in 2D culture, cells grow on flat surfaces, which do not fully recapitulate the complex three-dimensional environment found *in vivo*. As result, certain cell structure functions, including ECM remodeling and cytoskeletal dynamics, might be altered diverge from the 3D spatial organization, with more relevant cell-cell and cell-ECM interactions. These cell-matrix and cell-cell interactions in 3D models might trigger differential expression of proteins involved in cell structure, allowing adaptation to the surrounding environment (Duval et al., 2017; Kapałczyńska et al., 2018).

A higher expression of proteins associated with cell death was observed in EVs isolated from HCT116-DoxR homotypic spheroids compared to those isolated from 2D models. This can be associated to the mechanical stress during growth in a 3D confining matrix where oxygen and nutrient gradients may lead to a suppression of cell proliferation and induction of cell death, namely by necrosis in cells located at the center of the 3D structure–necrotic core (Cheng et al., 2009; Costa et al., 2016). Also, levels of caspase recruitment domain-containing protein 8 (CARD8) appear to be reduced in EVs derived from the 3D models, which may indicate a cumulative effect on programmed cell death, since CARD8 participates in a mechanism that negatively regulates the activation of the NF-κB signaling pathway commonly involved in cell proliferation and inhibition of apoptosis (Razmara et al., 2002; Escárcega et al., 2007).

A comparison between the proteomic profiles of the EVs isolated from heterotypic and homotypic spheroids revealed altered expression of proteins associated with cell structure, either in cytoskeleton or in ECM (Figures 9D, E; Supplementary Table S5). It can be observed that a group of those structural proteins may also be involved with modulation of the PI3K-AKT pathway, a signaling cascade involved in cell growth regulation, survival, metabolism and migration, which is aberrantly activated in cancer (Fruman et al., 2017; Molinaro et al., 2019; Hopkins et al., 2020). The increased expression of proteins like THSB1 and FN1 in EVs from heterotypic spheroids could have potential indirect implications in PI3K-AKT pathway. THSB1 and FN1’s increased expression may enhance cell adhesion by integrin engagement and ECM remodeling, that can lead to an activation of PI3K and initiate downstream AKT signaling (Engelman, 2009; Horton et al., 2015; Aksorn and Chanvorachote, 2019). In contrast, a higher expression of heat shock protein HSP 90β (HSP90AB1) in EVs from homotypic spheroids suggests a potential direct modulation of the PI3K-AKT pathway in recipient cells upon EV uptake (Solit et al., 2003; Workman, 2004; Whitesell and Lindquist, 2005). This indicates that the presence of fibroblasts in 3D models, and consequent interaction with tumor cells, results in secretion of EVs that modulate the structure of the spheroid by ECM modulation and induction of cell proliferation (Supplementary Figure S21; Supplementary Table S6).

Regarding proteins present in ECM or involved in its remodeling (e.g., DSE, COL1A1, COL1A2, COL6A3, FBLN1, FBN1, FN1, PXDN, LOXL2, A2M and MMP2), these are mostly observed in heterotypic spheroids, following the order 2D homotypic cultures < homotypic spheroids < heterotypic spheroids.

Integrating these data with that from gene and protein expression, highlights *MMP2* gene with increased expression in heterotypic spheroids, strengthened by higher protein expression in HCT116-DoxR heterotypic spheroids. This may be related to high presence of fibroblasts–key players in ECM proteins secretion and ECM remodeling, namely by the secretion of MMP2 (Liu et al., 2019). Same conclusions can be taken by analyzing the Cathepsin D protein expression, which has the same cleavage role of ECM components as MMP2 (Corcoran et al., 1996). Even though the *CTSD* gene expression has been demonstrated to be low in heterotypic spheroids, CSTD protein levels are considerably elevated in HCT116 heterotypic spheroids between the 5^th^ and the 8^th^ days of growth.

A2M is a multifunctional protein that acts as a protease inhibitor (Vandooren and Itoh, 2021) and can bind to MMP2 inhibiting its function (Lindner et al., 2010; Kim et al., 2018). Its presence in EVs derived from heterotypic spheroids may be helpful to understand the considerably low MMP2 protein levels detected in HCT116 heterotypic spheroids, despite its transcript overexpression.

Although the expression levels of THBS1 and TBHS2, both angiogenesis-associated proteins (Zhang et al., 2009), were consistently higher in 3D cultures, different patterns of expression were observed. In Dox-resistant 3D models, heterotypic spheroids were associated with increased THBS1 and THBS2 levels, whereas the opposite was observed in Dox-sensitive 3D spheroids. It was also observed that these proteins always have higher levels in fibroblasts when comparing with the heterotypic spheroids. THBS1 has been reported to have an anti-angiogenic role by modulating the uptake of VEGF and antagonize its function by inhibiting the activation of the MMP9, and consequently inhibiting the mobilization of VEGF through the ECM (Zhang et al., 2009; Matuszewska et al., 2022). The regulation between those two proteins is demonstrated by the downregulation of the VEGFA protein, although *VEGFA* gene appears to be overexpressed in HCT116 heterotypic spheroids.

CD163, an immune response related protein, is highly present in HCT116 heterotypic spheroids when compared with the homotypic spheroids. CD163 is considered an inflammatory biomarker and can be found in its free form or bound to macrophages’ membrane. IL-6 is more expressed (at gene and protein levels) in HCT116 heterotypic than in HCT116 homotypic spheroids. IL-6 has been reported to increase the CD163 protein expression (Buechler et al., 2000; Calu et al., 2021).

When analyzing the proteins released by EVs in HCT116 and HCT116-DoxR spheroids, HCT116 spheroids, especially heterotypic spheroids, it is observed an increased presence of inflammation related proteins (i.e. CD163), leading to higher inflammation levels on these types of spheroids and consequently to higher expression of proteins related to this process, such as IL-6 (Figures 8C). On the other hand, HCT116-DoxR spheroids demonstrated a higher presence of anti-angiogenic proteins (such as THBS1 and THBS2). These observations are very interesting for the study of the mechanisms that may be involved in tumor chemoresistance, enabling us to understand some of the signaling pathways that may be activated in cells with acquired drug resistance.

Altogether, our data demonstrate the great potential of 3D models to recapitulate the *in vivo* tumor context and that EVs released by these models, have an important role in intercellular communication and consequent tumor progression, enabling a better understanding of the complexity of tumors and revealing new biomarkers for targeted therapy.

### 3.8 Challenging with doxorubicin

Dox inhibits topoisomerase II and intercalates with DNA base pairs, triggering apoptosis (Tacar et al., 2013). Due to its intrinsic fluorescence (Shah et al., 2017), it may be easily tracked during internalization into spheroids over time. Dox internalization was analyzed after 24 or 48 h of incubation and the effect on spheroids size was evaluated. Cell viability was determined for the 3D homotypic and heterotypic spheroids under study (Supplementary Figure S22 **)**, and the IC_50_ for 24 h or 48 h incubation of the different types of 2D cultures are shown in Table 1.

**TABLE 1:** Relative IC_50_ of Dox after 24 h and 48 h incubation with monolayer cultures. Data expressed as mean ± SEM.

Culture type	Hours	IC_50_ (µM)	References
Fibroblasts 2D	24	>120	Supplementary Figure S23
	48	12.1 ± 0.2	Choroba et al. (2023)
HCT116 2D	24	0.38	Pedrosa et al. (2018)
	48	0.5 ± 0.1	Choroba et al. (2023)
HCT116-DoxR 2D	24	>120	Supplementary Figure S24
	48	>6	Pedrosa et al. (2018)

All spheroids were incubated with 8 µM Dox, which corresponds to ∼20x the IC_50_ concentration in HCT116 2D cultures, after 48 h of incubation (Roma-Rodrigues et al., 2020). Dox-resistant spheroids were additionally incubated with 120 µM of Dox, which corresponds to ∼20 × 6 μM, that resulted in a reduction of less than 50% in HCT116-DoxR 2D cultures, after 48 h of exposure. The resistance of HCT116-DoxR cells is associated to an overexpression of ABC efflux pumps (Pedrosa et al., 2018). As such, higher intracellular levels of Dox are expected for Dox-sensitive spheroids.

**FIGURE 10:**
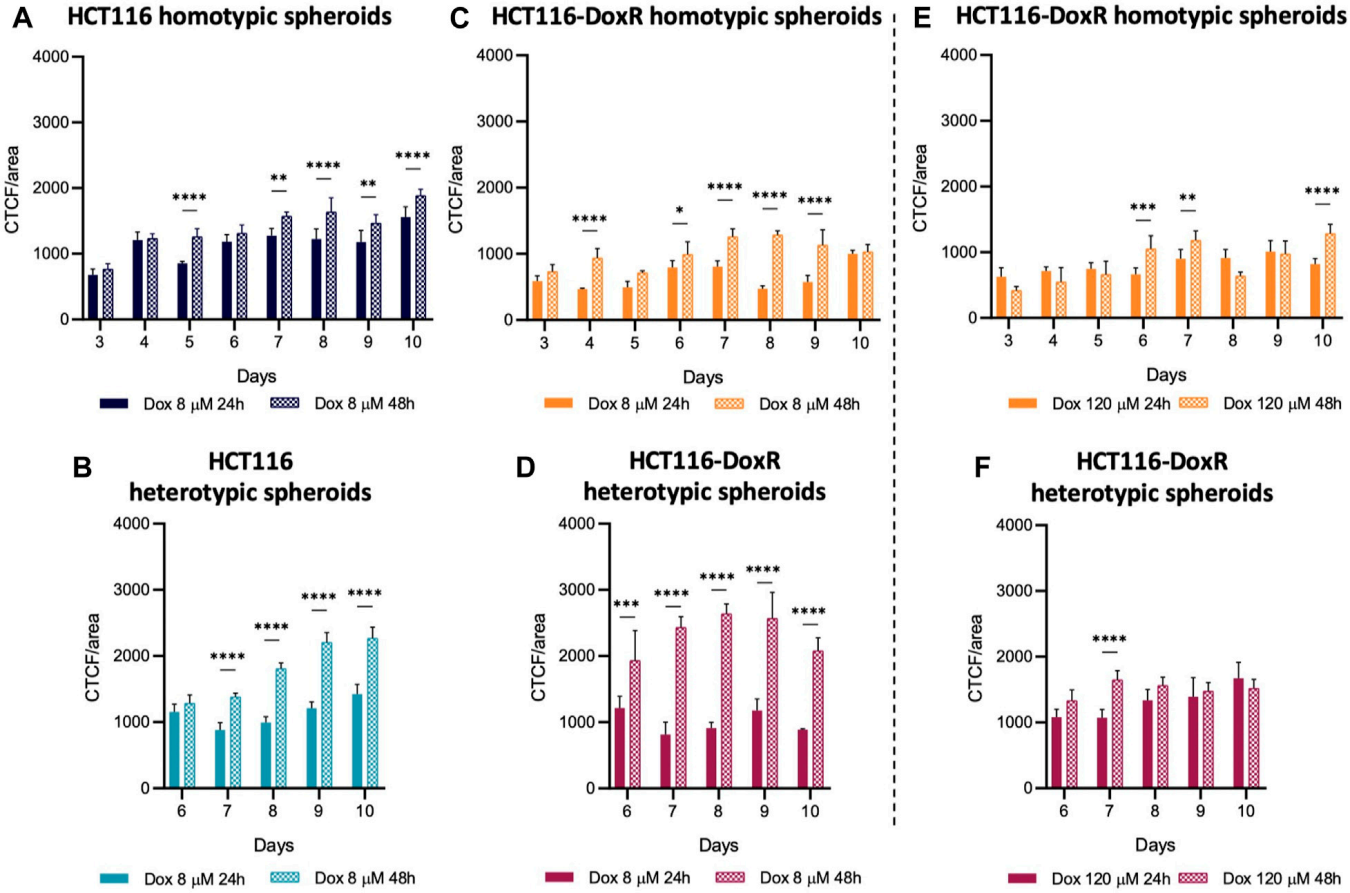
Doxorubicin internalization in spheroids. Internalization values after 24 h or 48 h in **(A)** HCT116 homotypic, **(B)** HCT116 heterotypic, **(C)** HCT116-DoxR homotypic and **(D)** HCT116-DoxR heterotypic spheroids incubated with 8 µM of Dox; and **(E)** HCT116-DoxR homotypic and **(F)** HCT116-DoxR heterotypic spheroids incubated with 120 µM of Dox. Data expressed as the mean ± SD of at least two independent assays. Statistical analysis was performed by two-way ANOVA method (*p < 0.1, **p < 0.01, ***p < 0.001, ****p < 0.0001). Data in **(E,F)** must not be directly compared with those in the other graphs, as fluorescence acquisition parameters were changed for those conditions.

In all types of spheroids incubated with 8 µM Dox (Figures 10AD), the detected fluorescence intensity was higher after 48 h than after 24 h of incubation. However, in HCT116-DoxR spheroids incubated with 120 µM Dox (Figures 10E and **-F**), these differences were less pronounced. This may indicate that, at higher concentrations, the rate of Dox uptake overtakes the rate of efflux by pumps at the cell membrane, leading to higher intracellular accumulation of the drug. In addition, all spheroids incubated with 120 µM Dox showed the formation of a round body (Supplementary Figure S25), which disappeared upon media renewal before fluorescence acquisition. This seems to indicate increased death after incubation at this higher concentration of Dox.

**FIGURE 11:**
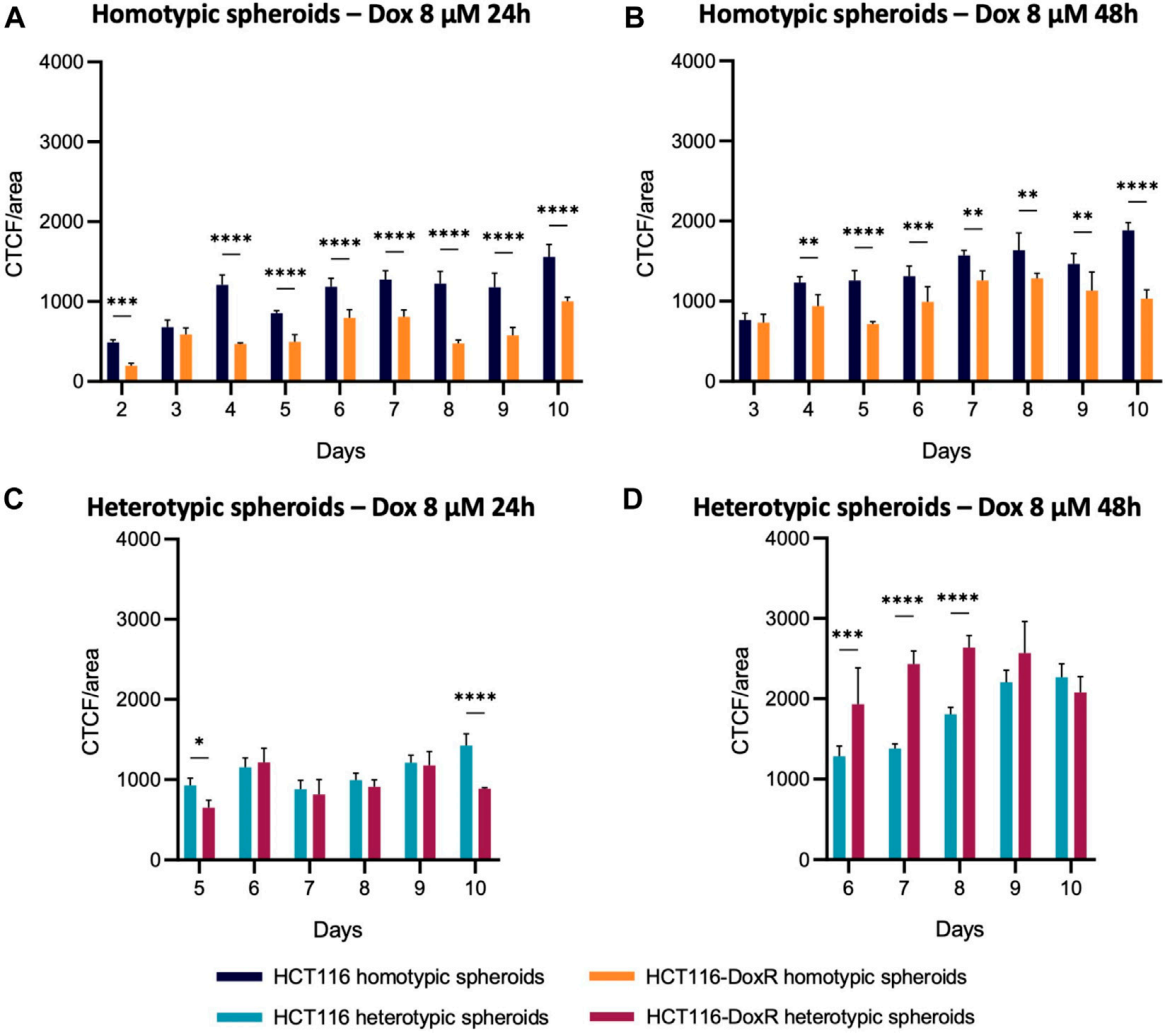
Comparison of Dox internalization between homotypic and heterotypic spheroids. CTCF/area values were compared between **(A,B)** homotypic or **(C,D)** heterotypic spheroids after 24 h or 48 h incubation with 8 µM Dox. Data expressed as the mean ± SD of at least two independent assays. Statistical analysis was performed by two-way ANOVA method (*p < 0.1, **p < 0.01, ***p < 0.001, ****p < 0.0001).

Comparing homotypic spheroids (Figures 11A, B), fluorescence levels were consistently higher in HCT116 homotypic spheroids, both after 24 and 48 h of incubation, i.e. Dox internalization was higher as expected. Fluorescence levels did not suffer a considerable variation between 24 h and 48 h incubation, which indicates that the uptake of this drug is faster in the first 24 h.

As observed in homotypic spheroids, Dox internalization levels in heterotypic spheroids (Figures 11C, D) are higher after 48 h than after 24 h, but different internalization patterns are observed. While internalization values are similar between HCT116 and HCT116-DoxR heterotypic spheroids, after 48 h those levels are significantly higher in Dox-resistant spheroids. As HCT116-DoxR cells have an overexpression of P-gP and accumulate lower amounts of Dox (Pedrosa et al., 2018; Roma-Rodrigues et al., 2020), this may lead to an increased uptake of Dox by fibroblasts, leading to an overall increased accumulation of Dox in Dox-resistant heterotypic spheroids.

A comparison between heterotypic and homotypic spheroids demonstrates that the addition of fibroblasts leads to significant changes in the accumulation of Dox, particularly in Dox-resistant spheroids **(** Figure 11; Supplementary Figure S26). Internalization levels were comparable in Dox-sensitive homotypic and heterotypic spheroids, whereas in Dox-resistant spheroids internalization levels suffered considerable increases upon the addition of fibroblasts to the 3D cultures, after incubation with 8 μM and 120 µM of Dox (Supplementary Figure S26).

**FIGURE 12:**
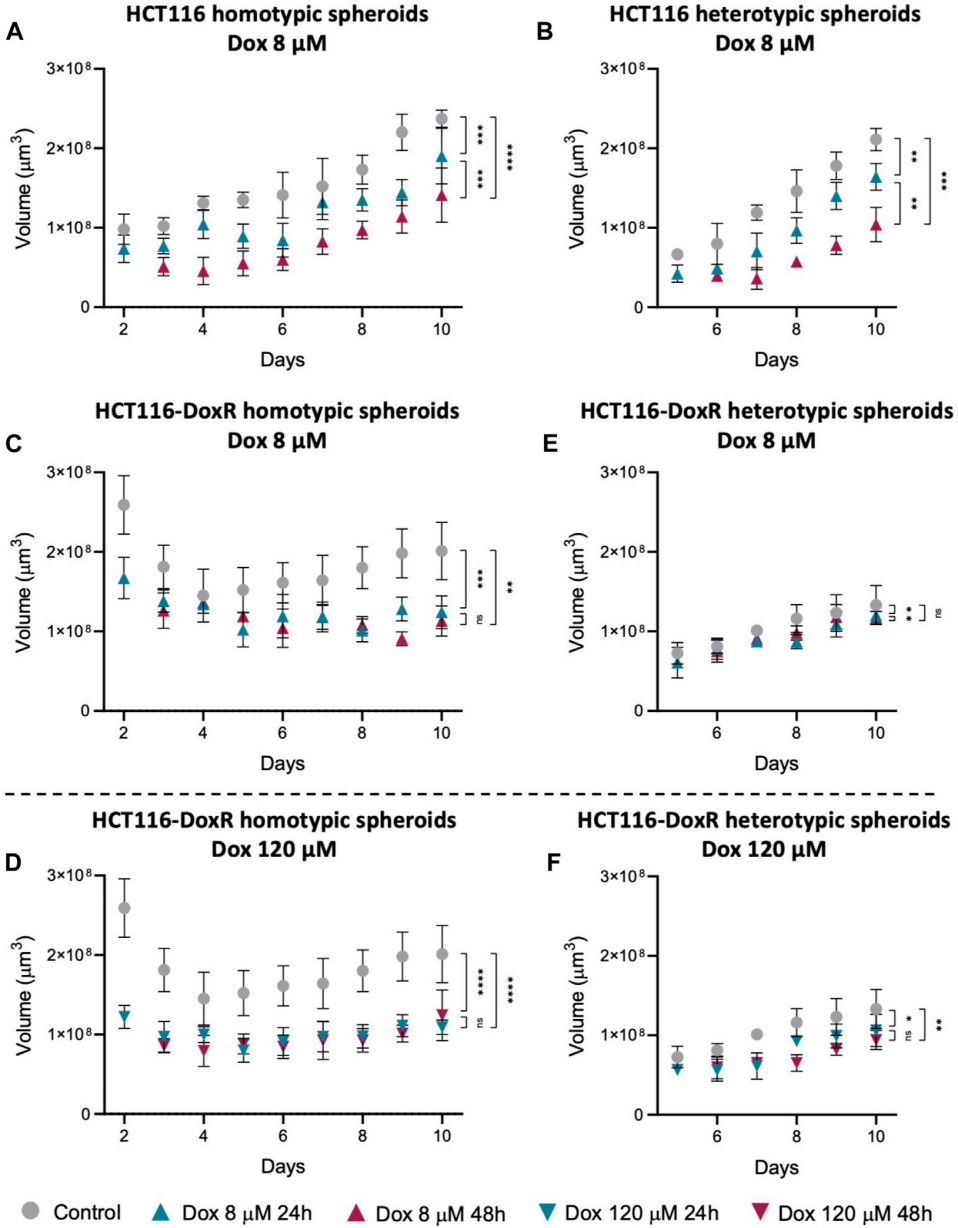
Spheroids volume after incubation with Dox. Variation in: **(A)** HCT116 homotypic spheroids incubated with 8 µM Dox; **(B)** HCT116 heterotypic spheroids incubated with 8 µM Dox; HCT116-DoxR homotypic spheroids incubated with 8 µM **(C)** or **(D)** 120 µM Dox; and HCT116-DoxR heterotypic spheroids incubated with 8 µM **(E)** or 120 µM **(F)** Dox. Data expressed as the mean ± SD of at least two independent assays. Statistical analysis was performed by ratio paired *t*-test (ns: not significant, *p < 0.1, **p < 0.01, ***p < 0.001, ****p < 0.0001).

Knowing that Dox induces cell death (Tacar et al., 2013), the variation in the total spheroid volume upon incubation with Dox was evaluated (Figure 12; Supplementary Figure S27). The reduction in volume was expected to be more pronounced after 48 h of incubation and, in the case of Dox-resistant spheroids, also higher after incubation with 120 µM Dox. Since elevated levels of Dox internalization were detected in HCT116-DoxR heterotypic spheroids, a higher variation in size was also expected, similar to Dox-sensitive spheroids. Regarding Dox-sensitive spheroids (Figures 12A, B), it was observed a decrease in volume after 24 h incubation with Dox, and this decrease was more evident after 48 h. In HCT116-DoxR homotypic spheroids (Figures 12C, D), the variation in volume was larger after incubation with 120 µM Dox, comparing with 8 µM Dox incubation, and no differences were observed between 24 or 48 h of incubation.

In HCT116-DoxR heterotypic spheroids (Figures 12E, F), although these showed higher fluorescence overall and thus higher Dox internalization, there was only a small decrease in volume after incubation with 8 or 120 µM Dox, contrary to what was expected. Fluorescence images of HCT116-DoxR heterotypic spheroids (Supplementary Figure S22) exhibit saturation of the fluorescence signal, that is neither uniform nor concentrated in the center, but rather in a few regions, which may correspond to the clusters of fibroblasts.

These results show that, as expected, Dox-sensitive spheroids are more susceptible to Dox action. Contrary to what was expected, they do not internalize more Dox. On the other hand, HCT116-DoxR heterotypic spheroids have a faster internalization of Dox, although they do not appear to be susceptible to Dox action, as seen by the small variation in volume (Figure 12). When fibroblasts are cultured in 2D, they are less sensitive to the cytotoxic action of doxorubicin (higher IC_50_ at 48 h of 12.1 µM; Table 1) compared to HCT116 2D or even HCT116-DoxR 2D cells (IC_50_ at 48 h of 0.5 µM or >6 μM, respectively). However, heterotypic spheroids with fibroblasts and tumor cells, particularly in the presence of HCT116-DoxR cells [with an overexpression of P-gP and lower accumulation of Dox (Pedrosa et al., 2018)], might have a higher internalization of Dox by fibroblasts leading to high fluorescence levels (Figures 10, 11). This also agrees with the higher growth rate of fibroblasts in heterotypic spheroids (see Table 1), resembling CAFs (Fang et al., 2023). Nevertheless, their lower sensitivity to Dox might explain the small variation in volume observed (Figure 12). However, we may not discard that when exposed to 8 µM or particularly 120 µM of Dox, HCT116-DoxR cells might trigger additional resistance mechanisms (efflux independent) that might allow higher accumulation of Dox without cytotoxicity (Wang et al., 2022).

Seemingly, the presence of fibroblasts does not affect Dox internalization in Dox-sensitive spheroids, but in Dox-resistant spheroids significantly increases Dox internalization, but without affecting Dox resistance.

**FIGURE 13:**
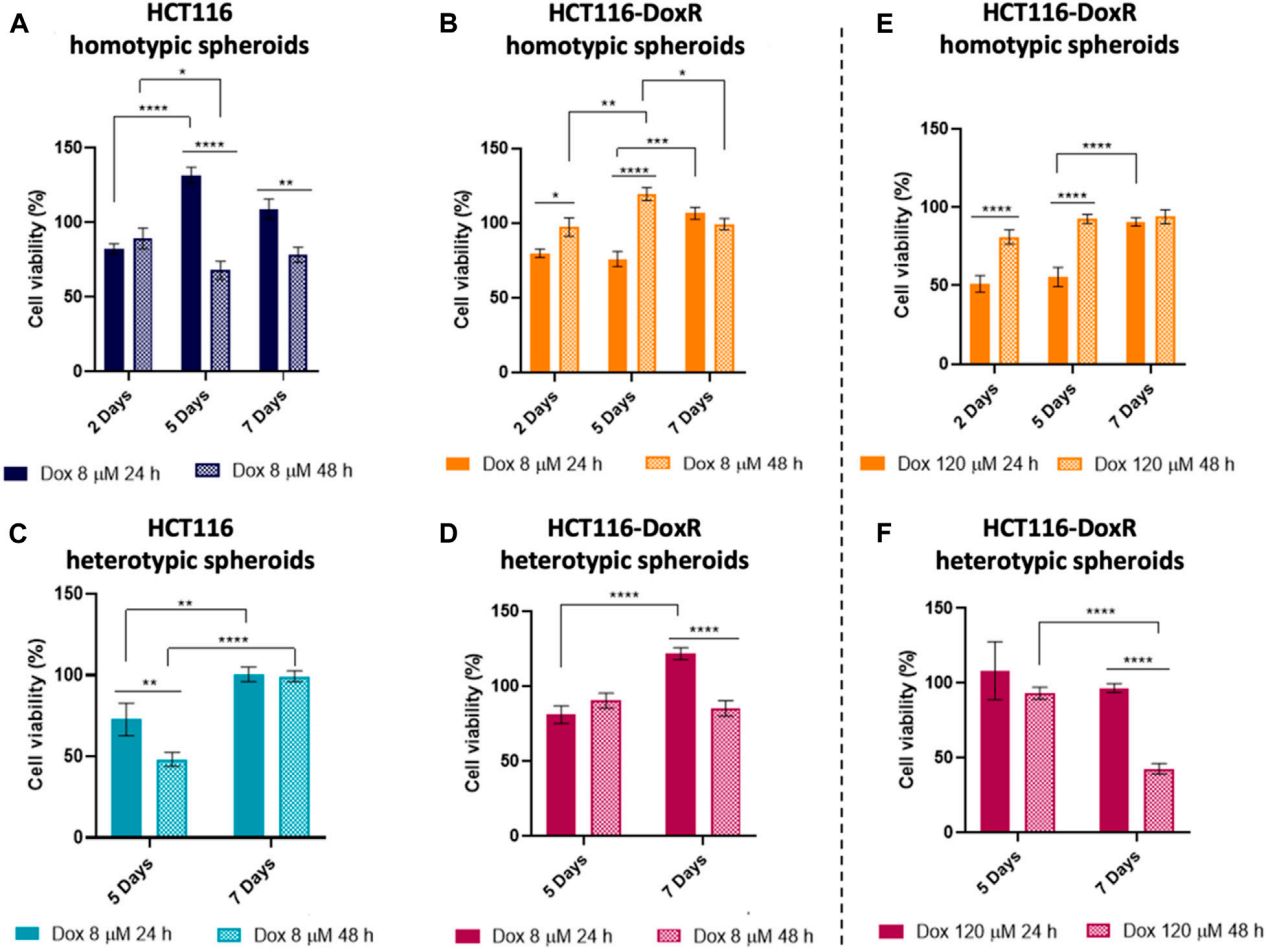
Percentage of cell viability assessed by the MTS assay of different types of spheroids after exposure to Dox for 24 h or 48 h **(A)** HCT116 homotypic, **(B)** HCT116-DoxR homotypic, **(C)** HCT116 heterotypic and **(D)** HCT116-DoxR heterotypic spheroids incubated with 8 µM of Dox; and **(E)** HCT116-DoxR homotypic and **(F)** HCT116-DoxR heterotypic spheroids incubated with 120 µM of Dox. 0.1% (v/v) DMSO was used as the vehicle control. Data are expressed as mean ± SEM of two independent assays. Statistical analysis was performed by two-way ANOVA method (* *p* < 0.1, ** *p* < 0.01, *** *p* < 0.001, **** *p* < 0.0001).

To better understand the dynamic of the different types of cells within the spheroids upon incubation with Dox, viability of the cells in the 3D models was assessed by MTS method (Figure 13) (Choroba et al., 2023).

In HCT116 homotypic spheroids incubated with 8 µM of Dox (Figures 13A), a higher loss of cell viability is observed, attaining a minimum of approximately 70% after 48 h of incubation at the 5^th^ day of growth. Interestingly, these viability values are considerable higher than those for its 2D counterpart, reinforcing the relevance of 3D models in pre-clinical research. These results agree with the data obtained for the analysis of spheroids’ volume, where the change in their volume was more pronounced after 24 h incubation with Dox (Figures 12A).

On the other hand, HCT116-DoxR homotypic spheroids (Figures 13B, E), show an increased loss of cell viability after 24 h incubation, especially for 120 µM of Dox, but after 48 h of incubation, cell viability values are close to 100% indicating a cell recovery.

Comparing both homotypic spheroids, it is possible to state that HCT116 spheroids are more susceptible to Dox action than HCT116-DoxR spheroids, especially after 48 h incubation since these last ones seem to overcome Dox action and possibly trigger additional resistance mechanisms, as mentioned above (Wang et al., 2022).

Heterotypic HCT116-DoxR spheroids (Figures 13D, F) show higher viability values upon incubation with 8 µM of Dox, compared to HCT116 heterotypic spheroids (Figures 13C). The increase of Dox concentration to 120 µM resulted in a significant reduction of cell viability, especially after 48 h incubation on the 7^th^ day (40% cell viability).

Comparing homotypic and heterotypic spheroids, in HCT116 models, the addition of fibroblasts resulted in a less noticeable difference in cell viability between the different incubation times, both at the 5th and 7th days of growth. In HCT116-DoxR spheroids, cell viability values are consistently lower in heterotypic models after 48 h incubation with 8 μM and 120 µM of Dox, being more pronounced upon incubation with 120 µM of Dox (approx. 80% at 5th day and 40% at the 7th day). Correlating these results with the variation of the spheroids’ volume and fluorescence images of Dox internalization (Figure 12; Supplementary Figure S27), the cell viability loss that is observed after 48 h incubation with 120 µM in HCT116-DoxR heterotypic spheroids at the 7th day may be due to the higher accumulation of Dox within fibroblasts, which may affect total viability but will not significantly reduce the volume of the spheroids since the periphery of the spheroids is mainly occupied by HCT116-DoxR cells, present in higher proportions compared to fibroblasts in those heterotypic spheroids.

## 4 Conclusion

The development of models that better recapitulate the TME to mimic tumor growth and progression, are critical for the characterization of the interplay between cells in this complex milieu and to evaluate the effects of TME on the response of cancer cells to therapeutic challenges. The use of 3D spheroids models enables a closer approximation to the TME and tumor organization. Herein, we characterized homotypic and heterotypic spheroids, sensitive or resistant to Dox, namely in what concerns their growth, cell viability, presence of hypoxia and inflammation, Dox internalization and extracellular vesicles content, to provide a basis for more effective translation of existing 2D culture data to the more complex models. Overall, this study reports the development of novel CRC heterotypic 3D models used as a strategy to better recapitulate the physiologic context and structure verified *in vivo.* These models allow to open new perspectives and strategies in the heterotypic 3D models context, which may be adapted to different tumor contexts. Different TME components (such as tumor-associated macrophages, tumor-infiltrating lymphocytes and myeloid-derived suppressor cells) can also be inserted to maximize the mimicking degree of the *in vivo* reality.

All types of spheroids have a similar growth progression and the susceptibility or resistance to Dox in homotypic spheroids does not affect the cell number within the spheroid. Moreover, a necrotic and hypoxic core was identified in all types of spheroids, resulting from the differential diffusion of nutrients, oxygen, and metabolic products to and from the center of these cell masses. TEM imaging further supported the presence of different strata in these spheroids, with a clear reduction of cell density towards the core with increasing amounts of cell debris, compatible with the formation of the necrotic core. Besides, the detection of low oxygen levels at the center of spheroids reinforces the idea that the formation of a necrotic core in spheroids occurs because of hypoxia. Still, there were differences between the types of spheroids in terms of hypoxia and cell viability: while homotypic spheroids tend to exhibit higher levels of hypoxia, Dox-resistance spheroids present lower levels of cell death. It is possible to claim that difference in Dox susceptibility in the CRC cell lines tested, does not influence spheroid formation and development, namely for terms of cell death and hypoxia. Also, fibroblasts in heterotypic spheroids seem to stabilize these cultures, even though with a small increase of cell death levels.

Analysis of transcript and protein levels of different hypoxia and inflammation-associated genes, indicates that the presence of fibroblasts modulates expression of some key genes/proteins, namely MMP2 and IL-6. It was also possible to verify the presence of proteins, such as MMP2 and Cathepsin D in the different types of spheroids, which may be the promotors of cell death under the observed hypoxic conditions. A deeper characterization of the secreted proteins by each cell type in heterotypic models would give greater insights about which pathways are more activated in each cell type and would enable a further understanding of the role of each cell type in tumor progression and invasion.

The overall cell to cell modulation seems to be conveyed by the protein content within EVs. In fact, several key effectors were identified pointing out to a cell-cell and cell-ECM interaction due to increase complexity of the studied spheroid models. Such observation shows that these 3D spheroid models are getting one step closer to better recapitulate the TME observed *in vivo.* Interestingly, these proteins within EVs suggests that interactions occur not only in a direct way (cell expression) but also in an indirect way, where EVs act as vehicles, carrying bioactive molecules and proteins that facilitate the crosstalk among cells, in an autocrine and paracrine way. On the other hand, this EV-mediated communication appears to have a role in other cellular processes, such as programmed cell death and modulation of signaling pathways like the PI3K-AKT pathway.

Finally, possible differences in the internalization of chemotherapeutics and cell viability after exposure to Dox were also evaluated via assessment of internalization and toxicity. As expected, the amount of drug internalization is higher for longer periods of incubation (48 h vs. 24 h), and Dox-sensitive spheroids were more susceptible to Dox action. However, contrary to what was expected, HCT116-DoxR heterotypic spheroids internalized more Dox than the other spheroids, and a small variation of the overall spheroid size (volume) is observed. Although, exposure to 120 µM Dox for 48 h induced a significant decrease of spheroids’ cell viability, an exposure for 48h to 8 µM Dox did not show any effect. This may suggest that the presence of fibroblasts is somehow affecting Dox internalization, but without affecting the ability of these spheroids to resist Dox toxicity. Such observations seem to indicate that both the presence or absence of fibroblasts and the resistance or susceptibility to Dox can affect spheroid viability, hypoxia, and Dox internalization.

## Data Availability

The datasets presented in this study can be found in online repositories. The names of the repository/repositories and accession number(s) can be found in the article/Supplementary Material.
