# Supplementary material for: Doxorubicin-sensitive and -resistant colorectal cancer spheroid models: assessing tumor microenvironment features for therapeutic modulation
Source: Front Cell Dev Biol. 2023 Dec 22;11:1310397. doi: 10.3389/fcell.2023.1310397 (PMC10771845; doi:10.3389/fcell.2023.1310397)
Supplement: Supplementary file 2 [file DataSheet2.DOCX]

Supplementary Material

**Doxorubicin-sensitive and -resistant colorectal cancer spheroid models: Assessing Tumor Microenvironment features for therapeutic modulation.**

**Ruben Valente^¥^, Sandra Cordeiro^¥^, André Luz^¥^, Maria C Melo, Catarina Roma-Rodrigues, Pedro V Baptista* and Alexandra R Fernandes***

Associate Laboratory i4HB - Institute for Health and Bioeconomy, NOVA School of Science and Technology, NOVA University Lisbon, 2819-516 Caparica, Portugal

UCIBIO – Applied Molecular Biosciences Unit, Department of Life Sciences, NOVA School of Science and Technology, NOVA University Lisbon, 2819-516 Caparica, Portugal

**^¥^Co-first authors:** these authors contributed equally to the work.

***Co-last and Corresponding authors**

**Supplementary Tables:**

**Supplementary Table 1** - List of primers forward (Fw) and reverse (Rv) used for RT-qPCR.

| Primers | | Sequence 5'-3' |
| --- | --- | --- |
| *18S* | Fw  Rv | GTA ACC CGT TGA ACC CCA TT  CCA TCC AAT CGG TAG TAG CG |
| *HIF1A* | Fw  Rv | TTG ATG GGA TAT GAG CCA GA  TGT CCT GTG GTG ACT TGT CC |
| *RELA* | Fw  Rv | ATC CCA TCT TTG ACA ATC GTG C  CTG GTC CCG TGA AAT ACA CCT C |
| *VEGFA* | Fw  Rv | ATG AAC TTT CTG CTG TCT TGG GT  TGG CCT TGG TGA GGT TTG ATC C |
| *MMP2* | Fw  Rv | AGA TCT TCT TCT TCA AGG ACC GGT T  GGC TGG TCA GTG GCT TGG GGT A |
| *CTSD* | Fw  Rv | ACC TGA ATG TCA CCC GCA AG  TCT CAC AGG GGA TCA TGT ACT CG |
| *IL6* | Fw  Rv | GGT ACA TCC TCG ACG GCA TCT  TCT TTG CTG CTT TCA CAC AT |
| *TNFA* | Fw  Rv | CCA GGC AGT CAG ATC ATC TTC TC  TAT CTC TCA GCT CCA CGC CA |

**Supplementary Table 2.** RT-qPCR cycling programs.

| Gene | Initial denaturation | Denaturation | Annealing | Extension | Number of cycles | Final extension |
| --- | --- | --- | --- | --- | --- | --- |
| *HIF1A* | 95 °C, 2 min | 95 °C, 20 s  95 °C, 20 s | 50 °C, 10 s  58 °C, 10 s | 72 °C, 20 s  72 °C, 20 s | 10  30 | --------- |
| *RELA* | 95 °C, 5 min | 95 °C, 30 s | 58 °C, 20 s | 72 °C, 20 s | 30 | --------- |
| *VEGFA* | 95 °C, 5 min | 95 °C, 30 s | 60 °C, 15 s | 72 °C, 15 s | 35 | 72 °C, 5 min |
| *MMP2* | 95 °C, 3 min | 95 °C, 30 s | 62 °C, 15 s | 72 °C, 10 s | 30 | --------- |
| *CTSD* | 95 °C, 3 min | 95 °C, 30 s | 62 °C, 15 s | 72 °C, 10 s | 30 | --------- |
| *IL6* | 95 °C, 5 min | 95 °C, 20 s  95 °C, 20 s | 50 °C, 10 s  53 °C, 10 s | 72 °C, 20 s  72 °C, 20 s | 10  30 | --------- |
| *TNFA* | 95 °C, 5 min | 95 °C, 20 s  95 °C, 20 s | 50 °C, 10 s  53 °C, 10 s | 72 °C, 20 s  72 °C, 20 s | 10  30 | --------- |

**Supplementary Table 3**. Proteins bands obtained through WB technique for each protein at 2-10 days of growth of homotypic spheroids and 5-10 days of growth of heterotypic spheroids (*n* = 2): 120 kDa for HIF-1α, 64 kDa for NF-κB p65, 40 kDa for VEGFA, 75 kDa for MMP2, 44 kDa for Cathepsin D (being also possible to detect the light chain at 15 kDa), 21 kDa for IL-6 and 26 kDa for TNFα.

|  | Proteins/  Days | | 2 | 3 | 4 | 5 | 6 | 7 | 8 | 9 | 10 |
| --- | --- | --- | --- | --- | --- | --- | --- | --- | --- | --- | --- |
| HCT116 homotypic spheroids | **NF-κB** |  | |  |  |  |  |  |  |  |  |
|  | **VEGFA** |  | |  |  |  |  |  |  |  |  |
|  | **MMP2** | - | | - | - |  |  |  | - | - | - |
|  | **Cathepsin D** | - | | - | - |  |  | - | 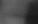 | 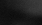 | 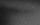 |
|  | **IL-6** | - | | - | - |  |  |  | - |  | 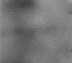 |
|  | **HIF-1α** | - | | - | - | - | - | - | - | - | - |
|  | **TNF-α** | - | | - | 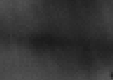 | - | - | 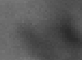 | 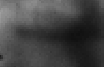 | 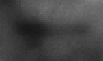 | 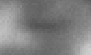 |
| HCT116 –DoxR homotypic spheroids | **NF-κB** | - | | - |  |  |  |  |  |  |  |
|  | **VEGFA** |  | |  |  |  |  |  |  |  |  |
|  | **MMP2** | - | | - | - |  |  |  |  |  |  |
|  | **Cathepsin D** | 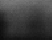 | | 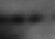 | 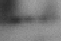 |  |  |  |  |  |  |
|  | **IL-6** | - | | - | - | - | - | - | - | - | - |
|  | **HIF-1α** | - | | - | - | 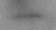 | 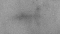 | 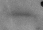 | - | - | - |
|  | **TNF-α** | - | | - | - | 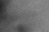 | 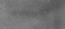 | - | - | - | - |
| HCT116 heterotypic spheroids | **NF-κB** | - | | - | - | 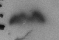 | 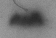 | 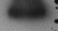 |  |  |  |
|  | **VEGFA** | - | | - | - | 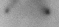 | 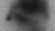 |  |  |  |  |
|  | **MMP2** | - | | - | - |  |  |  |  |  |  |
|  | **Cathepsin D** | - | | - | - |  |  |  |  |  |  |
|  | **IL-6** | - | | - | - | - |  |  | - |  |  |
|  | **HIF-1α** | - | | - | - | 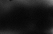 | 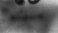 | 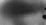 | - | - | - |
|  | **TNF-α** | - | | - | - | - | - | - | - | - | - |
| HCT116-DoxR heterotypic spheroids | **NF-κB** | - | | - | - |  |  |  |  |  |  |
|  | **VEGFA** | - | | - | - |  |  |  |  |  |  |
|  | **MMP2** | - | | - | - |  |  |  |  |  |  |
|  | **Cathepsin D** | - | | - | - |  |  |  |  | - |  |
|  | **IL-6** | - | | - | - |  |  |  | - | - | - |
|  | **HIF-1α** | - | | - | - | - | - | - | - | - | - |
|  | **TNF-α** | - | | - | - | - | - | - | - | - | - |

**Supplementary Table 4.** List of proteins with significant differential expression between 2D models and homotypic spheroids of HCT116/HCT116-DoxR derived EVs. Expression was assessed using Tukey's test (FDR of 0.05). Positive values indicate an increased expression in EVs from 2D models and Negative values indicate an increased expression in EVs from homotypic spheroids.

| Protein | Abbreviation (ID Uniprot) | Description | T-student Test Difference | |
| --- | --- | --- | --- | --- |
|  |  |  | (HCT116-DoxR 2D –HCT116- DoxR homotypic spheroids) | (HCT116 2D –HCT116 homotypic spheroids) |
| Galectin-3-binding protein | LGALS3BP (Q08380) | Glycoprotein that interacts with various molecules in the extracellular matrix and cell surface. Can be involved in in immune regulation, inflammation, cell adhesion, and tissue remodeling. | -4.33 | 1.35 |
| Hemoglobin subunit α | HBA2 (P69905) | Plays a crucial role in oxygen transport. | 3.03 | - |
| Collagen α-1(I) chain | COL1A1 (P02452) | Key component of the extracellular matrix (ECM) with diverse functions, contributing to tissue strength, wound healing, bone formation, and cell signaling. | 2.16 | -1.82 |
| Dermatan-sulfate epimerase | DSE (Q9UL01) | Enzyme involved in the biosynthesis of dermatan sulfate, a glycosaminoglycan with roles in tissue organization, function, and biological signaling. | 3.88 | - |
| Caspase recruitment domain-containing protein 8 | CARD8 (Q9Y2G2) | Plays important roles in various cellular processes, particularly in the regulation of inflammation and cell death. It is known to Inhibit NF-κB activation. | 0.68 | - |
| Collagen α-2(I) chain | COL1A2 (P08123) | Crucial component of collagen type I. Collagen type I is a fibrous, structural protein found in various connective tissues, providing strength, support, and flexibility to ECM. | - | -1.82 |
| Thrombospondin-2 | THBS2 (P35442) | Adhesive glycoprotein that mediates cell-to-cell and cell-to-matrix interactions essential in tissue development, ECM organization and wound healing. | - | -1.51 |
| Fibronectin | FN1 (P02751) | Essential ECM protein involved in cell-matrix interactions. | - | -7.1 |
| Thrombospondin-1 | THBS1 (P07996) | Adhesive glycoprotein that mediates cell-to-cell and cell-to-matrix interactions essential in tissue organization, repair, and homeostasis. | -1.01 | -6.69 |
| Fibulin-1 | FBLN1 (P23142) | Component of the ECM and is involved in the assembly and stabilization of various ECM structures. Its interactions with other ECM components and cell surface receptors make it a critical player in tissue development, homeostasis, and repair. | -1.73 | -2.33 |
| Fibrillin-1 | FBN1 (P35555) | Glycoprotein that plays a critical role in the formation and maintenance of elastic fibers in connective tissues. It is a major component of the ECM. | -1.35 | -2.33 |
| Peroxidasin homolog | PXDN (Q92626) | Displays peroxidase activity and is likely to participate in H_2_O_2_ metabolism. Plays a role in extracellular matrix formation | - | -0.86 |
| Lysyl oxidase homolog 2 | LOXL2 (Q9Y4K0) | Enzyme involved in ECM maturation through crosslinking of collagen and elastin fibers | - | -2.18 |
| Complement factor H | CFH (P08603) | Glycoprotein with an essential role in maintaining a well-balanced immune response by modulating complement activation. | -0.71 | - |
| Collagen α-3(VI) chain | COL6A3 (P12111) | Critical component of collagen type VI. Its presence in the ECM provides support, elasticity, and stability to tissues, and its functions are essential for tissue development, homeostasis, and repair. | -1.99 | - |
| Glyceraldehyde-3-phosphate dehydrogenase | GAPDH (P04406) | Important in cellular metabolism and regulation. Its involvement in glycolysis ensures the production of energy, while its non-metabolic functions contribute to cellular homeostasis and signaling. | -1.15 | - |
| Cytokeratin 6 Type C | KRT6C (P48668) | Plays important roles in maintaining the structural integrity of epithelial tissues and performing specialized functions. | -3.69 | - |
| Cytokeratin 1 | KRT1 (P04264) | Plays fundamental roles in the structural integrity, differentiation, and barrier function. May regulate the activity of kinases such as PKC and SRC. | -3.14 | - |
| Heat shock protein 90β | HSP90AB1 (P08238) | Molecular chaperone that promotes the maturation, structural maintenance and proper regulation of specific target proteins involved for instance in cell cycle control and signal transduction. Undergoes a functional cycle that is linked to its ATPase activity. | -1.35 | - |
| Cytokeratin 10 | KRT10 (P13645) | Plays crucial roles in the structural integrity, differentiation, and barrier function of the epidermis. | -3.08 | - |
| Cytokeratin 9 | KRT9 (P35527) | Plays fundamental roles in the structural integrity, differentiation, and barrier function. | -4.06 | - |
| Cytokeratin 2 | KRT2 (P35908) | Intermediate filament protein that plays essential roles in providing structural support, promoting cell differentiation, and contributing to tissue organization in epithelial cells. | -4.99 | - |

**Supplementary Table 5.** List of proteins with significant differential expression between homotypic and heterotypic spheroids of HCT116/HCT116-DoxR derived EVs. Expression was assessed using Tukey's test (FDR of 0.05). Positive values indicate an increased expression in EVs from homotypic spheroids and Negative values indicate an increased expression in EVs from heterotypic spheroids.

| Protein | Abbreviation (ID Uniprot) | Description | T-student Test Diference | |
| --- | --- | --- | --- | --- |
|  |  |  | (HCT116-DoxR homotypic spheroids –HCT116-DoxR heterotypic spheroids) | (HCT116 homotypic spheroids –HCT116 heterotypic spheroids) |
| Cytokeratin 6 Type C | KRT6C (P48668) | Plays important roles in maintaining the structural integrity of epithelial tissues and performing specialized functions. | 2.99 | - |
| Collagen α-2(I) chain | COL1A2 (P08123) | Crucial component of collagen type I. Collagen type I is a fibrous, structural protein found in various connective tissues, providing strength, support, and flexibility to ECM. | - | 2.74 |
| Scavenger receptor cysteine-rich type 1 protein M130 | CD163 (Q86VB7) | Cell surface receptor with essential roles in immune regulation, inflammation resolution, tissue repair, and iron metabolism. | - | 3.01 |
| Zinc Finger CCCH-Type Containing 14 | ZC3H14 (G3V4R5) | RNA-binding protein that is involved in the regulation of post-transcriptional gene expression. | - | 2.55 |
| Collagen -1(I) chain | COL1A1 (P02452) | key component of the extracellular matrix (ECM) with diverse functions, contributing to tissue strength, wound healing, bone formation, and cell signaling. | - 2.02 | 2.87 |
| Fibronectin | FN1 (P02751) | Essential ECM protein involved in cell-matrix interactions | - | 7.66 |
| Thrombospondin-1 | THBS1 (P07996) | Adhesive glycoprotein that mediates cell-to-cell and cell-to-matrix interactions essential in tissue organization, repair, and homeostasis. | 1.52 | 5.52 |
| Peroxidasin homolog | PXDN (Q92626) | Displays peroxidase activity and is likely to participate in H_2_O_2_ metabolism. Plays a role in extracellular matrix formation | - | 1.41 |
| Lysyl oxidase homolog 2 | LOXL2 (Q9Y4K0) | Enzyme involved in ECM maturation through crosslinking of collagen and elastin fibers | - | 1.09 |
| Fibrillin-1 | FBN1 (P35555) | Glycoprotein that plays a critical role in the formation and maintenance of elastic fibers in connective tissues. It is a major component of the ECM. | 2.06 | - |
| Cytokeratin 2 | KRT2 (P35908) | Intermediate filament protein that plays essential roles in providing structural support, promoting cell differentiation, and contributing to tissue organization in epithelial cells. | 3.31 | - |
| Proactivator polypeptide-like 1 | PSAPL1 (Q6NUJ1) | May be involved in lipid metabolism and intracellular trafficking. | 1.97 | - |
| Complement factor H | CFH (P08603) | Glycoprotein with an essential role in maintaining a well-balanced immune response by modulating complement activation. | - | -0.47 |
| Glyceraldehyde-3-phosphate dehydrogenase | GAPDH (P04406) | Important in cellular metabolism and regulation. Its involvement in glycolysis ensures the production of energy, while its non-metabolic functions contribute to cellular homeostasis and signaling. | - | -1.11 |
| Cytokeratin 1 | KRT1 (P04264) | Plays fundamental roles in the structural integrity, differentiation, and barrier function. May regulate the activity of kinases such as PKC and SRC. | 1.33 | -1.13 |
| Heat shock protein HSP 90β | HSP90AB1 (P08238) | Molecular chaperone that promotes the maturation, structural maintenance and proper regulation of specific target proteins involved for instance in cell cycle control and signal transduction. Undergoes a functional cycle that is linked to its ATPase activity. | 0.61 | -1.65 |
| Cytokeratin 10 | KRT10 (P13645) | Plays crucial roles in the structural integrity, differentiation, and barrier function of the epidermis. | 1.05 | -1.48 |
| Galectin-3-binding protein | LGALS3BP (Q08380) | Glycoprotein that interacts with various molecules in the extracellular matrix and cell surface. Can be involved in in immune regulation, inflammation, cell adhesion, and tissue remodeling. | 1.40 | -4.20 |

**Supplementary Table 6.** List of proteins with significant differential expression between heterotypic spheroids of HCT116/HCT116-DoxR and 2D models of fibroblasts derived EVs. Expression was assessed using Tukey's test (FDR of 0.05). Positive values indicate an increased expression in EVs from culture spheroids and Negative values indicate an increased expression in EVs from 2D models of fibroblasts.

| Protein | Abbreviation (ID Uniprot) | Description | T-student Test Difference | |
| --- | --- | --- | --- | --- |
|  |  |  | (HCT116-DoxR heterotypic spheroids –Fibroblasts 2D) | (HCT116-DoxR heterotypic spheroids –Fibroblasts 2D) |
| Titin | TTN (Q8WZ42) | Structural protein predominantly found in muscle tissues. Titin plays several essential functions related to muscle contraction, elasticity, and structural integrity. | - | 2.80 |
| Complement factor H | CFH (P08603) | Glycoprotein with an essential role in maintaining a well-balanced immune response by modulating complement activation. | - | 0.31 |
| Hemoglobin subunit α | HBA2 (P69905) | Plays a crucial role in oxygen transport. | - | 3.79 |
| Glyceraldehyde-3-phosphate dehydrogenase | GAPDH (P04406) | Important in cellular metabolism and regulation. Its involvement in glycolysis ensures the production of energy, while its non-metabolic functions contribute to cellular homeostasis and signaling. | - | 1.16 |
| α-2-macroglobulin | A2M (P01023) | Glycoprotein that plays essential roles in regulating protease activity, modulating immune responses, and participating in various cellular processes. | - | 1.00 |
| Cytokeratin 2 | KRT2 (P35908) | Intermediate filament protein that plays essential roles in providing structural support, promoting cell differentiation, and contributing to tissue organization in epithelial cells. | - | 1.13 |
| Heat shock protein HSP 90β | HSP90AB1 (P08238) | Molecular chaperone that promotes the maturation, structural maintenance and proper regulation of specific target proteins involved for instance in cell cycle control and signal transduction. Undergoes a functional cycle that is linked to its ATPase activity. | - | 3.37 |
| Cytokeratin 10 | KRT10 (P13645) | Plays crucial roles in the structural integrity, differentiation, and barrier function of the epidermis. | - | 1.13 |
| Galectin-3-binding protein | LGALS3BP (Q08380) | Glycoprotein that interacts with various molecules in the extracellular matrix and cell surface. Can be involved in in immune regulation, inflammation, cell adhesion, and tissue remodeling. | - | 3.77 |
| Caspase recruitment domain-containing protein 8 | CARD8 (Q9Y2G2) | Plays important roles in various cellular processes, particularly in the regulation of inflammation and cell death. It is known to Inhibit NF-κB activation. | - | 0.84 |
| Tropomyosin α-4 chain | TPM4 (P67936) | Play important roles in the regulation of actin filament dynamics and cellular processes. | 1.83 | - |
| Cytokeratin 9 | KRT9 (P35527) | Plays fundamental roles in the structural integrity, differentiation, and barrier function. | 1.34 | - |
| Collagen α-2(I) chain | COL1A2 (P08123) | Crucial component of collagen type I. Collagen type I is a fibrous, structural protein found in various connective tissues, providing strength, support, and flexibility to ECM. | -3.27 | -2.95 |
| Thrombospondin-2 | THBS2 (P35442) | Adhesive glycoprotein that mediates cell-to-cell and cell-to-matrix interactions essential in tissue development, ECM organization and wound healing. | -1.83 | -1.39 |
| Collagen α-3(VI) chain | COL6A3 (P12111) | Critical component of collagen type VI. Its presence in the ECM provides support, elasticity, and stability to tissues, and its functions are essential for tissue development, homeostasis, and repair. | -0.81 | -1.46 |
| Zinc Finger CCCH-Type Containing 14 | ZC3H14 (G3V4R5) | RNA-binding protein that is involved in the regulation of post-transcriptional gene expression. | - | -5.09 |
| 72 kDa type IV collagenase | MMP2 (P08253) | Ubiquitinous metalloproteinase that plays critical roles in tissue remodeling, repair, and various physiological and pathological processes involving the ECM. Rupture. | -2.36 | -2.23 |
| Collagen α-1(I) chain | COL1A1 (P02452) | Key component of the extracellular matrix (ECM) with diverse functions, contributing to tissue strength, wound healing, bone formation, and cell signaling. | -3.22 | -3.38 |
| Fibronectin | FN1 (P02751) | Essential ECM protein involved in cell-matrix interactions | -7.94 | -8.13 |
| Thrombospondin-1 | THBS1 (P07996) | Adhesive glycoprotein that mediates cell-to-cell and cell-to-matrix interactions essential in tissue organization, repair, and homeostasis. | -7.62 | -6.43 |
| Fibulin-1 | FBLN1 (P23142) | Component of the ECM and is involved in the assembly and stabilization of various ECM structures. Its interactions with other ECM components and cell surface receptors make it a critical player in tissue development, homeostasis, and repair. | -2.17 | -1.84 |
| Fibrillin-1 | FBN1 (P35555) | Glycoprotein that plays a critical role in the formation and maintenance of elastic fibers in connective tissues. It is a major component of the ECM. | -3.59 | -2.39 |
| Peroxidasin homolog | PXDN (Q92626) | Displays peroxidase activity and is likely to participate in H_2_O_2_ metabolism. Plays a role in extracellular matrix formation | -1.60 | -1.91 |
| Lysyl oxidase homolog 2 | LOXL2 (Q9Y4K0) | Enzyme involved in ECM maturation through crosslinking of collagen and elastin fibers | -2.42 | -1.87 |
| Dermatan-sulfate epimerase | DSE (Q9UL01) | Enzyme involved in the biosynthesis of dermatan sulfate, a glycosaminoglycan with roles in tissue organization, function, and biological signaling. | -5.55 | - |

**Figures**

**Supplementary Figure 1.** Growth curve of fibroblasts cultured as 2D monolayers in DMEM supplemented with supplemented with 10% heat-inactivated fetal bovine serum, 100 U/mL penicillin and 0.1 mg/mL streptomycin and 5 ng/mL of FGF. Non-linear regression of the growth curve described fibroblasts growth as an exponential curve, following the equation Y=Y0*exp(b*X), with Y0=1890.3 and b=0.5079 and an R^2^=0.9897.

**Supplementary Figure 2.** Growth curve of HCT116 cells cultured as 2D monolayers in DMEM supplemented with supplemented with 10% heat-inactivated fetal bovine serum, 100 U/mL penicillin and 0.1 mg/mL streptomycin. Non-linear regression of the growth curve described HCT116 cells growth as an exponential curve, following the equation Y=Y0*exp(b*X), with Y0=1254.7 and b=1.1474 and an R^2^=0.9918.

**Supplementary Figure 3.** Growth curve of HCT116-DoxR cells cultured as 2D monolayers in DMEM supplemented with supplemented with 10% heat-inactivated fetal bovine serum, 100 U/mL penicillin and 0.1 mg/mL streptomycin. Non-linear regression of the growth curve described HCT116-DoxR cells growth as an exponential curve, following the equation Y=Y0*exp(b*X), with Y0=1415.5 and b=0.9432 and an R^2^=0.9974.


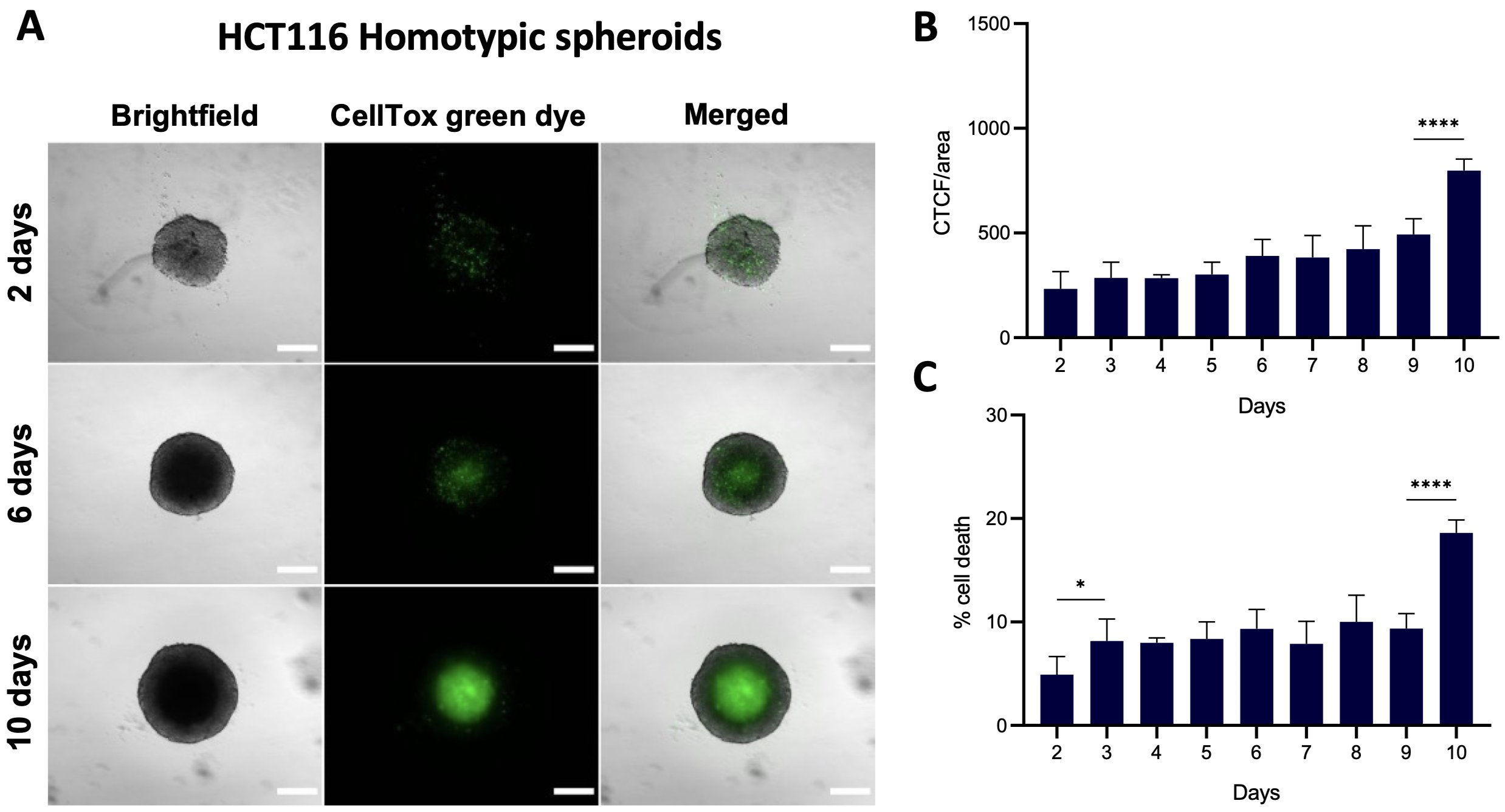


**Supplementary Figure 4.** Cell viability in HCT116 homotypic spheroids. **(A)** Fluorescence microscopy images of HCT116 homotypic spheroids with 2, 6, and 10 days of growth. **(B)** CTCF/area values and **(C)** percentage of cell death for HCT116 homotypic spheroids between 2 and 10 days of growth. Spheroids were incubated with CellTox^TM^ Green dye 1x for 24 h. Scale bars correspond to 300 μm. Data expressed as the mean ± SD of at least two independent assays. Statistical analysis was performed by two-way ANOVA method (**p* <0.1, *****p*<0.0001).


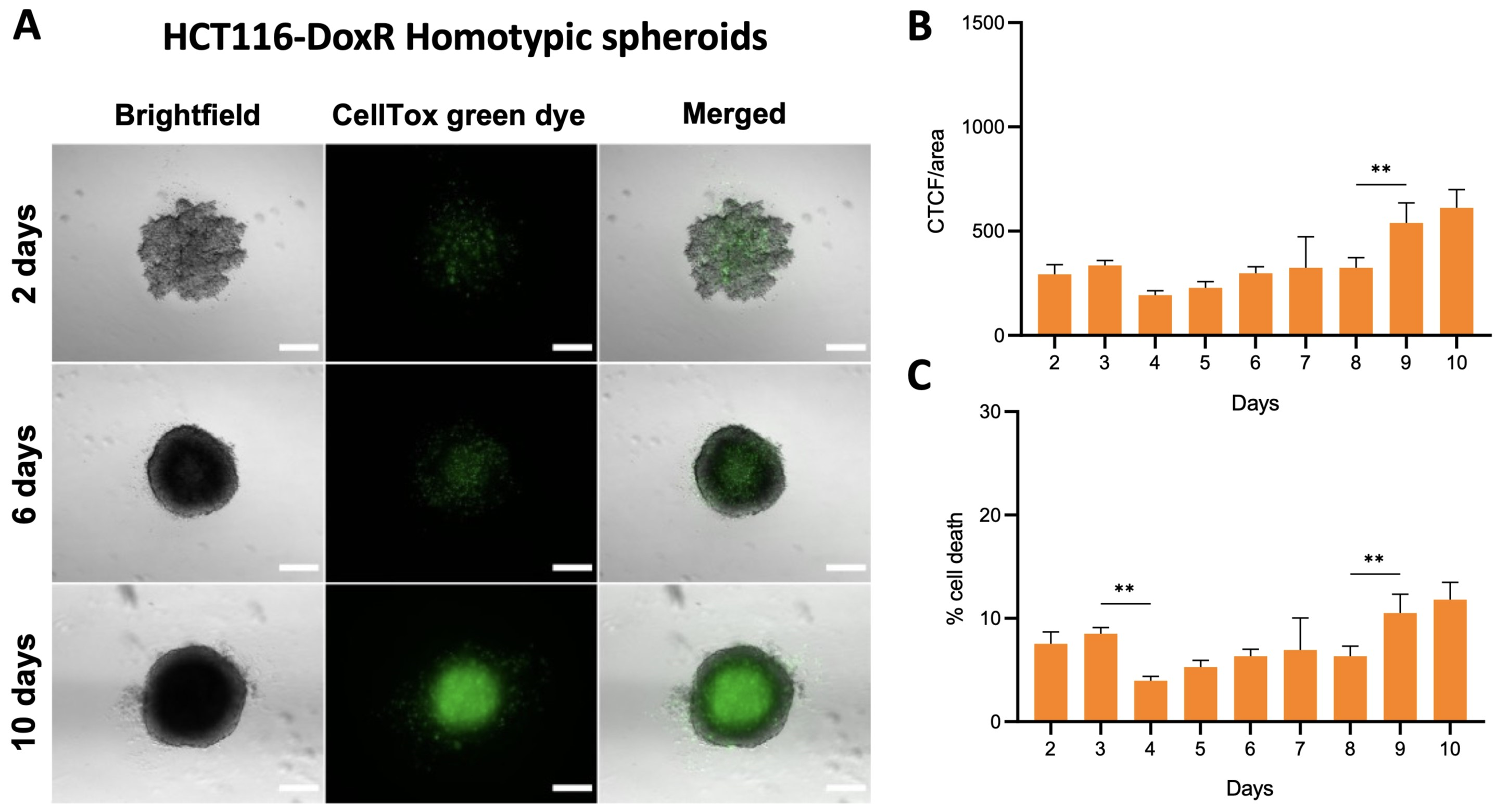


**Supplementary Figure 5.** Cell viability in HCT116-DoxR homotypic spheroids. **(A)** Fluorescence microscopy images of HCT116-DoxR homotypic spheroids with 2, 6, and 10 days of growth. **(B)** CTCF/area values and **(C)** percentage of cell death for HCT116-DoxR homotypic spheroids between 2 and 10 days of growth. Spheroids were incubated with CellTox^TM^ Green dye 1x for 24 h. Scale bars correspond to 300 μm. Data expressed as the mean ± SD of at least two independent assays. Statistical analysis was performed by two-way ANOVA method (***p*<0.01, *****p*<0.0001).


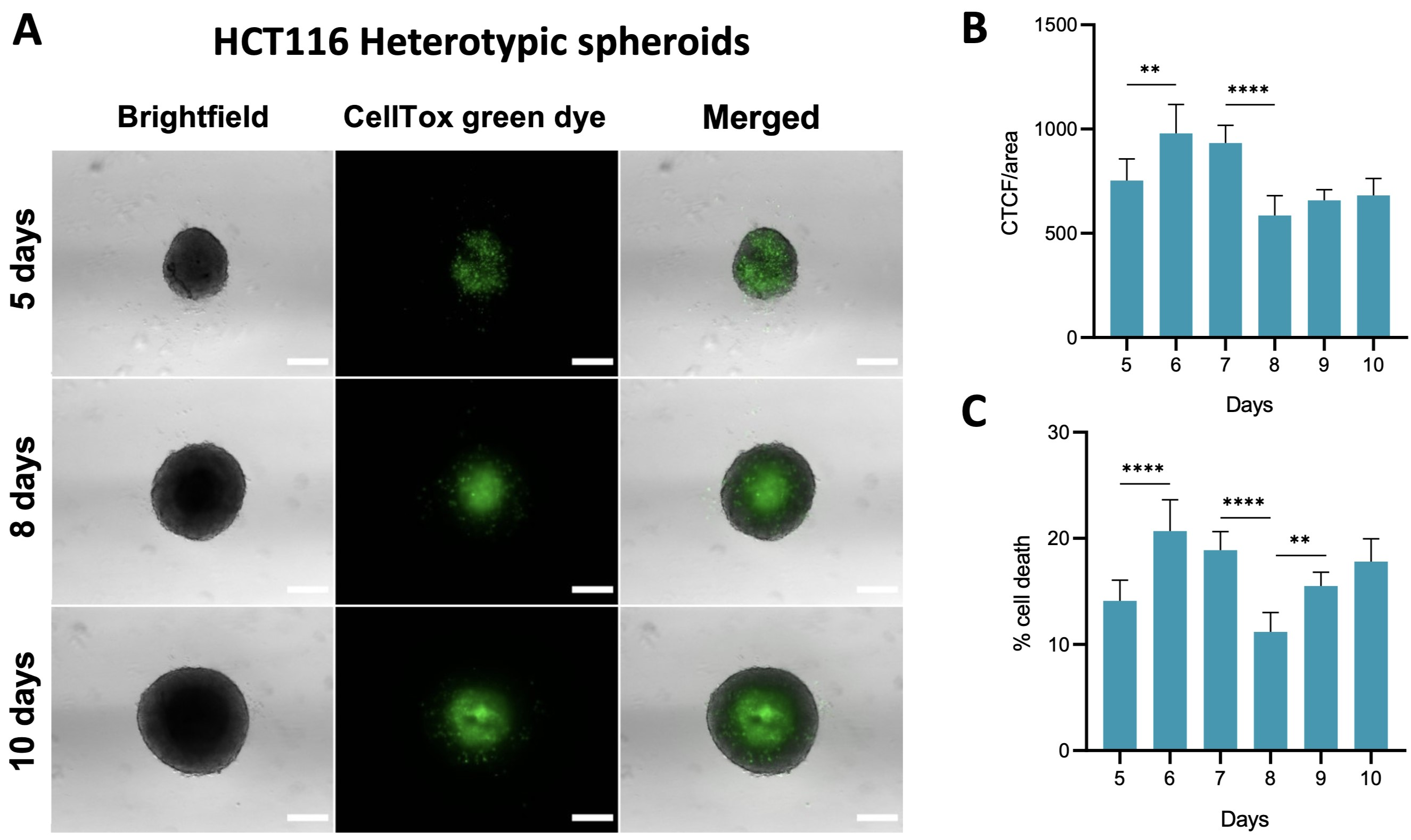


**Supplementary Figure 6.** Cell viability in HCT116 heterotypic spheroids. **(A)** Fluorescence microscopy images of HCT116 heterotypic spheroids with 5, 8, and 10 days of growth. **(B)** CTCF/area values and **(C)** percentage of cell death for HCT116 heterotypic spheroids between 5 and 10 days of growth. Spheroids were incubated with CellTox^TM^ Green dye 1x for 24h. Scale bars correspond to 300 μm. Data expressed as the mean ± SD of at least two independent assays. Statistical analysis was performed by two-way ANOVA method (***p*<0.01, *****p*<0.0001).


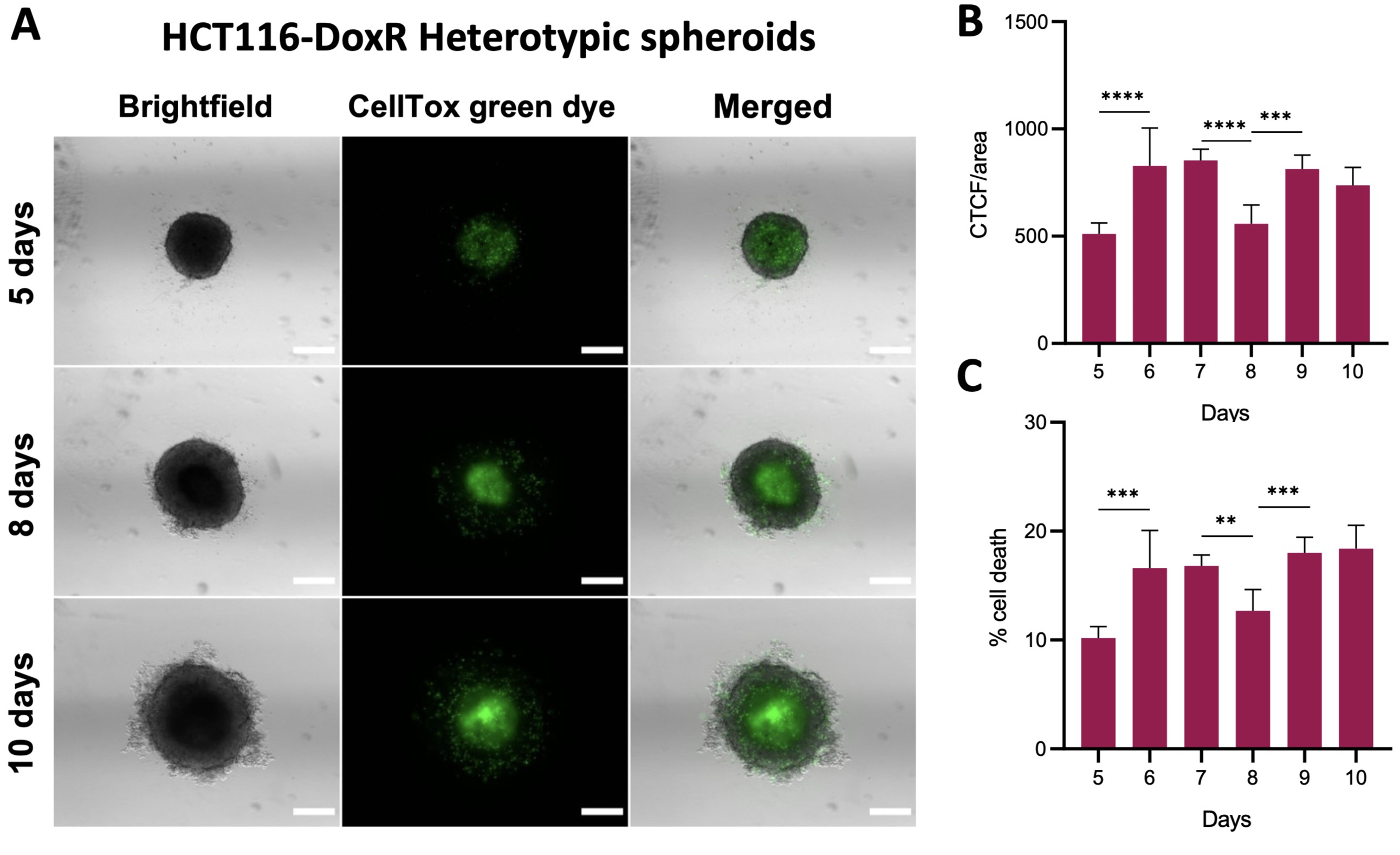


**Supplementary Figure 7.** Cell viability in HCT116-DoxR heterotypic spheroids. **(A)** Fluorescence microscopy images of HCT116-DoxR heterotypic spheroids with 5, 8, and 10 days of growth. **(B)** CTCF/area values and **(C)** percentage of cell death for HCT116-DoxR heterotypic spheroids between 5 and 10 days of growth. Spheroids were incubated with CellTox^TM^ Green dye 1x for 24h. Scale bars correspond to 300 μm. Data expressed as the mean ± SD of at least two independent assays. Statistical analysis was performed by two-way ANOVA method (***p*<0.01, ****p*<0.001, *****p*<0.0001).


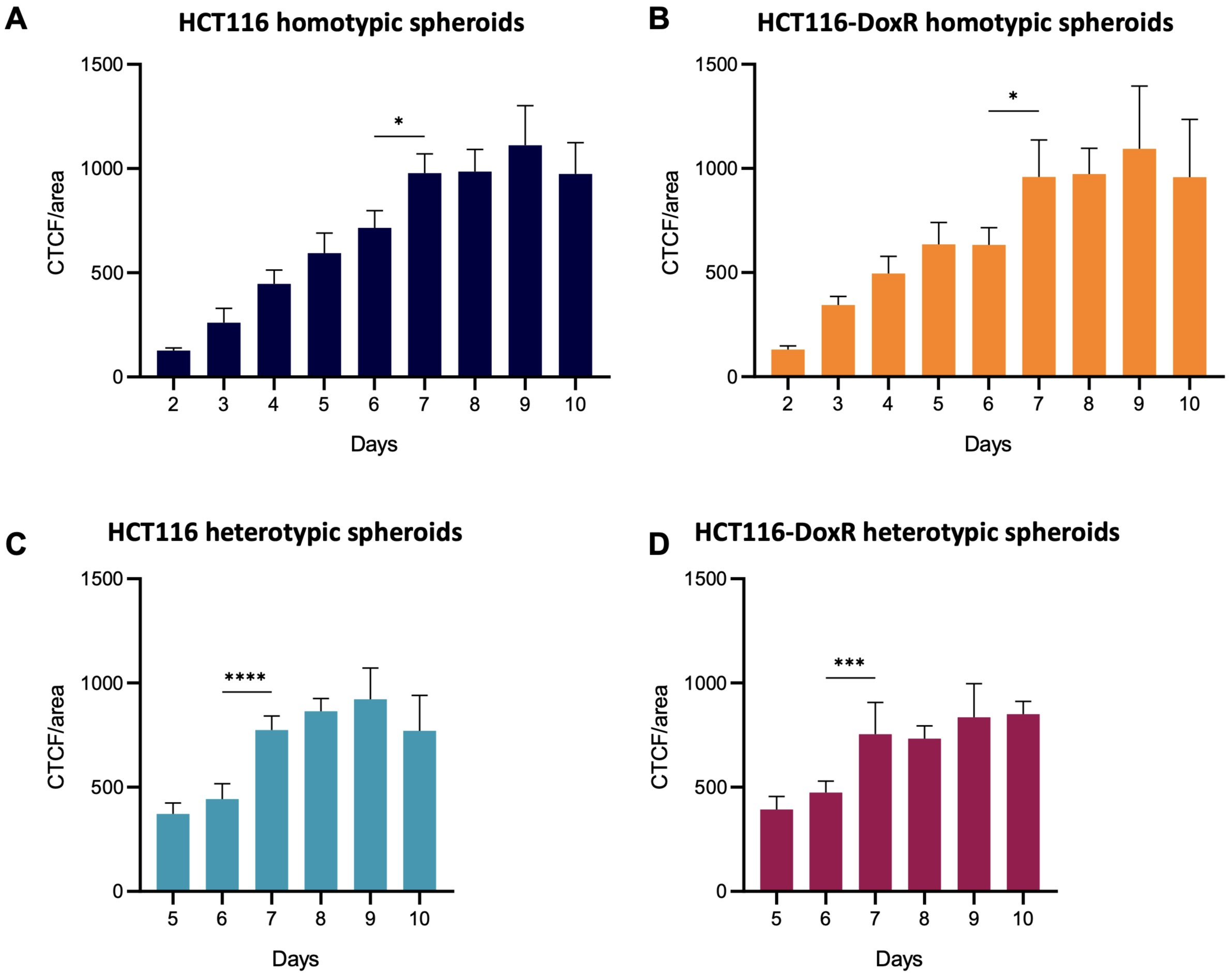


**Supplementary Figure 8.** Hypoxia levels in HCT116/HCT116-DoxR homotypic and heterotypic spheroids. CTCF/area values for hypoxia in HCT116 **(A)** and HCT116-DoxR **(B)** homotypic spheroids between 2 and 10 days of growth, and in HCT116 **(C)** and HCT116-DoxR **(D)** heterotypic spheroids between 5 and 10 days of growth. Spheroids were incubated with Image-iTTM Red Hypoxia Reagent for 24 h. Data expressed as the mean ± SD of at least two independent assays. Statistical analysis was performed by two-way ANOVA method (**p*<0.1, ***p*<0.01, ****p*<0.001, *****p*<0.0001).


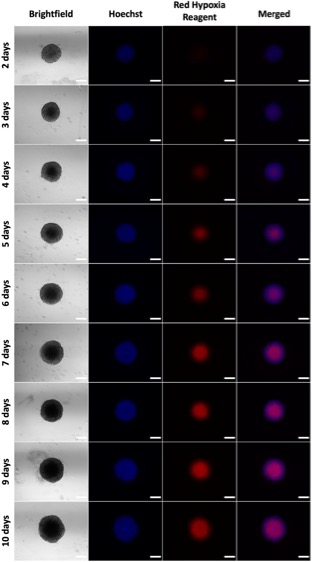


**Supplementary Figure 9.** Fluorescence microscopy images of HCT116 homotypic spheroids between 2 and 10 days of growth. Spheroids were incubated with Image-iT^TM^ Red Hypoxia Reagent and Hoechst 33258 for 24 h. It is possible to observe the accumulation of hypoxia in the center of spheroids since the 4th day of growth. Scale bars correspond to 300 μm.


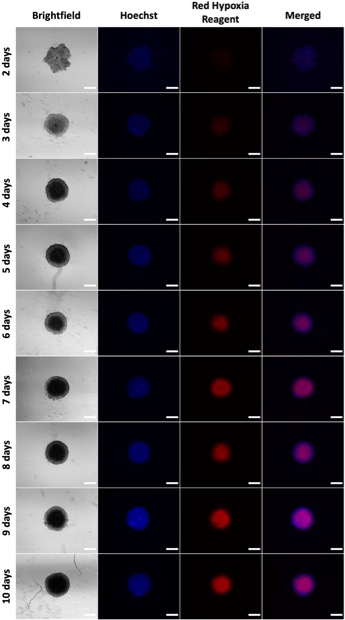


**Supplementary Figure 10.** Fluorescence microscopy images of HCT116-DoxR homotypic spheroids between 2 and 10 days of growth. Spheroids were incubated with Image-iT^TM^ Red Hypoxia Reagent and Hoechst 33258 for 24h. It is possible to observe the accumulation of hypoxia in the center of spheroids since the 4th day of growth. Scale bars correspond to 300 μm.


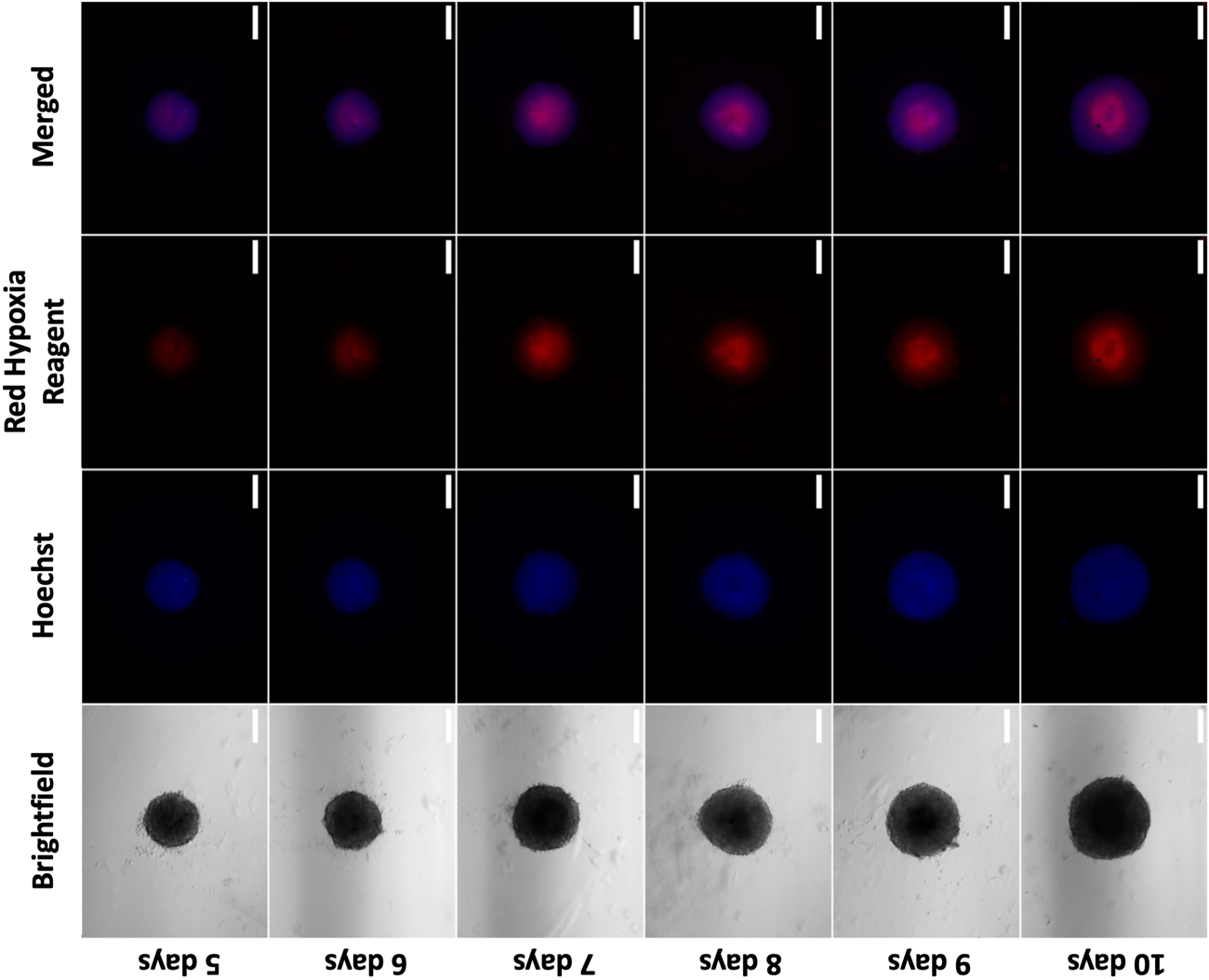


**Supplementary Figure 11.** Fluorescence microscopy images of HCT116 heterotypic spheroids between 5 and 10 days of growth. Spheroids were incubated with Image-iTTM Red Hypoxia Reagent and Hoechst 33258 for 24h. It is possible to observe the accumulation of hypoxia in the center of spheroids since the 6th day of growth. Scale bars correspond to 300 μm.


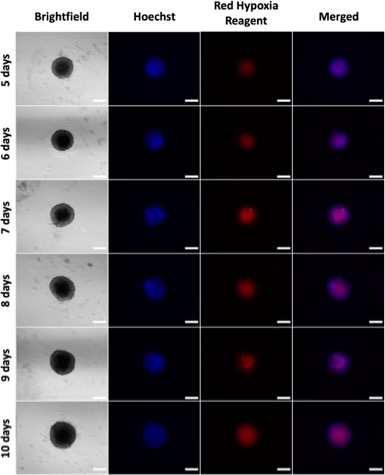


**Supplementary Figure 12.** Fluorescence microscopy images of HCT116-DoxR heterotypic spheroids between 5 and 10 days of growth. Spheroids were incubated with Image-iT^TM^ Red Hypoxia Reagent and Hoechst 33258 for 24h. It is possible to observe the accumulation of hypoxia in the center of spheroids since the 6th day of growth. Scale bars correspond to 300 μm.


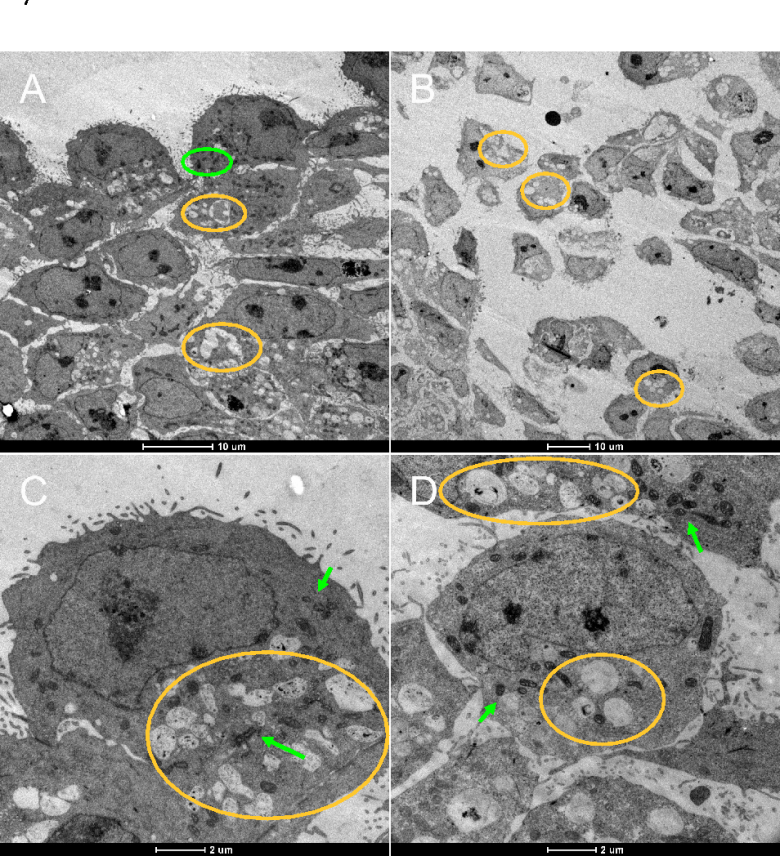


**Supplementary Figure 13.** TEM images of HCT116 heterotypic spheroids, from **(A, C)** the outer layer and **(B, D)** the core. Green arrows and circles indicate the presence of mitochondria. Yellow circles point out vesicles. Scale bars correspond to **(A, B)** 10 µm or **(C, D)** 2 µm.


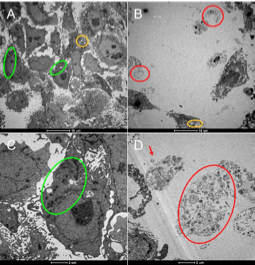


**Supplementary Figure 14.** TEM images of HCT116-DoxR heterotypic spheroids, from **(A, C)** the outer layer and **(B, D)** the core. Green circles indicate the presence of mitochondria. Yellow circles point out vesicles. Red arrows and circles denote dead cells and cell debris. Scale bars correspond to **(A, B)** 10 µm or **(C, D)** 2 µm.


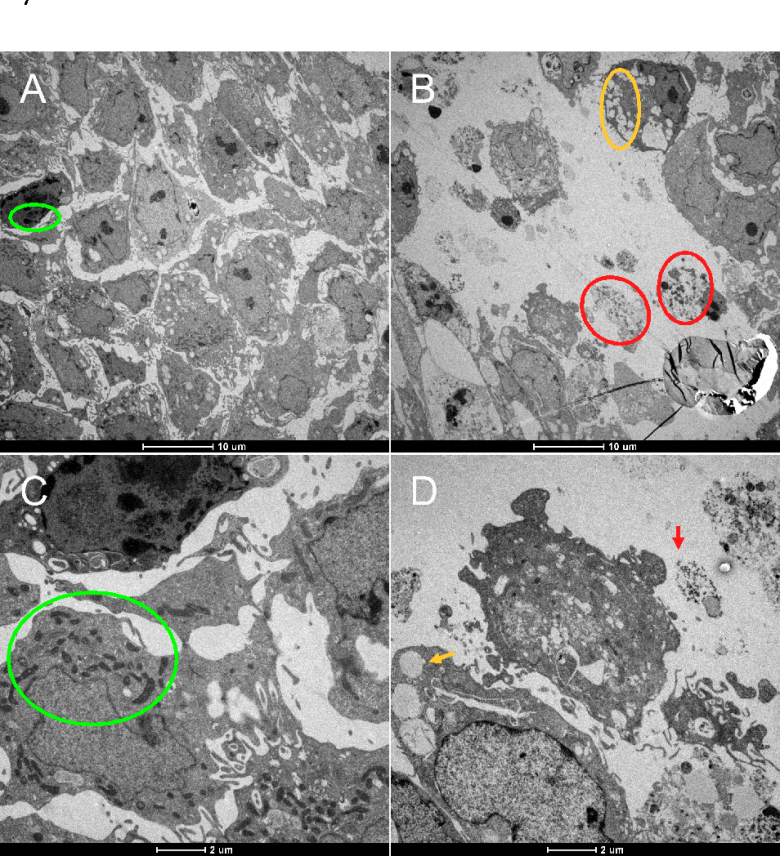


**Supplementary Figure 15.** TEM images of HCT116-DoxR homotypic spheroids, from **(A, C)** the outer layer and **(B, D)** the core. Green circles indicate the presence of mitochondria. Yellow arrows and circles point out vesicles. Red arrows and circles denote dead cells and cell debris. Scale bars correspond to **(A, B)** 10 µm or **(C, D)** 2 µm.


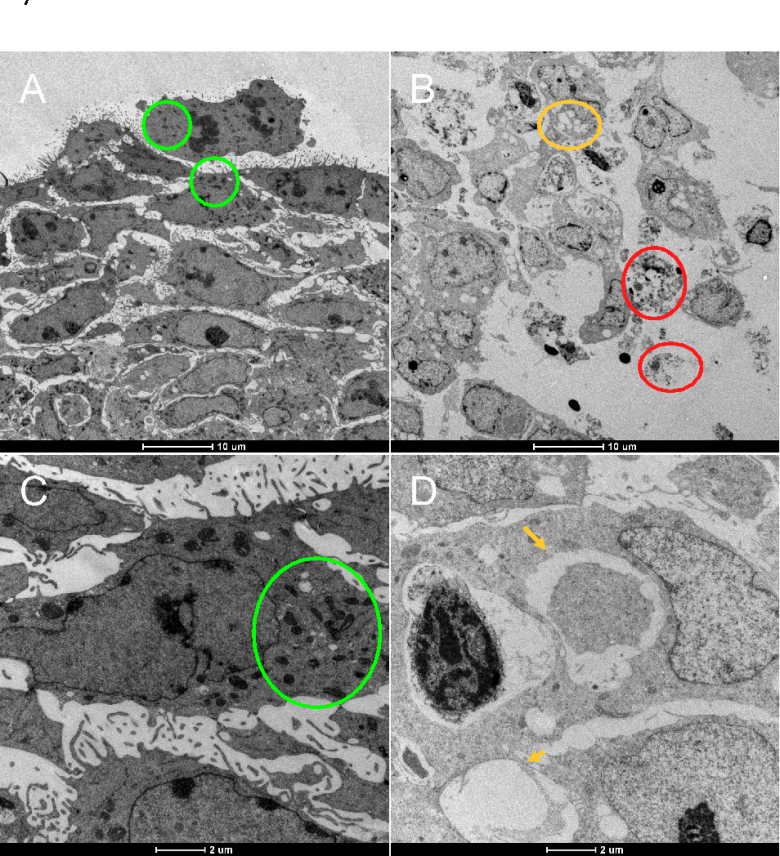


**Supplementary Figure 16.** TEM images of HCT116 homotypic spheroids, from **(A, C)** the outer layer and **(B, D)** the core. Green circles indicate the presence of mitochondria. Yellow arrows and circles point out vesicles. Red circles denote dead cells and cell debris. Scale bars correspond to **(A, B)** 10 µm or **(C, D)** 2 µm.


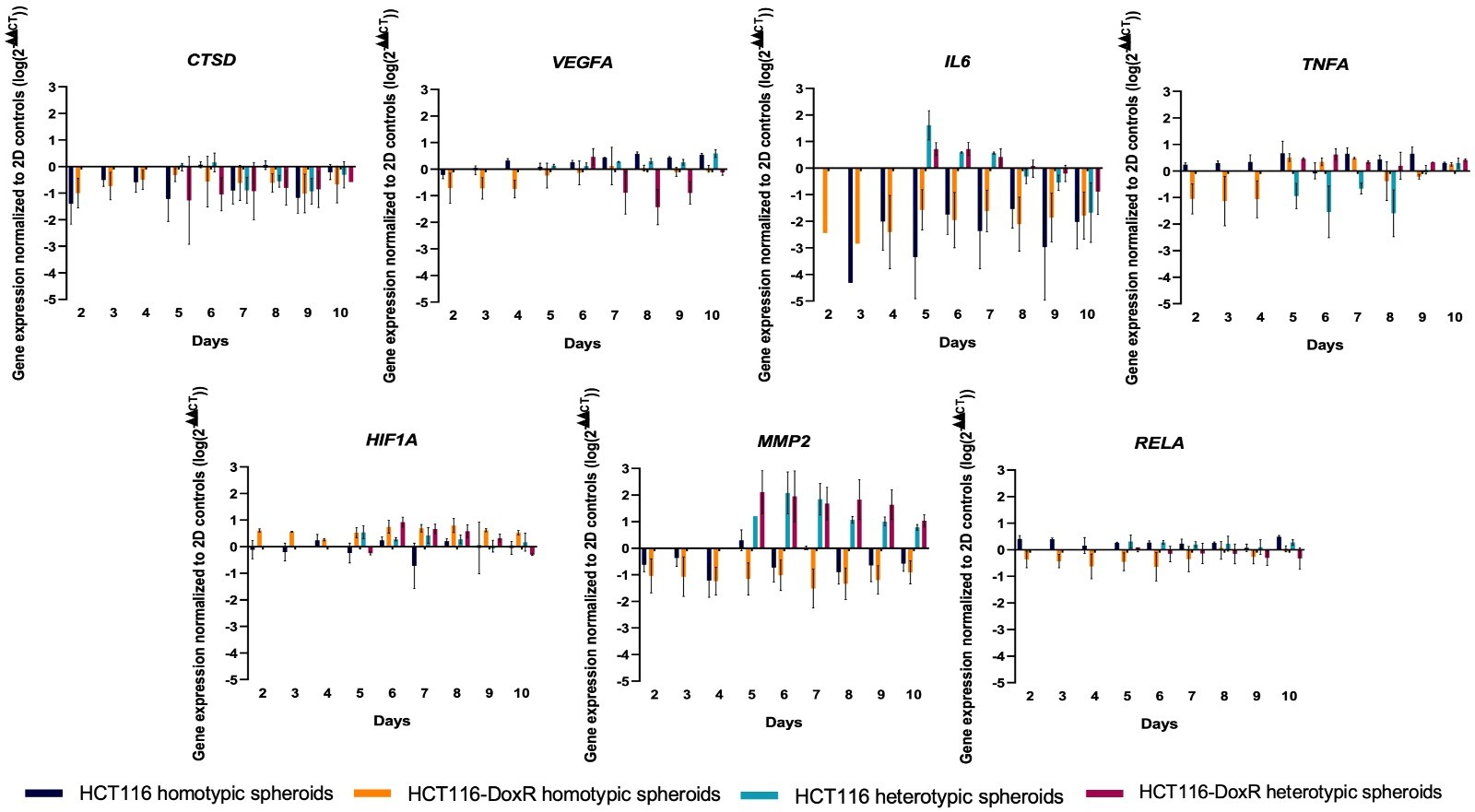


**Supplementary Figure 17.** Gene expression of *HIF1A*, *RELA*, *VEGFA*, *MMP2*, *CTSD*, *IL6* and *TNFA* in different types of spheroids, HCT116 homotypic and heterotypic and HCT116-DoxR homotypic and heterotypic, for 2-10 days of growth, in the case of the homotypic spheroids, and 5-10 days of growth, in the case of heterotypic spheroids. All data was normalized to the gene expression of its correspondent 2D culture (HCTT116 or HCT116-DoxR cell lines). The value 0 is considered as basal expression and the red color represents overexpression and the blue color under expression of the genes. Data expressed as the mean ± SEM of at least two independent assays.


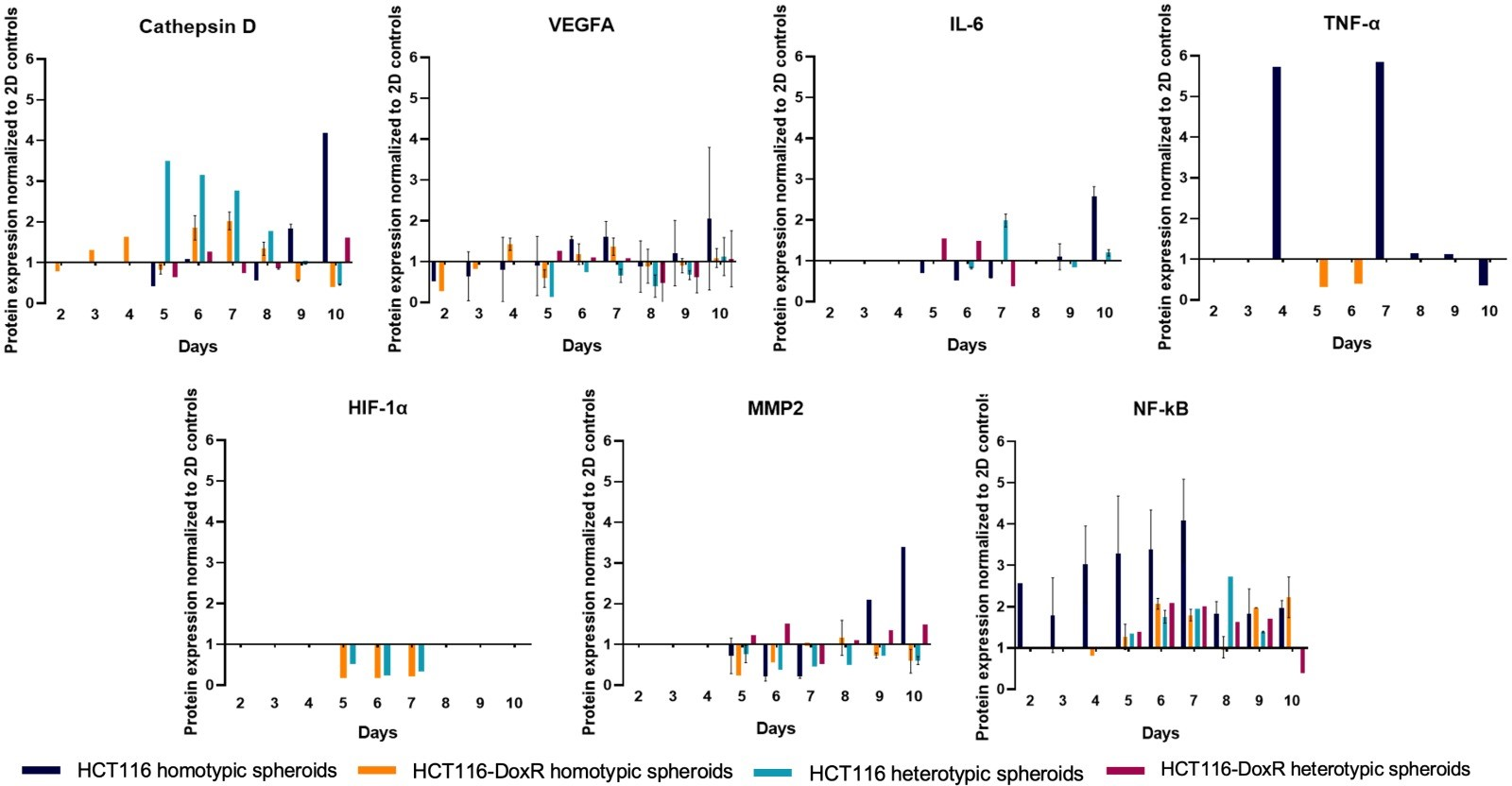


**Supplementary Figure 18.** Protein levels of HIF-1α, TNF-α, NF-κB p65, IL-6, VEGFA, MMP2 and Cathepsin D in different types of spheroids, HCT116 homotypic and heterotypic and HCT116-DoxR homotypic and heterotypic, for 2-10 days of growth, in the case of the homotypic spheroids, and 5-10 days of growth, in the case of heterotypic spheroids. All data was normalized to the protein expression of its correspondent 2D culture (HCTT116 or HCT116-DoxR cell lines). Value 1 is considered as basal expression and the red color represents overexpression and the blue color under expression of the proteins. Data expressed as the mean ± SEM of at least two independent assays.


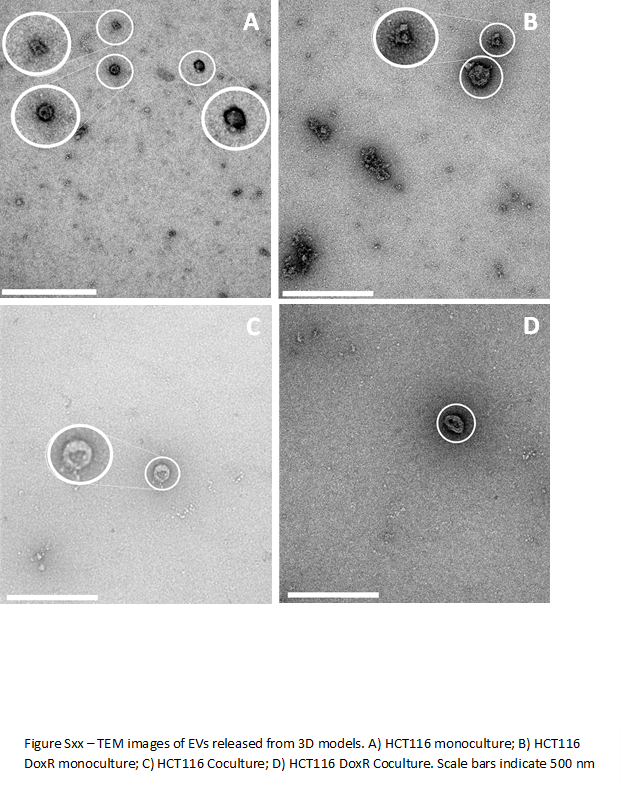


**Supplementary Figure 19**. TEM images of EVs released from 3D models. A) HCT116 homotypic spheroids; B) HCT116-DoxR homotypic spheroids; C) HCT116 heterotypic spheroids; D) HCT116-DoxR heterotypic spheroids. Scale bars indicate 500 nm.


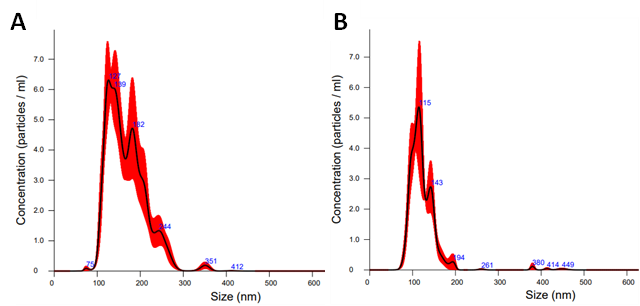


**Supplementary Figure 20**. NTA profiles of EVs released by 3D homotypic spheroids (A- HCT116; B- HCT116-DoxR). The samples were prepared with a dilution of 1:10000.


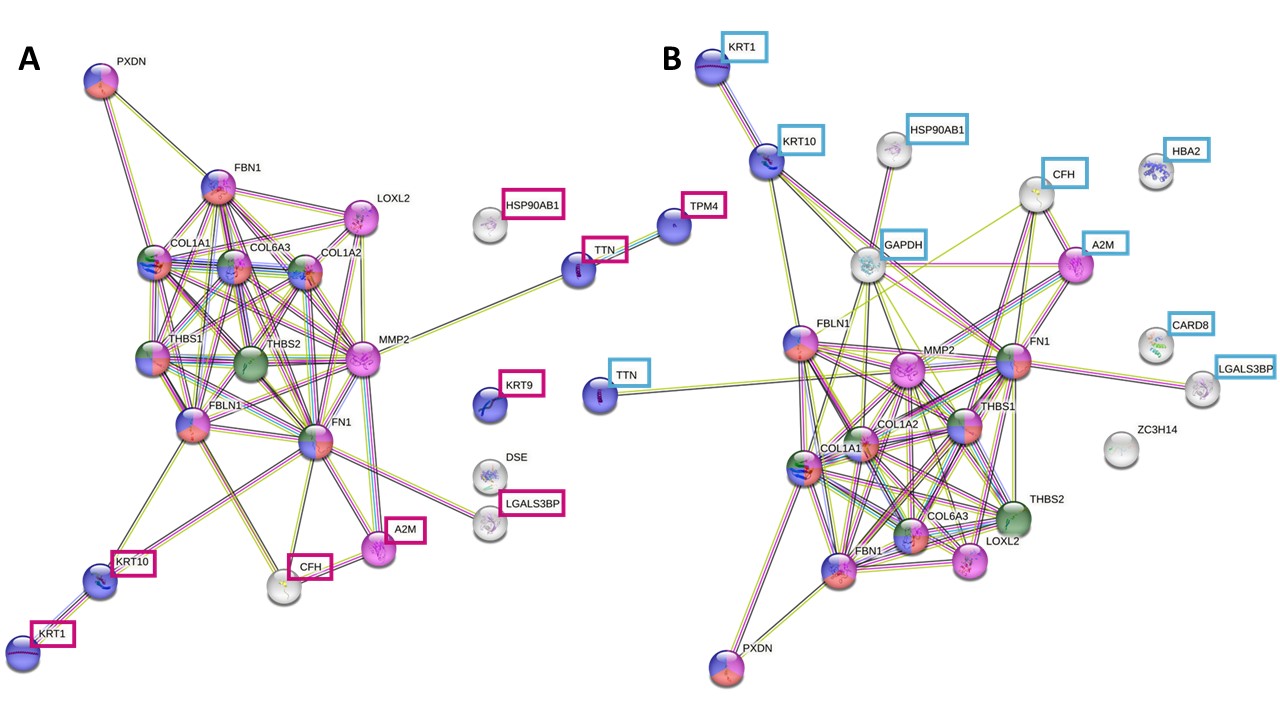


**Supplementary Figure 21**. Proteins with altered expression in EVs isolated from HCT116-DoxR homotypic spheroids and 2D fibroblasts models **(A)** and from HCT116 heterotypic spheroids and fibroblast 2D models **(B)**. Nodes represent proteins and the lines connecting them indicate direct or indirect interactions. Blue nodes indicate a structural molecule activity (Molecular function – [GO:0005198](http://amigo.geneontology.org/amigo/term/GO:0005198)); red nodes indicate ECM structural constituents (Molecular function – [GO:0005201](http://amigo.geneontology.org/amigo/term/GO:0005201)); the purple nodes indicate proteins with a role in ECM organization (Biological process - [GO:0030198](http://amigo.geneontology.org/amigo/term/GO:0030198)); the green nodes indicate proteins involved in ECM-receptor interaction (KEGG Pathways - [hsa04512](https://www.kegg.jp/kegg-bin/show_pathway?hsa04512)) and white nodes indicates proteins without a biological process associated. Proteins marked with the purple and cyan boxes presented an increased expression in EVs extracted from HCT116-DoxR heterotypic spheroids or HCT116 heterotypic spheroids, respectively.


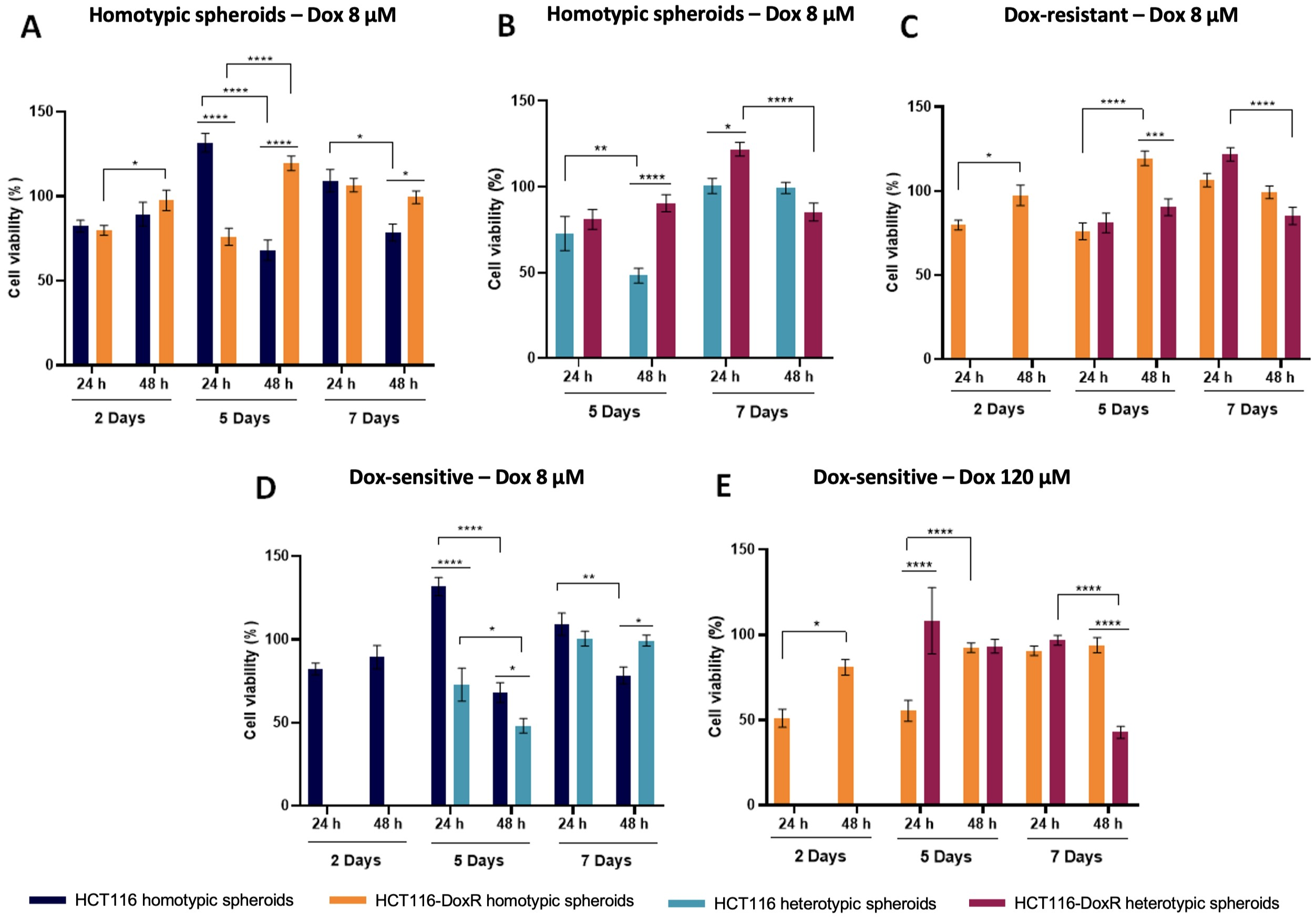


**Supplementary Figure 22.** Percentage of cell viability of different types of spheroids after exposure to 8 µM or 120 µM of Dox for 24 h or 48 h. Cell viability data was compared between **(A)** homotypic or **(B)** heterotypic spheroids after 24 h and 48 h incubation with 8 µM Dox; homotypic and heterotypic **(C)** resistant or **(D)** sensitive spheroids after 24 h and 48h incubation with 8 µM Dox; and **(E)** homotypic and heterotypic resistant spheroids after 24 h and 48 h incubation with 120 µM Dox. DMSO was used as the vehicle control. Data are expressed as mean ± SEM of two independent assays (* *p* < 0.1, ** *p* < 0.01, *** *p* < 0.001, **** *p* < 0.0001), Statistical analysis was performed by two-way ANOVA method.

**Supplementary Figure 23.** Percentage of cell viability of fibroblasts cell line after 24 h exposure to different concentration of Dox. 0.1 % (v/v) DMSO was used as vehicle control. Data are expressed as mean ± SEM of at least two independent assays.


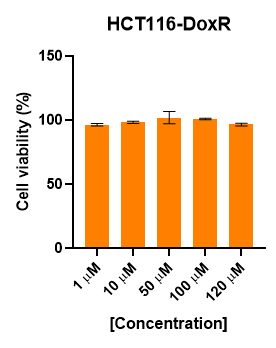


**Supplementary Figure 24.** Percentage of cell viability of HCT116-DoxR tumor cell line after 24 h exposure to different concentration of Dox. 0.1 % (v/v) DMSO was used as vehicle control. Data are expressed as mean ± SEM of two independent assays.


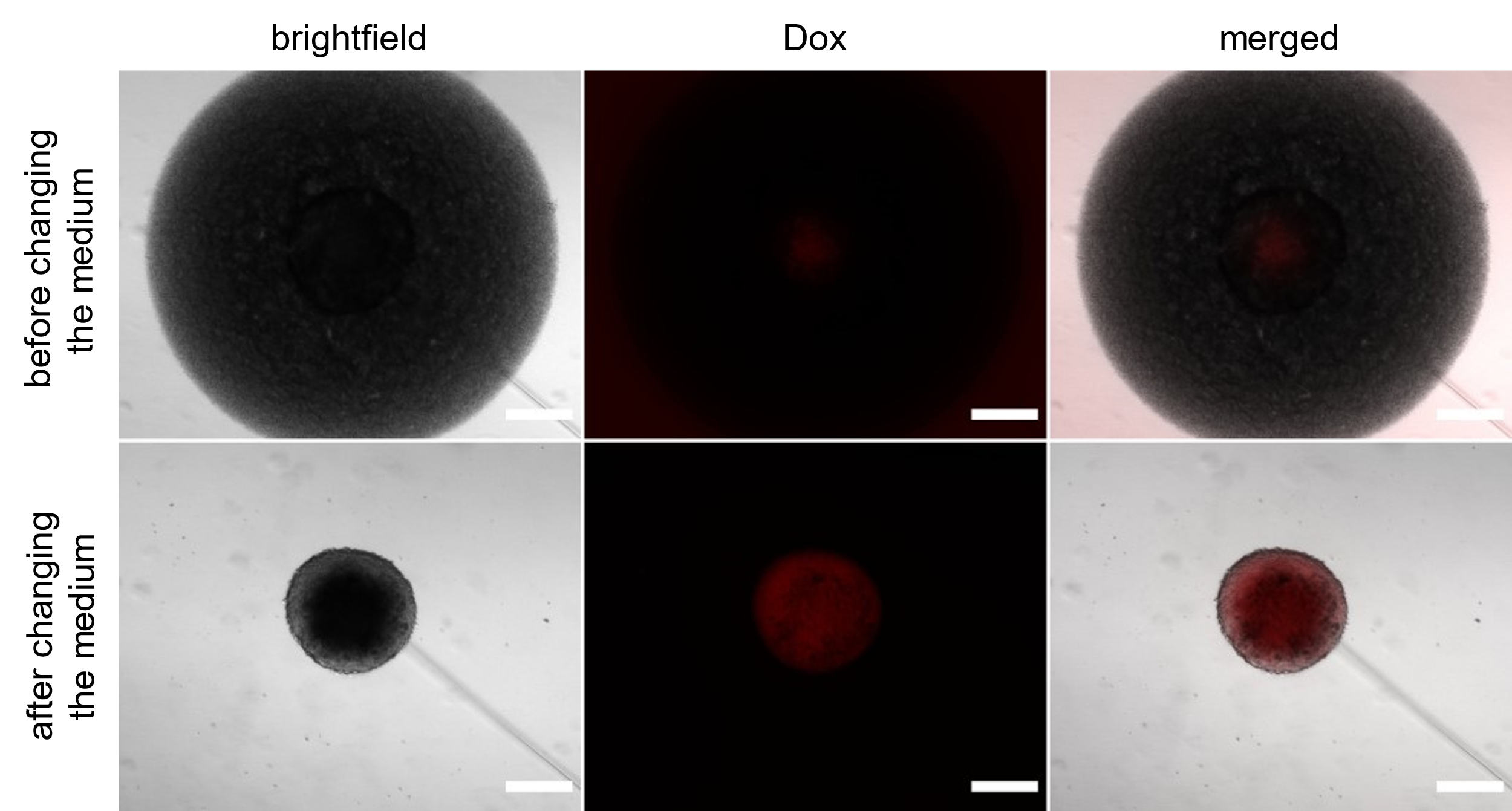


**Supplementary Figure 25.** Effect of incubation with 120 µM Dox in spheroids. In all spheroids incubated with 120 µM Dox, a sphere of probably dead cells was observed around the spheroid, which was removed after changing the culture medium by fresh medium. This is an example of an HCT116-DoxR homotypic spheroid with 10 days of growth after incubation with 120 µM Dox for 24h. Scale bars correspond to 300 µm.


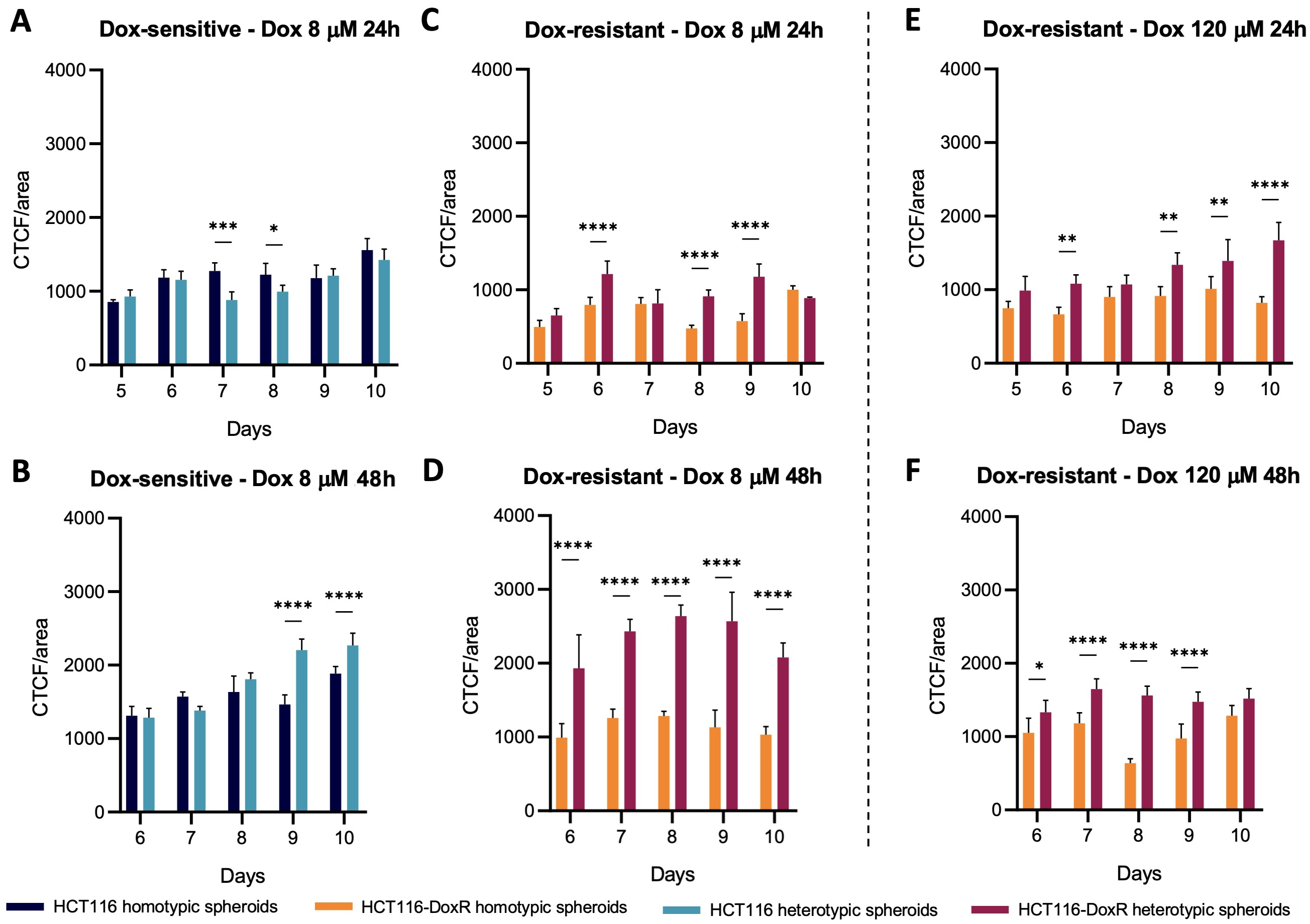


**Supplementary Figure 26.** Comparison of Dox internalization between Dox-sensitive and Dox-resistant spheroids. CTCF/area values were compared between **(A, B)** Dox-sensitive or **(C, D)** Dox-resistant spheroids after 24 h or 48 h incubation with 8 µM Dox, and between **(E, F)** Dox-resistant spheroids after 24 h or 48 h incubation with 120 µM Dox. Data expressed as the mean ± SD of at least two independent assays. Statistical analysis was performed by two-way ANOVA method (**p*<0.1, ***p*<0.01, ****p*<0.001, *****p*<0.0001).


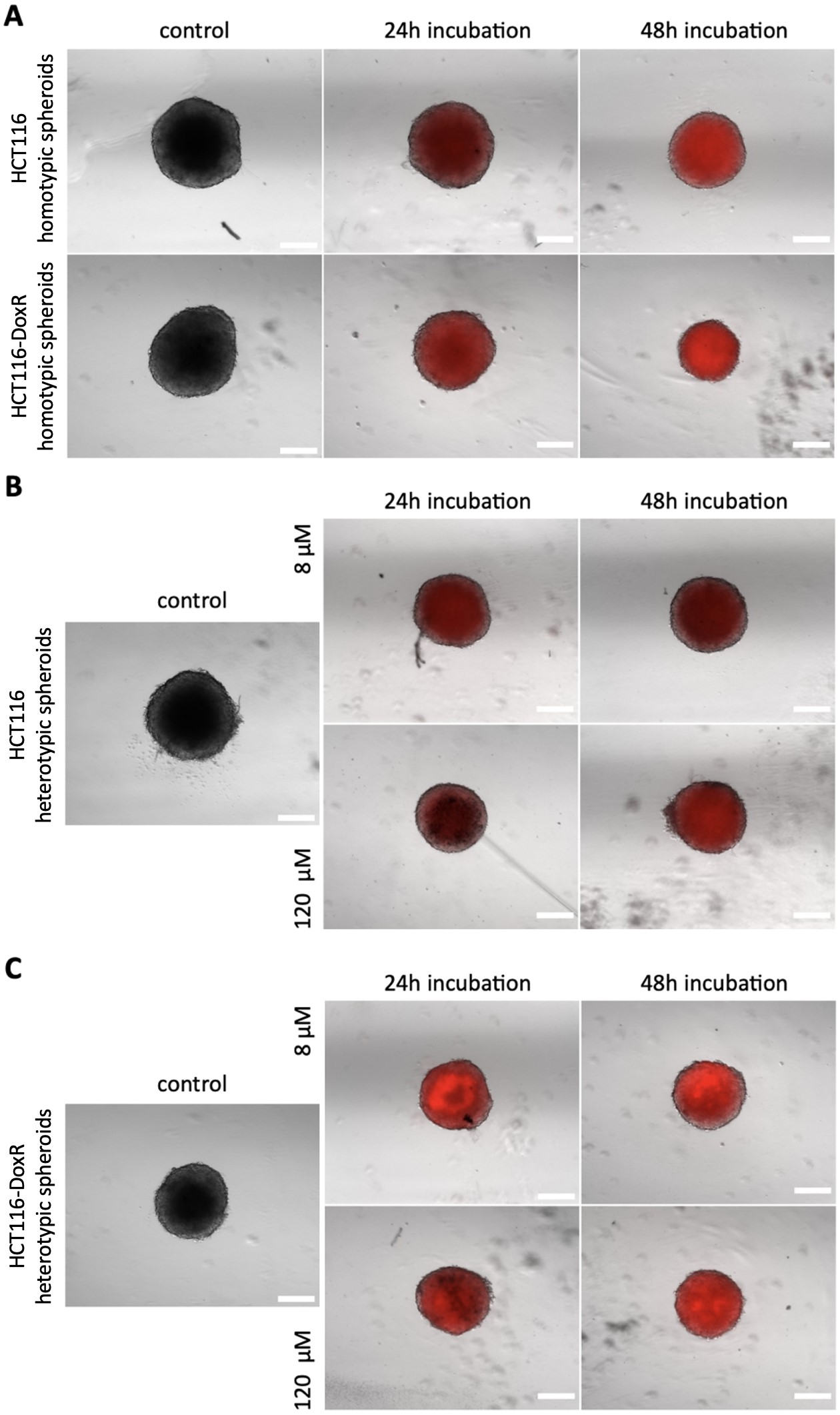


**Supplementary Figure 27.** Variation of spheroids volume after incubation with Dox. Fluorescence microscopy images of spheroids with 10 days of growth after incubation with Dox. **(A)** Comparison of HCT116 homotypic and heterotypic spheroids’ size after 24 h or 48 h incubation with 8 µM Dox. **(B)** Comparison of HCT116-DoxR homotypic spheroids’ size after 24 h or 48 h incubation with 8 or 120 µM Dox. **(C)** Comparison of HCT116-DoxR heterotypic spheroids’ size after 24 h or 48 h incubation with 8 or 120 µM Dox. Scale bars correspond to 300 µm.

**Videos**

**Supplementary video 1.** Real time formation and growth of HCT116 homotypic spheroid.

**Supplementary video 2.** Real time formation and growth of HCT116-DoxR homotypic spheroid.

**Supplementary video 3.** Real time distribution of fibroblasts in 3D heterotypic spheroids with HCT116.

**Supplementary video 4.** Real time distribution of fibroblasts in 3D heterotypic spheroids with HCT116-DoxR.

**Supplementary videos 5.** EVs samples released by HCT116 3D homotypic spheroids analyzed by NTA. The samples were prepared with a dilution of 1:10000.

**Supplementary videos 6.** EVs samples released by HCT116-DoxR 3D homotypic spheroids analyzed by NTA. The samples were prepared with a dilution of 1:10000.
